# Changes in psychotropic medication prescription patterns during the COVID-19 pandemic among Japanese children, adolescents and young adults: interrupted time-series study using a national claims database

**DOI:** 10.1192/bjo.2025.10903

**Published:** 2025-11-14

**Authors:** Wenbo Huang, Hiroki Matsui, Yusuke Sasabuchi, Hideo Yasunaga

**Affiliations:** Department of Clinical Epidemiology and Health Economics, School of Public Health, Graduate School of Medicine, The University of Tokyo, Tokyo, Japan; Department of Real-world Evidence, Graduate School of Medicine, The University of Tokyo, Tokyo, Japan

**Keywords:** COVID-19, psychotropic medication, children, adolescents

## Abstract

**Background:**

To date, no research has explored the impact of the COVID-19 pandemic on psychotropic prescription patterns among young people in Japan, where lockdown measures were relatively less stringent.

**Aims:**

This study aimed to investigate changes in the prescription patterns of psychotropic medications for Japanese young people before and after the COVID-19 pandemic, using the DeSC Database.

**Method:**

We conducted an interrupted time-series analysis, with data from February 2016 to November 2022, to assess the pandemic’s effects on psychotropic prescriptions for children, adolescents and young adults. The analysis included subgroups based on age (6–11, 12–17 and 18–22 years) and gender. The number of patients prescribed psychiatric drugs before and after the pandemic was analysed.

**Results:**

Among 93 385 individuals, psychotropic prescriptions – except anxiolytics – increased overall, although not uniformly across age and gender groups. Significant upward trends were observed in the prescription rates of antidepressants (from 2.53 (95% CI 2.21–2.84) to 6.47 (95% CI 5.89–7.05) patients per month), anxiolytics (from −1.83 (95% CI −2.52 to −1.13) to 7.37 (95% CI 6.06–8.67) per month) and hypnotics/sedatives (from −1.48 (95% CI 0.73–2.24) to 6.62 (95% CI 5.21–8.03) per month).

**Conclusions:**

A persistent increase in psychotropic medication prescriptions was observed after the COVID-19 pandemic. Given the influence of age and gender, clinicians and society must prioritise the mental health needs of the female and adolescent populations. These findings may be generalisable to other countries that implemented less stringent lockdown measures.

Mental health disorders and psychological issues represent some of the most serious challenges in paediatric healthcare. By 18 years of age, approximately 15% of children and teenagers are projected to experience a mental illness.^1^ In Japanese youth, social isolation and suicidal behaviour attributable to mental health disorders have long been major concerns.^2,3^ The global spread of the COVID-19 worsened mental health issues, introducing stressors such as social isolation and economic uncertainty.^4–7^ These stressors, along with anxieties about the future, severely affected mental health and increased the risk of suicide.^8,9^ Moreover, within a year of the pandemic’s outbreak, hospital admissions and emergency visits for severe mental disorders and suicidal tendencies surged.^10–14^ Although the COVID-19 pandemic is currently under control, similar stressors may arise in the future, highlighting the persistent need to address their effects on mental health.

The prevalence of psychotropic drug use may reflect trends in mental healthcare utilisation or treatment patterns within a population. Studies from Hong Kong^15^ and France^16^ showed that depression rates rose during the pandemic (risk ratio: 1.21, 95% CI 1.10–1.33), with monthly antidepressant sales in France increasing (+0.20 defined daily doses sold per 1000 inhabitants per day). Additionally, studies from Denmark^17^ and France^18^ indicated an increase in psychotropic medication use and mental disorder diagnoses among young people, with variations in prescription trends in different drug types. These findings indicate the growing burden of mental health issues during the pandemic, and highlight the impact of these challenges based on drug types and population characteristics. However, these studies focused on countries with relatively strict lockdown policies, leaving gaps in understanding how more voluntary public health measures, such as those implemented in Japan, influenced mental health outcomes and psychotropic drug use in vulnerable populations.

Japan offers a unique case for examining the mental health impacts of the pandemic because of its relatively less stringent lockdown policies, emphasis on voluntary measures and prolonged school closures. Studies conducted in Europe consistently report increases in psychotropic drug use among young people.^16,18^ However, limited data are available on how Japan’s distinct policy measures and healthcare access dynamics have influenced psychotropic prescriptions for children and adolescents.

This study aimed to utilise data from a large national database to investigate changes in the prescription patterns of psychotropic medications for Japanese individuals aged 6–22 years before and after the COVID-19 pandemic, using the interrupted time-series (ITS) analysis method. Results were further examined by time points, age groups and gender to identify potential differential impacts of the pandemic on these subgroups. Insights from this research may clarify the effects of less stringent isolation policies on vulnerable groups of society during a pandemic.

## Method

### Data source and study population

This nationwide retrospective study used the DeSC Database (DeSC Healthcare Inc, Tokyo, Japan), which includes claims and health check-up data from both National Health Insurance and society-managed health insurance enrollees, covering approximately 4.4 million individuals as of January 2022. The claims data provide anonymised identifiers, demographic details, and medical, dental and pharmaceutical claims. The database covers a wide age range and broad geographic distribution, capturing both employed individuals and their dependents. Although it does not represent the entire Japanese population, it is considered broadly representative of working-age individuals and their families in urban and semi-urban areas. Diagnoses are recorded with ICD-10 codes and Japanese text, and include those made in both hospital and out-patient clinic settings, such as those made by community-based (‘city’) doctors. Dental claims also include standardised dental charts. Medication details encompass names, assessment dates, quantities, drug codes, ingredients and Anatomical Therapeutic Chemical codes from the World Health Organization and the European Pharmaceutical Market Research Association.^19,20^

This study analysed psychiatric medication prescriptions for out-patients aged 6–22 years from February 2016 (4 years pre-COVID-19) to November 2022 (3 years post-pandemic). Participants were divided into children (6–11 years), teenagers (12–17 years) and young adults (18–22 years). Patients were included if they had continuous enrolment data during each relevant analysis period to ensure the availability of complete prescription records. This approach minimised potential bias from data gaps caused by changes in insurance coverage, employment status or mortality. Prescriptions were categorised by drug class: antidepressants (N06A), antipsychotics (N05A), anxiolytics (N05B), hypnotics/sedatives (N05C, excluding melatonin (a hormone primarily used as a chronobiotic to regulate circadian rhythms rather than as a direct sedative) and psychostimulants (N06B), with each category assessed separately for trends and patterns.

The primary focus was on the prescription volume of psychotropic medications, defined as the number of children, adolescents and young adults prescribed these medications, categorised by gender and age group. The underlying assumption posits that the effect of the COVID-19 pandemic on psychiatric medication prescriptions becomes evident only after the pandemic’s onset. Depressive symptoms related to the pandemic are not expected to manifest immediately; rather, new diagnoses and prescriptions for exacerbated symptoms are anticipated to increase as mental health services improve following the emergency declaration.

### Outcome

The number of patients prescribed psychotropic medications in the target demographic were analysed before and after the pandemic’s onset in February 2020, assessing changes in numbers and trends over time.

### Statistical analyses

We initially aggregated individual data at a monthly level to observe the overall prescription trends. We evaluated whether the COVID-19 pandemic directly affected the number of patients prescribed psychotropic medications. Using the first verified COVID-19-related intensive care unit admission in Japan (on 9 February 2020) as a reference, we designated February 2020 as the pandemic’s starting month and divided the data into two periods: pre-COVID-19 pandemic (February 2016 to February 2020) and COVID-19 pandemic (February 2020 to November 2022).

We used ITS analysis^21^ to assess whether the COVID-19 pandemic affected the quantity and/or trends in psychiatric drug use among Japanese children and young adults. We used ordinary least squares regression, accounting for non-stationarity, autocorrelation and seasonality. This study identified the COVID-19 pandemic as its primary ‘intervention’ of interest. The following formula was used:

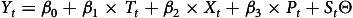

where *Y_t_* represents the number of patients prescribed psychotropic medications over time, *t*. *T_t_* is a continuous variable indicating time elapsed since the beginning of the investigation (in months). The dummy variable *X_t_* distinguishes between the pre-pandemic (coded 0) and pandemic (coded 1) phases. *S_t_* includes seasonal control variables, and *P_t_* is a continuous variable counting the number of months during the pandemic, coded as 0 before and *T_t_* − 51 during the epidemic. *β*_0_ denotes the model’s baseline level, *β*_1_ represents the slope before the pandemic, *β*_2_ reflects immediate changes in prescription patterns after the pandemic onset and *β*_3_ indicates differences in slope before and after the pandemic started.^22,23^ The relationship between the Fourier terms and seasonal trends (*S_t_*) was determined by fitting sine and cosine functions to the time variable. A fundamental period corresponding to the seasonal cycle (calendar months) allowed the regression model to effectively account for and correct predictable seasonal variations. The Durbin–Watson test was used to assess for autocorrelation in the model residuals.^24^ Models showing evidence of autocorrelation were re-estimated by using the Cochrane–Orcutt method until the Durbin–Watson statistic approached 2.^25^ To assess the impact of the COVID-19 pandemic on psychotropic medication prescription patterns, we developed counterfactual scenarios based on pre-pandemic trends. These scenarios assumed no significant disruptions, allowing comparisons between actual changes and expected patterns in the absence of pandemic-related events. Predicted prescription patterns were generated with coefficients from the main model.

Subgroup analyses examined prescription patterns by gender, age (6–11, 12–17 and 18–22 years) and pandemic timeline, with December 2019–January 2020 as cut-offs. Analyses were performed using R statistical software (version 4.4.1 on the Windows platform; R Foundation for Statistical Computing, Vienna, Austria; https://www.r-project.org/), with *P*-values <0.05 considered significant.

### Patient and public involvement

Patients and/or the public were not involved in designing, conducting, reporting or disseminating this study.

## Results

Patients aged 6–22 years were initially selected from the DeSC database, which included a cohort of approximately 4.4 million individuals. The selection was further refined to include those whose data could be continuously tracked between February 2016 and November 2022. This process identified separate populations prescribed psychotropic medications, including antidepressants, antipsychotics, anxiolytics, hypnotics, sedatives and psychostimulants.

Between February 2016 and November 2022, psychotropic medications were prescribed to 93 385 individuals, including those who received antidepressants (*n* = 16 597, 17.78%), antipsychotics (*n* = 38 540, 41.27%), anxiolytics (*n* = 28 750, 30.79%), hypnotics (*n* = 23 790, 25.48%) and sedatives (*n* = 27 448, 29.39%). Young adults (32 425, 34.72%) accounted for the majority of these prescriptions, followed by 27 362 (29.30%) for children and 22 478 (24.07%) for adolescents. In total, 53 092 prescriptions were dispensed to males and 37 173 to females.

### Changes in the prescription pattern of antidepressants

**Fig. 1:**
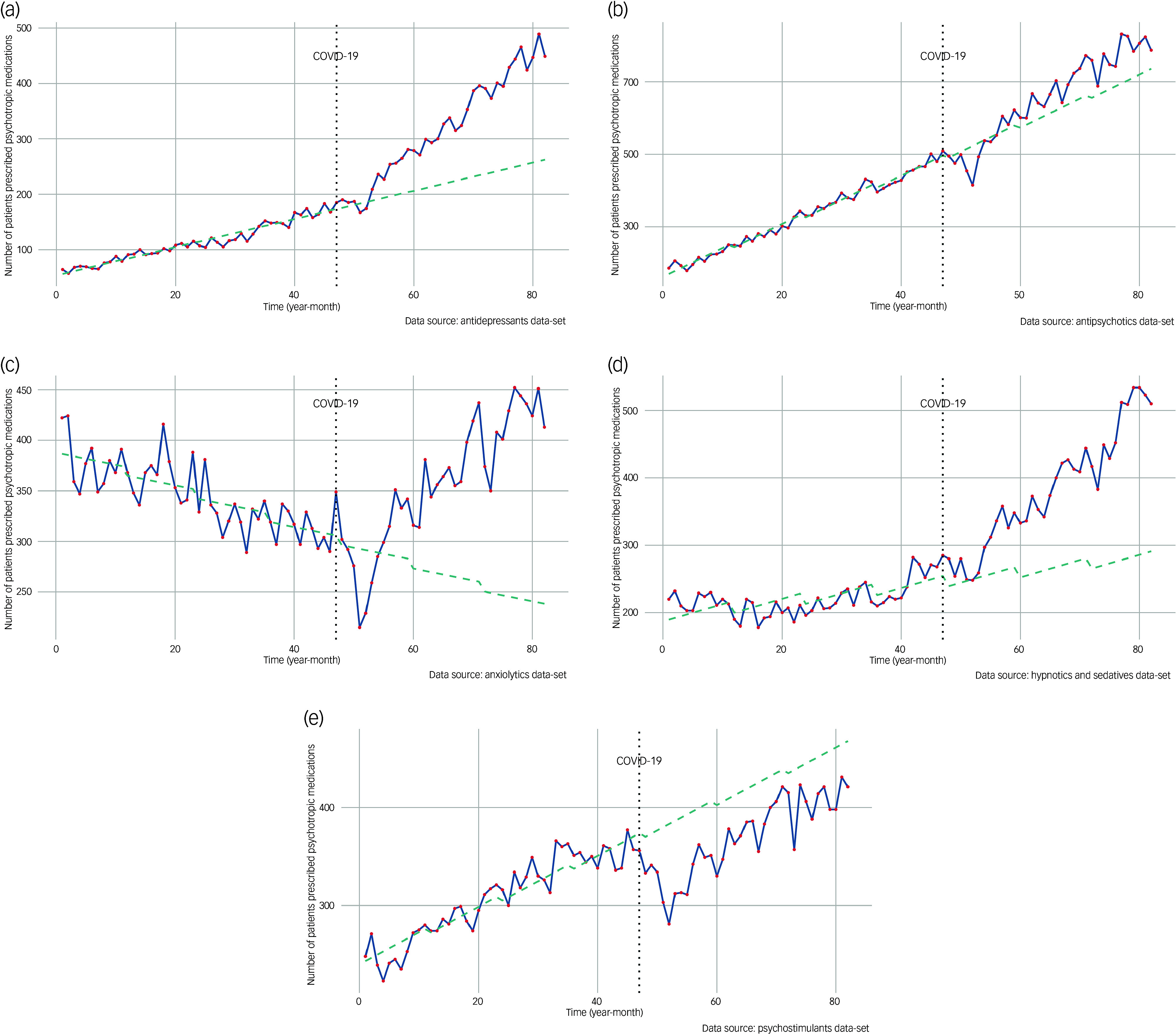
Changes in the estimated number of children, adolescents and young adults in Japan prescribed psychotropic medications. The counterfactual trend lines, derived from pre-pandemic data, were modelled with ordinary least squares with 95% confidence intervals. The onset of the pandemic is marked by vertical dotted lines, set at February 2020. The graphs show changes in the estimated number of children, adolescents and young adults in Japan prescribed (a) antidepressants, (b) antipsychotics, (c) anxiolytics, (d) hypnotics and sedatives and (e) psychostimulants.

In February 2016, antidepressants were prescribed to 64 children, adolescents and young adults. Before the pandemic, this figure was increasing at a rate of 2.5 patients per month (95% CI 2.2–2.8) (Table 1, Fig. 1). The number of patients prescribed antidepressants did not substantially change during the early stages of the epidemic and after its onset (estimates −1.2, *P* = 0.84). However, the trend significantly shifted, with an increase of 6.5 patients per month (95% CI 5.9–7.1), ultimately reaching 449 by November 2022.

**Table 1:** Changes in the levels and trends of the number of patients prescribed psychotropic medications before and after the outbreak of the COVID-19 pandemic

	Before the pandemic onset		Initial pandemic period		After the pandemic onset	
Type of medication	Level	Trend	Change in level	*P*-value	Trend	*P*-value
Antidepressants	58.5 (45.5–71.4)	2.5 (2.2–2.8)	−1.2 (−13.8 to 11.3)	0.84	6.5 (5.9–7.1)	<0.001
Antipsychotics	167.4 (143.1–191.8)	6.9 (6.3–7.5)	−25.3 (−49.5 to −1.0)	0.04	4.1 (3.0–5.3)	<0.001
Anxiolytics	379.0 (352.0–406.1)	−1.8 (−2.5 to −1.1)	−30.2 (−57.4 to −3.0)	0.03	7.4 (6.0–8.7)	<0.001
Hypnotics and sedatives	172.1 (145.0–199.0)	1.5 (0.7–2.2)	−4.7 (−30.3 to 20.9)	0.71	6.6 (5.2–8.0)	<0.001
Psychostimulants	242.3 (222.8–261.7)	2.6 (2.1–3.1)	−50.3 (−69.8 to −30.8)	<0.001	0.60 (−0.34 to 1.54)	0.21

The table shows the initial number of patients (level), trends in the rate of change, and shifts in both levels and trends across different time periods in estimates and 95% confidence intervals.

### Changes in the prescription pattern of antipsychotics

In February 2016, antipsychotics were prescribed to 186 children, adolescents and young adults. Pre-pandemic, the number of patients prescribed antipsychotics increased by 6.9 per month (95% CI 6.3–7.5) (Table 1, Fig. 1). This figure significantly decreased during the early stages of the pandemic and the period following its onset (estimates −25.3, *P* = 0.04). Despite this initial decline, the overall trend did not show a significant increase, with prescriptions increasing by 4.1 per month (95% CI 3.0–5.3) and totalling 787 by November 2022.

### Changes in the prescription pattern of anxiolytics

In February 2016, 422 children, adolescents and young adults were prescribed anxiolytics, with a pre-pandemic monthly decline of −1.8 (95% CI −2.5 to −1.1) (Table 1, Fig. 1). During the initial pandemic period and shortly afterward (estimates −30.2, *P* = 0.03), the number of patients prescribed anxiolytics significantly decreased. However, the trend later reversed, with prescriptions increasing by 7.4 patients per month (95% CI 6.1–8.7), reaching a total of 413 by November 2022.

### Changes in the prescription pattern of hypnotics and sedatives

In February 2016, 220 children, adolescents and young adults were prescribed hypnotics and sedatives, showing a pre-pandemic monthly increase of 1.5 (95% CI 0.7–2.2) (Table 1, Fig. 1). The number of patients prescribed hypnotics and sedatives did not considerably decrease during the early pandemic period or after its onset (estimates −4.7, *P* = 0.71). However, a significant change was found in the trend, with the number of patients prescribed these medications increasing by 6.6 per month (95% CI 5.2–8.0), reaching a total of 510 by November 2022.

### Changes in the prescription pattern of psychostimulants

In February 2016, 248 children, adolescents and young adults were prescribed psychostimulants, with a pre-pandemic monthly increase of 2.6 (95% CI 2.1–3.1) (Table 1, Fig. 1). This trend significantly reversed during the initial pandemic period, with a substantial decrease in prescriptions that persisted after the onset of COVID-19 (estimates −50.3, *P* < 0.001). Although the number of patients prescribed psychostimulants began to rise again by 0.6 per month (95% CI −0.3 to 1.5), the trend did not exhibit a statistically significant rebound, ultimately reaching a total of 421 by November 2022.

### Subgroup analyses

Two subgroup analyses, stratified by age and gender, revealed statistically significant changes in the number of patients, particularly among adolescents and females (Tables 2 and 3). Among adolescent patients, the trend in antidepressant prescriptions significantly increased from 0.40 (95% CI 0.25–0.53) before the pandemic to 3.2 (95% CI 2.9–3.5) afterward. Antipsychotic prescriptions decreased from 2.8 (95% CI 2.6–3.0) to 1.7 (95% CI 1.3–2.1). Anxiolytic prescriptions initially showed a negative trend of −0.68 (95% CI −0.94 to −0.43), but later surged to 3.2 (95% CI 2.6–3.9). Trends in hypnotic and sedative prescriptions increased from 0.36 (95% CI 0.15–0.57) to 3.3 (95% CI 2.9–3.7). However, psychostimulant prescriptions initially showed a positive trend of 2.0 (95% CI 1.8–2.3), followed by a notable decline to −1.5 (95% CI −1.9 to −1.0).

**Table 2:** Subgroup analysis of changes in the levels and trends of patients prescribed anxiolytics, hypnotics/sedatives and psychostimulants across different age groups (children, adolescents and young adults) before and after the outbreak of the COVID-19 pandemic

	Before the pandemic onset		Initial pandemic period		After the pandemic onset	
Number of patients	Level	Trend	Change in level	*P*-value	Trend	*P*-value
Antidepressants						
Children	2.5 (0.6–4.4)	0.08 (0.03–0.14)	6.1 (4.3–7.9)	<0.001	0.18 (0.09–0.27)	<0.001
Adolescents	25.3 (18.5–32.1)	0.40 (0.25–0.54)	−4.8 (−10.9 to 1.2)	0.11	3.2 (2.9–3.5)	<0.001
Young adults	33.7 (25.8–41.6)	2.0 (1.8–2.1)	−2.2 (−9.3 to 4.7)	0.52	3.2 (2.9–3.5)	<0.001
Antipsychotics						
Children	2.1 (−3.8 to 8.0)	1.1 (1.0–1.2)	−2.3 (−7.7 to 3.0)	0.38	1.5 (1.3–1.7)	<0.001
Adolescents	61.7 (51.2–72.2)	2.8 (2.6–3.0)	−9.6 (−19.0 to −2.0)	0.04	1.7 (1.3–2.1)	<0.001
Young adults	107.8 (96.0–119.7)	2.9 (2.6–3.1)	−15.7 (−26.3 to −5.1)	<0.01	1.2 (0.8–1.7)	<0.001
Anxiolytics						
Children	201.0 (187.4–214.6)	−2.4 (−2.7 to −2.2)	−31.4 (−43.6 to −19.3)	<0.001	2.3 (1.7–2.8)	<0.001
Adolescents	94.7 (82.6–106.9)	−0.68 (−0.94 to −0.43)	−0.9 (−11.8 to 9.9)	0.86	3.4 (2.9–3.9)	<0.001
Young adults	87.7 (73.5–101.9)	1.2 (0.9–1.5)	−1.5 (−14.2 to 11.2)	0.81	2.1 (1.5–2.6)	<0.001
Hypnotics and sedatives						
Children	201.0 (187.4–214.6)	−2.4 (−2.7 to −2.2)	−31.4 (−43.6 to −19.3)	<0.001	2.3 (1.7–2.8)	<0.001
Adolescents	41.7 (31.6–51.8)	0.3 (0.1–0.6)	6.3 (−2.6 to 15.3)	0.16	3.3 (2.9–3.7)	<0.001
Young adults	46.7 (36.8–56.5)	2.0 (1.8–2.1)	−10.3 (−19.1 to −1.4)	0.02	2.5 (2.1–2.9)	<0.001
Psychostimulants						
Children	−6.21 (−12.35 to −0.07)	1.1 (0.9–1.2)	−2.5 (−8.0 to 3.0)	0.37	1.7 (1.4–2.0)	<0.001
Adolescents	139.9 (128.3–151.5)	2.0 (1.7–2.3)	−41.8 (−52.3 to −31.4)	<0.001	−1.5 (−1.9 to −1.0)	<0.001
Young adults	106.4 (99.6–113.3)	−0.33 (−0.47 to −0.18)	−19.5 (−25.6 to −13.4)	<0.001	0.58 (0.31–0.86)	<0.001

The table shows the initial number of patients (levels), trends in prescription rates and changes in both levels and trends across different time periods in estimates and 95% confidence intervals.

**Table 3:** Gender-based subgroup analysis of changes in the levels and trends of patients prescribed anxiolytics, hypnotics/sedatives and psychostimulants before and after the outbreak of the COVID-19 pandemic

	Before the pandemic onset		Initial pandemic period		After the pandemic onset	
Number of patients	Level	Trend	Change in level	*P*-value	Trend	*P*-value
Antidepressants						
Male	20.7 (15.4–26.0)	1.2 (1.1–1.3)	−6.5 (−11.3 to −1.8)	<0.01	2.2 (2.0–2.4)	<0.001
Female	33.1 (25.2–41.1)	1.1 (0.9–1.2)	8.4 (1.3–15.5)	0.02	3.6 (3.2–3.9)	<0.001
Antipsychotics						
Male	70.7 (61.5–79.7)	2.5 (2.3–2.7)	−5.1 (−13.3 to 2.9)	0.21	1.4 (1.1–1.8)	<0.001
Female	87.0 (72.7–101.3)	3.8 (3.5–4.1)	−23.7 (−36.5 to −10.9)	<0.001	2.3 (1.6–2.8)	<0.001
Anxiolytics						
Male	148.4 (139.0–157.8)	−0.63 (−0.83 to −0.44)	−20.7 (−29.1 to −12.2)	<0.001	2.6 (2.3–3.0)	<0.001
Female	197.9 (182.8–212.9)	−0.96 (−1.28 to −0.65)	−12.2 (−25.7 to –1.2)	0.07	4.1 (3.5–4.7)	<0.001
Hypnotics and sedatives						
Male	84.1 (72.9–95.2)	0.23 (0.002–0.46)	4.5 (−5.4 to 14.5)	0.36	2.8 (2.4–3.3)	<0.001
Female	84.3 (72.4–96.2)	0.80 (0.56–1.05)	0.6 (−10.0 to 11.2)	0.91	3.2 (2.7–3.7)	<0.001
Psychostimulants						
Male	99.6 (92.2–107.9)	1.1 (0.9–1.3)	−23.5 (−31.0 to −16.0)	<0.001	0.47 (0.13–0.80)	0.006
Female	119.3 (108.5–130.1)	1.5 (1.3–1.7)	−34.9 (−44.5 to −25.2)	<0.001	0.15 (−0.27 to 0.58)	0.47

The table displays the initial number of patients (levels), trends in prescription rates and changes in both levels and trends for male and female patients across different time periods in estimates and 95% confidence intervals.

Female patients experienced a rise in antidepressant prescription trends, which increased from 1.1 (95% CI 0.9–1.2) pre-pandemic to 3.6 (95% CI 3.2–3.9) post-pandemic. Antipsychotic prescriptions decreased from 3.8 (95% CI 3.5–4.1) to 2.3 (95% CI 1.7–2.8), and anxiolytic prescriptions, despite an initial downward trend of −0.9 (95% CI −1.2 to −0.6), sharply increased to 4.1 (95% CI 3.5–4.7). Hypnotic and sedative prescriptions also significantly rose, from 0.8 (95% CI 0.6–1.1) to 3.2 (95% CI 2.7–3.7). Meanwhile, psychostimulant prescriptions showed greater stability, shifting from 1.5 (95% CI 1.3–1.7) to 0.1 (95% CI −0.2 to 0.6).

## Discussion

In this study, we examined the impact of the COVID-19 pandemic on psychotropic medication prescriptions for Japanese children and adolescents, by drug type. Results showed significant increases in prescriptions for antidepressants, anxiolytics and hypnotics/sedatives following the pandemic. By contrast, prescription trends for antipsychotics and psychostimulants remained relatively unchanged. Notably, a significant increase in psychotropic prescriptions was observed among females and those aged 12–17 years.

Japan addressed the COVID-19 pandemic through nationwide school closures and individual behavioural changes, avoiding the strict lockdowns implemented by other countries.^26^ During the pandemic and its associated response, a short-term increase in suicide rates among young people in Japan was reported.^27^ However, the long-term effects of mental stress on psychotropic medication use, as reported in other countries,^16,18,28^ have been overlooked. The results of the present study indicate that even in countries with less stringent lockdown measures, such as Japan, the mental stress experienced by schoolchildren was severe enough to require psychiatric consultations and prescriptions. This highlights the need to provide adequate mental healthcare for schoolchildren during a pandemic.

Our findings are consistent with those of previous studies and indicate a link between the COVID-19 pandemic and changes in psychotropic medication use.^28–30^ The quasi-experimental methodology employed in this study provides stronger evidence of causal relationships. Existing studies consistently demonstrate that the pandemic has adversely affected the mental health of children and adolescents, leading to increased anxiety and depression and a rise in the use of psychotropic medications.^8,14,17^ In our study, we observed a temporary decline in prescriptions across five categories of psychotropic medications following the COVID-19 outbreak, followed by a sustained increase. The initial decrease may reflect reduced access to healthcare during periods of strict lockdowns^31,32^ and social isolation.^33^

An important finding of this study is the significant increase in prescriptions for antidepressants, anxiolytics and hypnotics/sedatives during the pandemic. These trends are consistent with similar studies, including those conducted in France, which reported comparable patterns.^18^ Recent reports have shown an increase in mood and anxiety disorders among children and adolescents,^28,29,30,34^ which aligns with our findings, as these conditions are commonly treated with medications from these psychotropic categories. Notably, anxiolytic prescriptions, which had been steadily declining before the pandemic, showed a sharp reversal and a significant increase after its onset. This surge suggests that the pandemic either introduced or exacerbated anxiety-related symptoms.

By contrast, prescriptions for antidepressants and hypnotics/sedatives were already increasing before the pandemic, but this growth significantly accelerated during the COVID-19 crisis. The pandemic seems to have aggravated pre-existing conditions such as depression and sleep disturbances,^35^ emphasising the need for long-term management strategies. Prescribing specific psychotropic medications is a common first-line treatment for paediatric populations, a practice consistent with findings from previous studies in other countries.^18,28,30,36^ The consistency of these trends across international research highlights the need of developing standardised approaches to managing mental health conditions in children and adolescents.

Trends for antipsychotic and psychostimulant prescriptions did not exhibit a significant increase, suggesting that conditions such as psychosis and attention-deficit hyperactivity disorder may be less affected by pandemic-related stressors compared with mood and anxiety disorders. Notably, psychostimulant prescriptions declined at the beginning of the pandemic, representing the largest decrease among all psychiatric medications. This initial decline highlights concerns about disruptions in healthcare access during the pandemic, and emphasises the necessity of adopting more flexible policies for psychostimulant prescriptions during such crises. However, the relatively stable prescribing patterns for attention-deficit hyperactivity disorder and psychosis should also be interpreted with caution, as they may reflect factors such as reduced help-seeking, limited care accessibility or smaller sample sizes, rather than lack of pandemic-related impact on these conditions.

Females and individuals aged 12–17 years were disproportionately affected by the pandemic, as reflected in greater increases in psychotropic prescriptions. Adolescent females may be more vulnerable to pandemic-related stressors, including social pressures and a heightened risk of suicide, which are particularly pronounced in Japan.^32,37,38^ This gender disparity aligns with broader patterns, as females generally exhibit greater susceptibility to anxiety and depression during adolescence, caused by a complex interplay of biological, psychological and sociocultural factors. The greater increase in psychotropic prescriptions among females may not only reflect increased mental health needs, but also potential gender differences in clinical recognition and prescription practices. Girls may be more likely to seek help and be diagnosed with internalising disorders. Additionally, reduced access to non-pharmacological support, such as therapy or school-based services during the pandemic, may have led to increased reliance on medications.

The rise in psychotropic prescriptions during the pandemic highlights the need for targeted mental healthcare, especially for vulnerable adolescents and females. Clinicians should focus on early identification, integrated treatments combining pharmacotherapy and psychotherapy, and regular monitoring to optimise efficacy, manage side-effects and consider deprescription when appropriate.

### Strengths and limitations

Our study had several strengths. First, we used an epidemiological database that provides a robust foundation for extrapolating nationwide data. The DeSC database, which represents Japan’s national healthcare information, is particularly valuable for examining the effects of the COVID-19 pandemic on psychotropic prescriptions, a topic that has been relatively under-researched in Japan.^19^ Second, in contrast to previous studies from other countries, we conducted age-stratified analyses and examined the data by gender. Previous research indicated the influence of gender on psychotropic drug use during the pandemic. Furthermore, we selected the number of patients as our primary outcome variable rather than relying solely on the number of prescriptions or defined daily doses. This approach minimises the risk of double counting and inconsistencies in dosing, which are common pitfalls when using prescription data alone. In addition, although changes in psychotropic prescribing patterns may reflect underlying trends in mental health burden, particularly during the pandemic, they do not necessarily indicate the actual incidence or prevalence of mental disorders, especially among children and adolescents, who may not access clinical services or receive pharmacological treatment as a first-line option.

Several limitations should be acknowledged. Various deprivation indicators, such as household structure (e.g. single-parent households), could influence medication use. However, the lack of relevant data in the database prevented us from incorporating these factors into the analysis. Additionally, the DeSC database includes a higher proportion of individuals covered by non-employee insurance, a group that typically has lower incomes than those insured through employee insurance.

The ITS study showed significant upward trends in the number of Japanese children, adolescents and young adults prescribed antidepressants, anxiolytics and hypnotics/sedatives during the COVID-19 pandemic. The pronounced changes observed in individuals aged 12–17 years and females suggest that these groups may have been profoundly affected. These findings have long-term implications for youth mental health in Japan, where lockdown measures were relatively less stringent. They highlight the need for ongoing monitoring and preparedness to address potential increases in demand for mental health services.

## Supporting information

Huang et al. supplementary material Huang et al. supplementary material

## Data Availability

The data-sets used in this study contain individual-level sensitive information from the national register data. According to data protection legislation and the safety restrictions of the Department of Clinical Epidemiology and Health Economics, School of Public Health, Graduate School of Medicine, The University of Tokyo, the authors are not allowed to share these sensitive data directly upon request. The SQL code used for data-set cleaning contains sensitive individual information and is therefore not publicly available. However, the codes used for the statistical models referenced in the main text are provided in the Supplementary Appendix. This appendix includes the R code for the interrupted time series analysis and graphing, and a documentation file describing the variables in the original data-sets. All codes are available from the corresponding author, W.H., upon request.

## Supplementary material

The supplementary material is available online at https://doi.org/10.1192/bjo.2025.10903
