# Supplementary material for: Changes in psychotropic medication prescription patterns during the COVID-19 pandemic among Japanese children, adolescents and young adults: interrupted time-series study using a national claims database
Source: BJPsych Open. 2025 Nov 14;11(6):e281. doi: 10.1192/bjo.2025.10903 (PMC12641412; doi:10.1192/bjo.2025.10903)
Supplement: Huang et al. supplementary material [file S2056472425109034sup001.pdf]

Supplementary Appendix

Changes in Psychotropic Medication Prescription Patterns During the COVID-19 Pandemic Among Japanese Children, Adolescents, and Young Adults: An Interrupted Time Series Study Using a National Claims Database

TOKYO, JAPAN, MAY 01 2024

Research Protocol

Changes in Psychotropic Medication Prescription Patterns During the COVID-19 Pandemic Among Japanese Children, Adolescents, and Young Adults: An Interrupted Time Series Study Using a National Claims Database

(This study protocol has been reviewed and preregistered with DeSC Healthcare, Inc.; DeSC Administration Number: RWD29)

Wenbo Huang[1], Hiroki Matsui[1], Hideo Yasunaga[1]

1 Department of Clinical Epidemiology and Health Economics, School of Public Health, Graduate School of Medicine, The University of Tokyo, Tokyo, Japan.

##################################

Wenbo Huang

Department of Clinical Epidemiology and Health Economics, School of Public Health, Graduate School of Medicine, The University of Tokyo, 7-3-1 Hongo, Bunkyo-ku, Tokyo 113033, Japan

E-mail: wenbohuang@g.ecc.u-tokyo.ac.jp

Telephone number: +81 08061091154

#################################

Mental health disorders and psychological issues are among the most serious challenges in pediatric healthcare. Currently, nearly 15% of kids and adolescents get diagnosed with a mental condition by the age of 18 (1). In Japan, social isolation and suicidal behavior among youth due to mental health disorders have always been major concerns (2, 3). Recent data from the National Police Agency show that issues such as "futoko" (school refusal) have significantly increased since 2023. Additionally, the number of bullying cases involving students that led to police investigations reached 292 in 2023, a sharp rise from the previous year and the highest level in a decade (4). Japan's unique social atmosphere and behavioral norms may also contribute to this increase.

The global spread of COVID-19 has significantly exacerbated mental health issues by introducing various stressors such as social isolation and economic uncertainty (5-8). These challenges and anxieties about the future can severely impact mental health and increase the risk of suicide (9, 10). Moreover, there has been a significant increase in hospitalizations and emergency visits for severe mood disorders and suicidal tendencies within a year after the pandemic outbreak (11-15). Although the COVID-19 pandemic is now relatively under control, similar issues could arise in the future, making the impact of these stressors on mental health a persistent concern. If such issues re-emerge, urgent public health measures will be needed to address the underlying problems. However, non-pharmacological mental health services for children and young people remain insufficiently available worldwide.

The use of psychotropic drugs is typically regarded indicative of the overall mental health condition or the prevalence of specific mental disorders within a population. Studies from Hong Kong (16) and France (17) have shown that during the COVID-19 pandemic, the incidence of depression increased among individuals with depression (risk ratio = 1.21, 95% CI: 1.10–1.33), and there was a rise in monthly antidepressant sales in France (+0.20 defined daily doses sold per 1000 inhabitants per day). These findings suggest that the existing mental health services were insufficient

to meet the growing demand for depression care, highlighting a significant gap in the provision of mental health services. Additionally, studies from Denmark (18) and France (19) indicated an increase in psychotropic medication use and mental disorder diagnoses among young people, with varying trends in different drug prescriptions. Importantly, a previous interrupted time series (ITS) analysis (20) indicated that the COVID-19 pandemic negatively impacted suicide rates in Japan, with the most pronounced effects seen among women and younger demographics. Although there was an initial decrease in suicide rates during the early stages of the pandemic, potentially due to increased community bonding and mutual assistance, the long-term effects on mental health remain unclear. Specifically, it is uncertain whether the pandemic has led to changes in psychotropic medication prescription patterns among Japanese children, adolescents, and young adults compared to pre-pandemic times. This is important because prescription patterns can serve as an indicator of broader trends in mental health and access to treatment. Additionally, reports suggest that the long-term mental health impacts of the pandemic are limited. Furthermore, it remains unclear whether strict isolation policies, such as the COVID-19 emergency declaration (緊急事態宣言; Kinkyujitaisengen), are associated with changes in psychotropic medication use and prescription patterns. Investigating these patterns could provide insights into the indirect effects of the pandemic on mental health care and help identify potential gaps in service provision.

This study aims to utilize a large national database to investigate changes in the prescription patterns of psychotropic medications for Japanese individuals under 22 before and after the COVID-19 pandemic, using the ITS analysis method. Additionally, the study will assess the impact of government policies, such as the COVID-19 emergency declaration. When the government implements intervention measures at specific points, sharp changes may occur in the ongoing time series data related to COVID-19, making ITS analysis a suitable statistical method for evaluating the effects of these interventions. We will further analyze the results by time point, age, and gender groups to identify any differential impacts of the pandemic on these specific subgroups. The findings will contribute to the overall health and welfare of society in Japan and may provide insights for responses in other countries and future pandemics.

Methods

Data and study population

This study examines all prescriptions for psychiatric medications written for outpatients between the ages of 6 and 22 from February 9, 2016 (approximately four years before the onset of COVID-19), to February 8, 2023 (approximately three years after the pandemic began). The age groups are categorized as follows: children (6 to 11 years old), adolescents (12 to 17 years old), and young adults (18 to 22 years old). We included only patients with continuous data reported throughout this period. This criterion is crucial due to potential limitations in the database, such as missing data related to COVID-19 deaths. Additionally, in Japan, health insurance primarily falls under two types: kokuho (the National Health Insurance system) and kenpo (the Employees' Health Insurance system). When individuals retire or leave their employment positions, there may be instances where their data becomes unavailable. The prescriptions analyzed encompass all outpatient records containing at least one psychotropic medication, including antipsychotics (ATC code identified from database: N05A, N05AA01, N05AA02, N05AB02, N05AB03, N05AB04, N05AC01, N05AD, N05AD01, N05AD05, N05AD06, N05AD08, N05AE01, N05AE05, N05AG02, N05AH02, N05AH03, N05AH04, N05AH05, N05AL, N05AL01, N05AL02, N05AL03, N05AN01, N05AX, N05AX08, N05AX10, N05AX11, N05AX12, N05AX13, N05AX16), anxiolytics (N05BA, N05BA01, N05BA02, N05BA03, N05BA05, N05BA06, N05BA08, N05BA09, N05BA12, N05BA17, N05BA18, N05BA19, N05BA21, N05BA22, N05BA23, N05BA25, N05BB01, N05BE), hypnotics and sedatives (N05CA01, N05CA02, N05CA04, N05CA06, N05CC01, N05CD, N05CD01, N05CD02, N05CD03, N05CD04, N05CD05, N05CD06, N05CD08, N05CD09, N05CD10, N05CD14, N05CD15, N05CF01, N05CF02, N05CF04, N05CH02, N05CM03, N05CM05, N05CM07, N05CM09, N05CM11, N05CM18, N05CM19, N05CM21, except N05CH01: melatonin), antidepressants (N06AA02,

N06AA04, N06AA06, N06AA07, N06AA09, N06AA10, N06AA16, N06AA17, N06AA21, N06AB05, N06AB06, N06AB08, N06AB10, N06AX, N06AX03, N06AX05, N06AX11, N06AX16, N06AX17, N06AX21, N06AX26), and psychostimulants (N06BA04, N06BA05, N06BA07, N06BA09, N06BA12, N06BC01, N06BX01, N06BX03, N06BX06).

Outcome

The monthly number of psychotropic medication prescriptions among children, adolescents, and young adults were analyzed before and after the onset of the pandemic in February 2020, with changes in rates and trends being assessed.

Statistical analyses

This nationwide retrospective cohort study utilizes the DeSC Database (DeSC Healthcare, Inc; Tokyo, Japan), which contains claims information and specific health check-up details provided by various health insurance societies, including national health insurance and late-elderly healthcare (wide-area union for elderly healthcare). As of January 2022, the database encompasses approximately 4.4 million individuals. The primary focus is on the monthly number of psychotropic medication prescriptions among children, adolescents, and young adults, categorized by gender and age group. The hypothesis is that the effect of the COVID-19 epidemic on prescriptions for psychiatric medications will become evident post-pandemic onset. It is assumed that related depressive symptoms do not manifest immediately and that new diagnoses and prescriptions for exacerbated symptoms will increase only if mental health services improve following the emergency declaration. As the first verified COVID-19-related ICU admission in Japan

occurred on February 9, 2020, that date is used in this study as the breakpoint, separating the data into two periods: pre-COVID-19 pandemic (February 9, 2016, to February 8, 2020) and COVID-19 pandemic (February 9, 2020, to February 8, 2023).

We use ITS analysis (21) to investigate whether the COVID-19 pandemic and the COVID-19 emergency declaration affect the number and/or trends in the use of psychotropic medications among children and young people in Japan. The COVID-19 pandemic is the "intervention" of interest in this study. We use the following formula:

$$Yt = \beta 0 + \beta 1 \times Tt + \beta 2 \times Xt + \beta 3 \times Pt + St$$

where Yt is the number of psychotropic medications over time t; Tt is a continuous variable representing time from the start of the study (in months); Pre-pandemic (coded 0) or pandemic phase (coded 1) is indicated by the dummy variable Xt; St stands for seasonal control variables, and Pt is a continuous variable that counts the number of months throughout the pandemic, coded 0 before and (Tt - 51) during the epidemic. In this model, $\beta 0$ represents the baseline level; $\beta 1$ is the slope before the pandemic; $\beta 2$ shows whether there is an immediate change in prescription patterns after the pandemic; and $\beta 3$ is the difference in slope before and after the pandemic onset (22, 23). Fitting Fourier terms with sine and cosine functions of time with a fundamental period matching the full seasonal cycle (calendar months) allows for the adjustment of seasonal patterns in the regression model. This method works well for capturing predictable seasonal patterns. Additionally, to compare the actual COVID-19 pandemic's impact on psychotropic medication prescription patterns with a hypothetical scenario without any mitigation measures, we develop counterfactual scenarios. Predicted prescription patterns are calculated using the coefficients from the main model (producing Newey-West standard errors of the coefficients estimated by ordinary least squares regression).

First, we conduct a descriptive analysis of psychotropic medication data. For continuous variables, the standard deviation (SD) and median are shown, along with the interquartile range (IQR). For categorical variables, frequencies and percentages are displayed. Subsequently, we use ITS analysis to evaluate changes in psychotropic medication sales following the onset of the COVID-19 pandemic. To address autocorrelation, which can lead to underestimated standard errors, we employ six statistical models: ordinary least squares regression (OLS), OLS with Newey-West standard errors, Prais-Winsten, restricted maximum likelihood (REML) (with and without the small sample Satterthwaite approximation), and autoregressive integrated moving average (ARIMA). These models estimate monthly changes in drug prescriptions before and after the pandemic's onset. Given that the first, second, and third COVID-19 emergency declarations significantly reduced daily infection case counts in Tokyo and the surrounding prefectures, we will also examine the impact of such measures on prescription patterns.

Additionally, we conduct sensitivity analyses with different model specifications to account for non-stationarity, autocorrelation, and seasonality, examining differences in prescription patterns by time point, gender, and age. Initially, we confirm changes in prescription patterns 1, 2, 3, and 6 months after the pandemic onset. Next, we perform gender-specific analyses to identify the impact of gender differences. Furthermore, data from young adults (18–22 years old), adolescents (12–17 years old), and children (6–11 years old) are analyzed to assess if results differ with varying age. Finally, considering the timing of the pandemic, we redefine December 2019 and January 2020 as cutoff points for ITS analysis to explore changes in prescription patterns. The analysis is conducted using Stata statistical software (version 16; Stata Corp, College Station, TX, USA) and R statistical software (version 4.0.5). The statistical significance level is set at $p < 0.05$, and all p-values are two-sided.

Results

These findings indicate a significant and persistent increase in the prescription of psychotropic medications for children, adolescents, and young adults in Japan following the onset of the COVID-19 pandemic. Moreover, the declaration of the COVID-19 emergency significantly contributed to the rise in psychotropic medication prescriptions. Our study offers several notable meanings. Firstly, we utilized a national hospital pharmacy database, facilitating the extrapolation of findings to a national level. Additionally, considering the escalating rates of pediatric psychotropic medication prescriptions in Japan in recent years, our research employs time series statistical analysis that accounts for these pre-pandemic trends. It is imperative for Japanese society and clinical experts to recognize this trend and implement appropriate measures accordingly.

Data availability

The datasets used in this study contain individual-level sensitive information from national register data. According to data protection legislation and the safety restrictions of Department of Clinical Epidemiology and Health Economics, School of Public Health, Graduate School of Medicine, The University of Tokyo (clinepi_contact@www.heer.m.u-tokyo.ac.jp), the authors are not allowed to share these sensitive data directly upon request. However, the data are available for research upon request to DeSC Healthcare (https://desc-hc.co.jp/en/contact). Requests must include details such as affiliation, name, contact information, research purpose, results sharing plan, and ethical considerations. Access is granted in accordance with the DeSC Healthcare Privacy Policy.

Code availability

The SQL code used for dataset cleaning contains sensitive individual information and is therefore not publicly available. However, the codes used for the statistical models referenced in the main text are provided in the Supplementary Appendix. This appendix includes Stata code for ITSA analysis, R code for ITSA analysis and graphing, and a documentation file describing the variables in the original datasets. All codes are available from the corresponding author, Mr. Wenbo Huang (19435061@life.hkbu.edu.hk or wenbohuang@g.ecc.u-tokyo.ac.jp), upon request.

Author contributions

W.H., H.M. and H.Y. designed the study. W.H., and H.M. analyzed the data. W.H. drafted the paper. W.H., H.M. and H.Y. contributed to the critical interpretation of the results and development of the report. W.H., was corresponding author and took the responsibility for the study. All authors have seen and approved the final version. All authors have read, and confirmed that they meet, ICMJE criteria for authorship.

Competing interests

The authors declare no competing interests.

Acknowledgements

We utilized *Consensus*, an AI Search Engine for Research, in our literature searching process (https://consensus.app/).

We also want to express our gratitude to Dr. Jiazheng Tan of the School of Engineering, The University of Edinburgh, for his invaluable assistance with academic advice.

Funding

This work was supported by a grant from the Ministry of Health, Labour and Welfare, Japan (23AA2003). This study also received funding from the "Fostering Advanced Human Resources to Lead Green Transformation (GX)" project, a program for Support for Pioneering Research Initiated by Next Generation (SPRING) of Japan Science and Technology Agency (JST), which supported various aspects of the research process, including equipment procurement and personnel costs. It is important to note that while the Ministry of Health, Labour and Welfare and JST SPRING provided financial support, the funders had no role in study design, data collection and analysis, decision to publish, or preparation of the manuscript. The authors retained full independence in conducting the analysis, interpreting the results, and drafting the manuscript.

Ethics

The DescQ_202312 Database was established by the DeSC Healthcare Inc (https://desc-hc.co.jp/en), is an epidemiological receipt database that has accumulated receipts (inpatient, outpatient, dispensing) and medical examination data received from multiple health insurance associations since 2005. This study was approved by the Institutional Review Board of the Graduate School of Medicine at the University of Tokyo (approval number: 2021010NI [23 April 2021]). The requirement for written consent was waived owing to data anonymity.

References

1. Polanczyk GV, Salum GA, Sugaya LS, Caye A, Rohde LA. Annual research review: A meta-analysis of the worldwide prevalence of mental disorders in children and adolescents. J Child Psychol Psychiatry. 2015;56(3):345-65.

2. Endo K, Ando S, Shimodera S, Yamasaki S, Usami S, Okazaki Y, et al. Preference for Solitude, Social Isolation, Suicidal Ideation, and Self-Harm in Adolescents. J Adolesc Health. 2017;61(2):187-91.

3. Matsubayashi T, Ueda M, Yoshikawa K. School and seasonality in youth suicide: evidence from Japan. J Epidemiol Community Health. 2016;70(11):1122-7.

4. Daichi Itakura KK, Yukihito Takahama and senior staff writer Mayumi Ujioka. Police tackle record 292 cases of bullying at schools in 2023. The Asahi Shimbun. April 8, 2024

5. Nicola M, Alsafi Z, Sohrabi C, Kerwan A, Al-Jabir A, Iosifidis C, et al. The socio-economic implications of the coronavirus pandemic (COVID-19): A review. Int J Surg. 2020;78:185-93.

6. Wang Y, Su B, Xie J, Garcia-Rizo C, Prieto-Alhambra D. Long-term risk of psychiatric disorder and psychotropic prescription after SARS-CoV-2 infection among UK general population. Nature Human Behaviour. 2024.

7. Creswell KG, Hisler GC, Lyons G, Carrillo-Álvarez FA, Fairbairn CE, Wright AGC. Changes in alcohol consumption and alcohol problems before and after the COVID-19 pandemic: a prospective study in heavy drinking young adults. Nature Mental Health. 2024.

8. Ma J, Hua T, Zeng K, Zhong B, Wang G, Liu X. Influence of social isolation caused by coronavirus disease 2019 (COVID-19) on the psychological characteristics of hospitalized schizophrenia patients: a case-control study. Transl Psychiatry. 2020;10(1):411.

9. Nakao T, Murayama K, Takahashi S, Kayama M, Nishi D, Horinouchi T, et al. Mental Health Difficulties and Countermeasures during the Coronavirus Disease Pandemic in Japan: A Nationwide Questionnaire Survey of Mental Health and Psychiatric Institutions. Int J Environ Res Public Health. 2021;18(14).

10. Penninx BWJH, Benros ME, Klein RS, Vinkers CH. How COVID-19 shaped mental health: from infection to pandemic effects. Nature Medicine. 2022;28(10):2027-37.

11. Overhage L, Hailu R, Busch AB, Mehrotra A, Michelson KA, Huskamp HA. Trends in Acute Care Use for Mental Health Conditions Among Youth During the COVID-19 Pandemic. JAMA Psychiatry. 2023;80(9):924-32.

12. Otto AK, Jary JM, Sturza J, Miller CA, Prohaska N, Bravender T, et al. Medical Admissions Among Adolescents With Eating Disorders During the COVID-19 Pandemic. Pediatrics. 2021;148(4).

13. Hill RM, Rufino K, Kurian S, Saxena J, Saxena K, Williams L. Suicide Ideation and Attempts in a Pediatric Emergency Department Before and During COVID-19. Pediatrics. 2021;147(3).

14. Gutiérrez-Sacristán A, Serret-Larmande A, Hutch MR, Sáez C, Aronow BJ, Bhatnagar S, et al. Hospitalizations Associated With Mental Health Conditions Among Adolescents in the US and France During the COVID-19 Pandemic. JAMA Netw Open. 2022;5(12):e2246548.

15. Kuitunen I, Uimonen MM, Ponkilainen VT, Mattila VM. Primary care visits due to mental health problems and use of psychotropic medication during the COVID-19 pandemic in Finnish adolescents and young adults. Child Adolesc Psychiatry Ment Health. 2023;17(1):35.

16. Chan VKY, Chai Y, Chan SSM, Luo H, Jit M, Knapp M, et al. Impact of COVID-19 pandemic on depression incidence and healthcare service use among patients with depression: an interrupted time-series analysis from a 9-year population-based study. BMC Med. 2024;22(1):169.

17. De Bandt D, Haile SR, Devillers L, Bourrion B, Menges D. Prescriptions of antidepressants and anxiolytics in France 2012-2022 and changes with the COVID-19 pandemic: interrupted time series analysis. BMJ Ment Health. 2024;27(1).

18. Bliddal M, Rasmussen L, Andersen JH, Jensen PB, Pottegård A, Munk-Olsen T, et al. Psychotropic Medication Use and Psychiatric Disorders During the COVID-19 Pandemic Among Danish Children, Adolescents, and Young Adults. JAMA Psychiatry. 2023;80(2):176-80.

19. Valtuille Z, Acquaviva E, Trebossen V, Ouldali N, Bourmaud A, Sclison S, et al. Psychotropic Medication Prescribing for Children and Adolescents After the Onset of the COVID-19 Pandemic. JAMA Netw Open. 2024;7(4):e247965.

20. Yoshioka E, Hanley SJB, Sato Y, Saijo Y. Impact of the COVID-19 pandemic on suicide rates in Japan through December 2021: An interrupted time series analysis. Lancet Reg Health West Pac. 2022;24:100480.

21. Ewusie JE, Soobiah C, Blondal E, Beyene J, Thabane L, Hamid JS. Methods, Applications and Challenges in the Analysis of Interrupted Time Series Data: A Scoping Review. J Multidiscip Healthc. 2020;13:411-23.

22. Linden A. Conducting Interrupted Time-series Analysis for Single- and Multiple-group Comparisons. The Stata Journal. 2015;15(2):480-500.

23. Linden A, Adams JL. Applying a propensity score-based weighting model to interrupted time series data: improving causal inference in programme evaluation. J Eval Clin Pract. 2011;17(6):1231-8.

Supplementary Table 1. Catalog of Medication Classes and Agents Prescribed in Japan for Children, Teenagers, and Young Adults from February 2016 through November 2022.

MEDICATION CLASS	DRUG PRESCRIBING ATC CODES & AGENTS
**Antipsychotics**	N05AA01 (chlorpromazine), N05AA02 (levomepromazine), N05AB02 (fluphenazine), N05AB03 (perphenazine), N05AB04 (prochlorperazine), N05AC01 (periciazine), N05AD01 (haloperidol), N05AD05 (pipamperone), N05AD06 (bromperidol), N05AD08 (droperidol), N05AE01 (oxypertine), N05AE05 (lurasidone), N05AG02 (pimozide), N05AH02 (clozapine), N05AH03 (olanzapine), N05AH04 (quetiapine), N05AH05 (asenapine), N05AL01 (sulpiride), N05AL02 (sultopride), N05AL03 (tiapride), N05AN01 (lithium), N05AX08 (risperidone), N05AX10 (mosapramine), N05AX11 (zotepine), N05AX12 (aripiprazole), N05AX13 (paliperidone), N05AX16 (brexpiprazole)
**Anxiolytics**	N05BA01 (diazepam), N05BA02 (chlordiazepoxide), N05BA03 (medazepam), N05BA05 (potassium clorazepate), N05BA06 (lorazepam), N05BA08 (bromazepam), N05BA09 (clobazam), N05BA12 (alprazolam), N05BA17 (fludiazepam), N05BA18 (ethyl loflazepate), N05BA19 (etizolam), N05BA21 (clotiazepam), N05BA22 (cloxazolam), N05BA23 (tofisopam), N05BA25 (mexazolam), N05BB01 (hydroxyzine), N05BE (buspirone)
**Hypnotics and sedatives**	N05CA01 (pentobarbital), N05CA02 (amobarbital), N05CA04 (barbital), N05CA06 (secobarbital), N05CC01 (chloral hydrate), N05CD01 (flurazepam), N05CD02 (nitrazepam), N05CD03 (flunitrazepam), N05CD04 (estazolam), N05CD05 (triazolam), N05CD06 (lormetazepam), N05CD08 (midazolam), N05CD09 (brotizolam), N05CD10 (quazepam), N05CD14 (remimazolam), N05CD15 (nimetazepam), N05CF01 (zopiclone), N05CF02 (zolpidem), N05CF04 (eszopiclone), N05CH02 (ramelteon), N05CM03 (bromisoval), N05CM05 (scopolamine), N05CM07 (triclofos), N05CM09 (Valerianae radix), N05CM11 (bromides), N05CM18 (dexmedetomidine), N05CM19 (suvorexant), N05CM21 (lemborexant)

**Antidepressants**	N06AA02 (imipramine), N06AA04 (clomipramine), N06AA06 (trimipramine), N06AA07 (lofepramine), N06AA09 (amitriptyline), N06AA10 (nortriptyline), N06AA16 (dosulepin), N06AA17 (amoxapine), N06AA21 (maprotiline), N06AB05 (paroxetine), N06AB06 (sertraline), N06AB08 (fluvoxamine), N06AB10 (escitalopram), N06AX03 (mianserin), N06AX05 (trazodone), N06AX11 (mirtazapine), N06AX16 (venlafaxine), N06AX17 (milnacipran), N06AX21 (duloxetine), N06AX26 (vortioxetine)
**Psychostimulants**	N06BA04 (methylphenidate), N06BA05 (pemoline), N06BA07 (modafinil), N06BA09 (atomoxetine), N06BA12 (lisdexamfetamine), N06BC01 (caffeine), N06BX01 (meclofenoxate), N06BX03 (piracetam), N06BX06 (citicoline)

##

The R code provided in the supplementary files includes source code for interrupted time-series analysis and graphing. The code runs successfully in R version 4.4.1 on Windows 11 Home.

Wenbo Huang (wenbohuang@g.ecc.u-tokyo.ac.jp)

##

```
# Load necessary libraries

library(lubridate)      # For working with date-time data

library(tsModel)        # For time-series modeling

library(readxl)         # For reading Excel files

library(ggplot2)        # For creating plots and visualizations

library(car)            # For the Durbin-Watson test

library(orcutt)         # For Cochrane-Orcutt estimation

library(nlme)           # For generalized least squares (GLS)

library(prais)          # For Prais-Winsten estimation

library(sandwich)       # For Newey-West standard errors

library(ggthemes)       # For ggplot themes

library(lmtest)         # For testing linear models

library(lme4)           # For linear mixed-effects models
```

```
library(lmerTest)       # For REML with Satterthwaite approximation
library(forecast)       # For ARIMA modeling

# Read data from an Excel file
data <- read_excel("data.xls")

# Ensure 'Month' is a factor for seasonality adjustment
data$Month <- month(ym(data$Year))
# ----------------------------
# Linear Model with Seasonality
# ----------------------------
mod1 <- lm(Outcome ~ Time + Intervention + Post + factor(Month), data = data)
summary(mod1)
confint(mod1, level = 0.95)

# ----------------------------
# Durbin-Watson Test for Autocorrelation
# ----------------------------
durbinWatsonTest(mod1)
```

```
# ----------------------------
# Cochrane-Orcutt Model with Seasonality
# ----------------------------
mod2 <- cochrane.orcutt(mod1)
summary(mod2)
coef_estimates <- summary(mod2)$coefficients[, 1]
std_errors <- summary(mod2)$coefficients[, 2]
ci_lower <- coef_estimates - 1.96 * std_errors
ci_upper <- coef_estimates + 1.96 * std_errors
ci_mod2 <- cbind(coef_estimates, ci_lower, ci_upper)
colnames(ci_mod2) <- c("Estimate", "CI Lower (95%)", "CI Upper (95%)")
ci_mod2

# ----------------------------
# OLS with Newey-West Standard Errors and Seasonality
# ----------------------------
ols_model <- lm(Outcome ~ Time + Intervention + Post + factor(Month), data = data)
summary(ols_model)
```

```
coeftest(ols_model, vcov = NeweyWest(ols_model))

# ----------------------------
# Prais-Winsten Model with Seasonality
# ----------------------------
prais_model <- prais_winsten(Outcome ~ Time + Intervention + Post + factor(Month), data = data)
summary(prais_model)

# ----------------------------
# REML (Linear Mixed Effects Model) with Seasonality
# ----------------------------
reml_model <- lmer(Outcome ~ Time + Intervention + Post + factor(Month) + (1 | Year), REML = TRUE, data = data)
summary(reml_model)

# ----------------------------
# REML with Satterthwaite Approximation
# ----------------------------
reml_satterthwaite_model <- lmer(Outcome ~ Time + Intervention + Post + factor(Month) + (1 | Year), REML = TRUE, data = data)
summary(reml_satterthwaite_model)
```

```
# ----------------------------
# ARIMA with Exogenous Regressors including Seasonality
# ----------------------------
# For simplicity, encode month dummies (one-hot encoding for ARIMA)
month_dummies <- model.matrix(~ Month - 1, data = data)
xreg_matrix <- cbind(data$Time, data$Intervention, data$Post, month_dummies)

arima_model <- auto.arima(data$Outcome, xreg = xreg_matrix)
summary(arima_model)

# ----------------------------
# Counterfactual Estimates Using Pre-Intervention Data
# ----------------------------
pre_intervention <- subset(data, Intervention == 0)
model_counter <- lm(Outcome ~ Time + Month, data = pre_intervention)
data$Counterfactual <- predict(model_counter, newdata = data)
# Plotting the results including the actual data and the counterfactual scenario
plot <- ggplot(data, aes(x = Time)) +
```

```
geom_line(aes(y = Outcome), color = "#4E79A7", size = 1.0, linetype = "solid") +  # Actual data line
geom_point(aes(y = Outcome), color = "#E15759", size = 1.5, shape = 16) +         # Actual data points
geom_line(aes(y = Counterfactual), color = "#76B7B2", size = 1.0, linetype = "dashed") +  # Counterfactual line
geom_vline(xintercept = 47, linetype = "dotted", color = "black", size = 1.0) +  # Intervention line
annotate("text", x = 47, y = max(data$Outcome), label = "Covid-19", vjust = -1.5, color = "black", size = 5) +  # Label the intervention
labs(
  title = "Interrupted Time Series Analysis with Counterfactual Scenario",
  subtitle = "Examining the Effect of Covid-19",
  x = "Time (Year-Month)",
  y = "Number of Patients Prescribed Psychotropic Medications",
  caption = "Data Source: Data Dataset"
) +
theme_minimal(base_size = 15) +
theme(
  plot.title = element_text(hjust = 0.5, face = "plain", size = 13, color = "#333333"),  # Title styling
  plot.subtitle = element_text(hjust = 0.5, size = 10, color = "#555555"),  # Subtitle styling
  axis.title = element_text(face = "plain", size = 12),  # Axis title styling
  axis.text = element_text(size = 12),  # Axis text styling
  panel.grid.major = element_line(color = "#CCCCCC", size = 0.5),  # Grid styling
```

```
    panel.grid.minor = element_blank(),  # Hide minor grid lines
    legend.position = "bottom",  # Legend position
    legend.title = element_blank(),  # Hide legend title
    legend.text = element_text(size = 12)  # Legend text styling
  )
```

# Save the plot as an image file

```
ggsave("plot.jpg", plot = plot, width = 10, height = 6, dpi = 800)
```

# Read a dataset for further analysis

```
data <- read_excel("data.xls")

# Run a linear regression model
mod1 <- lm(Outcome ~ Time + Intervention + Post + factor(Month), data = data)

# Split the dataset by sex
data_male <- data[data$Sex == 1, ]
data_female <- data[data$Sex == 2, ]
```

```
# Fit the ITS model for males
its_model_male <- lm(Outcome ~ Time + Intervention + Post + factor(Month), data = data_male)
summary(its_model_male)

# Fit the ITS model for females
its_model_female <- lm(Outcome ~ Time + Intervention + Post + factor(Month), data = data_female)
summary(its_model_female)

# Plot the results for males
ggplot(data_male, aes(x = Time, y = Outcome)) +
  geom_point() +
  geom_smooth(method = "lm", formula = y ~ x + Intervention + Post + factor(Month)) +
  theme_minimal() +
  labs(title = "Interrupted Time Series Analysis for Males",
       x = "Time",
       y = "Outcome")

# Plot the results for females
ggplot(data_female, aes(x = Time, y = Outcome)) +
```

```
  geom_point() +
  geom_smooth(method = "lm", formula = y ~ x + Intervention + Post + Month) +
  theme_minimal() +
  labs(title = "Interrupted Time Series Analysis for Females",
       x = "Time",
       y = "Outcome")

# Conduct subgroup analysis for males
robust_se_male <- coeftest(its_model_male, vcov = vcovHC(its_model_male, type = "HC1"))
print(robust_se_male)

# Conduct subgroup analysis for females
robust_se_female <- coeftest(its_model_female, vcov = vcovHC(its_model_female, type = "HC1"))
print(robust_se_female)

# Extract fitted values and residuals from the model
data$Fitted <- fitted(mod1)
data$Residuals <- resid(mod1)
```

```r
# Plot residuals vs fitted values to check model assumptions
residual_plot <- ggplot(data, aes(x = Fitted, y = Residuals)) +
  geom_point(color = "#4E79A7", size = 2) +  # Plot residuals as blue points
  geom_hline(yintercept = 0, linetype = "dashed", color = "red") +  # Add a horizontal red dashed line at zero residuals
  labs(
    title = "Residuals vs Fitted",
    x = "Fitted Values",
    y = "Residuals"
  ) +
  theme_minimal(base_size = 15) +
  theme(
    plot.title = element_text(hjust = 0.5, face = "bold", size = 16),  # Title styling
    axis.title = element_text(face = "bold", size = 14),  # Axis title styling
    axis.text = element_text(size = 12)  # Axis text styling
  )

# Print the residual plot
print(residual_plot)
```

```r
# Save the residual plot as an image file
ggsave("Residuals_vs_Fitted.jpg", plot = residual_plot, width = 8, height = 6, dpi = 800)

# Plot residuals over time
residuals_time_plot <- ggplot(data, aes(x = Time, y = Residuals)) +
  geom_line(color = "#76B7B2", size = 1.0) +
  geom_hline(yintercept = 0, linetype = "dashed", color = "red") +
  geom_vline(xintercept = 47, linetype = "dotted", color = "black", size = 1.0) +
  labs(
    title = "Residuals Over Time",
    x = "Time (Year-Month)",
    y = "Residuals",
    caption = "Data Source: Data Dataset"
  ) +
  theme_minimal(base_size = 15)

# Save the plot
ggsave("Residuals_Over_Time.jpg", plot = residuals_time_plot, width = 10, height = 6, dpi = 800)
```

```r
# Plot distribution of residuals
residuals_distribution_plot <- ggplot(data, aes(x = Residuals)) +
  geom_histogram(binwidth = 0.5, fill = "#4E79A7", color = "black", alpha = 0.7) +
  geom_density(color = "#E15759", size = 1.0) +
  labs(
    title = "Distribution of Residuals",
    x = "Residuals",
    y = "Density",
    caption = "Data Source: Data Dataset"
  ) +
  theme_minimal(base_size = 15)

# Save the plot
ggsave("Residuals_Distribution.jpg", plot = residuals_distribution_plot, width = 10, height = 6, dpi = 800)

# Plot the Autocorrelation Function (ACF) of residuals
acf_plot <- ggAcf(data$Residuals) +
  ggtitle("ACF of Residuals") +
  theme_minimal(base_size = 15) +
```

```r
  theme(
    plot.title = element_text(hjust = 0.5, size = 16, face = "bold"),
    axis.title = element_text(face = "bold", size = 14)
  )

# Save the ACF plot
ggsave("ACF_Plot.jpg", plot = acf_plot, width = 8, height = 6, dpi = 800)

# Plot the Cumulative Impact Plot
data$CumulativeImpact <- cumsum(data$Outcome - data$Counterfactual)

cumulative_impact_plot <- ggplot(data, aes(x = Time, y = CumulativeImpact)) +
  geom_line(color = "#76B7B2", size = 1.0) +
  geom_vline(xintercept = 47, linetype = "dotted", color = "black", size = 1.0) +
  annotate("text", x = 47, y = max(data$CumulativeImpact), label = "Covid-19", vjust = -1.5, color = "black", size = 5) +
  labs(
    title = "Cumulative Impact of Intervention Over Time",
    x = "Time (Year-Month)",
    y = "Cumulative Impact",
```

```
        caption = "Data Source: Data Dataset"
    ) +
    theme_minimal(base_size = 15)

ggsave("Cumulative_Impact.jpg", plot = cumulative_impact_plot, width = 10, height = 6, dpi = 800)

# Segmented Regression Plot
segmented_plot <- ggplot(data, aes(x = Time, y = Outcome)) +
    geom_smooth(method = "lm", se = TRUE, color = "#4E79A7", size = 1.0) +
    geom_vline(xintercept = 47, linetype = "dotted", color = "black", size = 1.0) +
    annotate("text", x = 47, y = max(data$Outcome), label = "Covid-19", vjust = -1.5, color = "black", size = 5) +
    labs(
        title = "Segmented Regression Analysis",
        x = "Time (Year-Month)",
        y = "Outcome",
        caption = "Data Source: Data Dataset"
    ) +
    theme_minimal(base_size = 15)
```

```
ggsave("Segmented_Regression.jpg", plot = segmented_plot, width = 10, height = 6, dpi = 800)
```

Supplementary Table 2. Introductions for Variables Used in Models.

Variables used in models	Introductions
**drug_code**	Character variable, including a receipt medicine code with 9 characters, starting with the number 6.
**atc_code**	Character variable, including the ATC code with 7 characters, as defined by WHO and EPHMRA.
**receipt_ym**	Character variable, including the year and month information of the receipt, recorded in YYYY/MM format.
**kojin_id**	Integer variable. Each patient with a prescription is assigned one Kojin_id.
**receipt_id**	Integer variable. Each receipt is assigned one receipt_id.
**birth_ym**	Character variable, including the patient's birth year and month information in YYYY/MM format.
**observable_start_ym**	Character variable, representing the start of the observable period for each individual, formatted in YYYY/MM style.
**observable_end_ym**	Character variable, representing the end of the observable period for each individual, formatted in YYYY/MM style.
**sex_code**	Character variable: 1 for male, 2 for female.
**age**	Numerical variable, calculated from "**birth_ym**" and "**observable_end_ym**".
**patient_count**	Numerical variable, calculated from "**kojin_id**". This variable indicates the monthly recorded number of outpatients for psychotropic medication prescriptions.
**prescription_count**	Numerical variable, calculated from "**drug_code**". This variable indicates the monthly recorded counts of psychotropic medication prescriptions.
**month**	Time variable, calculated from "**receipt_ym**". This is the time variable for ITS modeling calculation, with YYYY/MM style.

Note: Abbreviations used: ATC (Anatomical Therapeutic Chemical Classification System), WHO (World Health Organization), and EPHMRA (European Pharmaceutical Market Research Association).

```
###################################################
Main ITS Analysis Results for the Number of Patients Prescribed Antidepressants.
###################################################
Residuals:
    Min      1Q  Median      3Q     Max
-26.4909  -6.0490   0.8336   6.9247  20.3742


Coefficients:
              Estimate Std. Error t value Pr(>|t|)
(Intercept)     59.7369     5.8682  10.180  3.1e-15 ***
Time             2.4894     0.1219  20.417  < 2e-16 ***
Intervention    -1.4917     5.2524  -0.284   0.7773
Post             6.5660     0.2349  27.958  < 2e-16 ***
factor(Month)2   -8.1147     6.4589  -1.256   0.2134
factor(Month)3   -2.8466     6.4447  -0.442   0.6601
factor(Month)4  -14.5785     6.4324  -2.266   0.0267 *
factor(Month)5   -9.7390     6.4222  -1.516   0.1341
factor(Month)6   -3.0424     6.4139  -0.474   0.6368
```

```
factor(Month)7    2.7972     6.4076   0.437   0.6638
factor(Month)8  -14.5062     6.4033  -2.265   0.0267 *
factor(Month)9   -9.5238     6.4010  -1.488   0.1415
factor(Month)10   0.8871     6.4007   0.139   0.8902
factor(Month)11  -3.5591     6.4024  -0.556   0.5801
factor(Month)12   5.0114     6.6264   0.756   0.4521
---
Signif. codes:  0 '***' 0.001 '**' 0.01 '*' 0.05 '.' 0.1 ' ' 1

Residual standard error: 11.48 on 67 degrees of freedom
Multiple R-squared:  0.9927,     Adjusted R-squared:  0.9912
F-statistic:   650 on 14 and 67 DF,  p-value: < 2.2e-16


> durbinWatsonTest(mod1)
 lag Autocorrelation D-W Statistic p-value
   1       0.2038118      1.501761   0.018
 Alternative hypothesis: rho != 0
```

```
> mod2 <- cochrane.orcutt(mod1)

> summary(mod2)

Call:

lm(formula = Outcome ~ Time + Intervention + Post + factor(Month),

    data = data)


                Estimate Std. Error t value  Pr(>|t|)
(Intercept)      58.46314    6.59798   8.861 7.688e-13 ***
Time             2.52517    0.15819  15.963 < 2.2e-16 ***
Intervention     -1.23622    6.43728  -0.192   0.84830
Post             6.47116    0.29779  21.731 < 2.2e-16 ***
factor(Month)2   -9.63832    5.91986  -1.628   0.10826
factor(Month)3   -2.93638    6.31908  -0.465   0.64369
factor(Month)4  -14.33806    6.43659  -2.228   0.02932 *
factor(Month)5   -9.41962    6.45316  -1.460   0.14912
factor(Month)6   -2.70128    6.44729  -0.419   0.67659
factor(Month)7    3.14689    6.43836   0.489   0.62662
factor(Month)8  -14.15076    6.43006  -2.201   0.03126 *
```

factor(Month)9   -9.16338   6.41979 -1.427   0.15819

factor(Month)10   1.25243   6.39169   0.196   0.84525

factor(Month)11 -3.18895   6.27390 -0.508   0.61295

factor(Month)12   4.26372   5.83349   0.731   0.46742

Signif. codes: 0 ‘***’ 0.001 ‘**’ 0.01 ‘*’ 0.05 ‘.’ 0.1 ‘ ’ 1

Residual standard error: 10.4453 on 76 degrees of freedom

Multiple R-squared: 0.9885 , Adjusted R-squared: 0.9879

F-statistic: 404.8 on 4 and 76 DF, p-value: < 3.182e-58

Durbin-Watson statistic

(original):   1.50176 , p-value: 8.329e-03

(transformed): 1.82108 , p-value: 1.575e-01>

> coef_estimates <- summary(mod2)$coefficients[, 1]

> std_errors <- summary(mod2)$coefficients[, 2]

> ci_lower <- coef_estimates - 1.96 * std_errors

> ci_upper <- coef_estimates + 1.96 * std_errors

```
> ci_mod2 <- cbind(coef_estimates, ci_lower, ci_upper)
> colnames(ci_mod2) <- c("Estimate", "CI Lower (95%)", "CI Upper (95%)")
> ci_mod2
                 Estimate CI Lower (95%) CI Upper (95%)
(Intercept)     58.463135      45.531103      71.395168
Time             2.525175       2.215121       2.835228
Intervention    -1.236216     -13.853284      11.380852
Post             6.471160       5.887494       7.054827
factor(Month)2  -9.638316     -21.241236       1.964605
factor(Month)3  -2.936380     -15.321771       9.449012
factor(Month)4 -14.338058     -26.953773      -1.722342
factor(Month)5  -9.419624     -22.067809       3.228561
factor(Month)6  -2.701277     -15.337959       9.935404
factor(Month)7   3.146894      -9.472282      15.766069
factor(Month)8 -14.150760     -26.753683      -1.547838
factor(Month)9  -9.163376     -21.746172       3.419420
factor(Month)10  1.252426     -11.275277      13.780130
factor(Month)11 -3.188950     -15.485786       9.107886
```

factor(Month)12	4.263723	-7.169908	15.697354

```
####################################################

Main ITS Analysis Results for the Number of Patients Prescribed Antipsychotics.

####################################################

Residuals:
    Min      1Q  Median      3Q     Max 
-69.788  -7.650   1.208  10.709  40.834 


Coefficients:
                Estimate Std. Error t value Pr(>|t|)    
(Intercept)     170.4774    10.5547  16.152  < 2e-16 ***
Time              6.8005     0.2193  31.010  < 2e-16 ***
Intervention    -27.7353     9.4472  -2.936  0.00455 ** 
Post              4.4079     0.4224  10.435 1.11e-15 ***
factor(Month)2  -13.7374    11.6172  -1.183  0.24119    
factor(Month)3   14.5730    11.5916   1.257  0.21305    
factor(Month)4  -11.9738    11.5696  -1.035  0.30442    
factor(Month)5  -24.8063    11.5511  -2.148  0.03537 *  
factor(Month)6    0.3612    11.5362   0.031  0.97512    
```

```
factor(Month)7   12.3858    11.5249   1.075  0.28636
factor(Month)8  -14.8752    11.5171  -1.292  0.20094
factor(Month)9   -5.5649    11.5130  -0.483  0.63042
factor(Month)10   8.3169    11.5124   0.722  0.47254
factor(Month)11  -0.3727    11.5155  -0.032  0.97428
factor(Month)12  23.1032    11.9185   1.938  0.05679 .
---
Signif. codes:  0 '***' 0.001 '**' 0.01 '*' 0.05 '.' 0.1 ' ' 1

Residual standard error: 20.64 on 67 degrees of freedom
Multiple R-squared:  0.9904,	Adjusted R-squared:  0.9884
F-statistic: 493.8 on 14 and 67 DF,  p-value: < 2.2e-16

durbinWatsonTest(mod1)
 lag Autocorrelation D-W Statistic p-value
   1       0.2572278      1.348087   0.002
 Alternative hypothesis: rho != 0
> mod2 <- cochrane.orcutt(mod1)
```

```
> summary(mod2)

Call:

lm(formula = Outcome ~ Time + Intervention + Post + factor(Month),

    data = data)


                Estimate Std. Error t value  Pr(>|t|)

(Intercept)     167.42444   12.42296  13.477 < 2.2e-16 ***

Time             6.87224    0.30968  22.191 < 2.2e-16 ***

Intervention    -25.33391   12.36540  -2.049   0.04446 *

Post             4.11941    0.58172   7.081  1.16e-09 ***

factor(Month)2  -17.25000   10.12777  -1.703   0.09323 .

factor(Month)3   14.13567   11.13686   1.269   0.20880

factor(Month)4  -11.42885   11.49721  -0.994   0.32383

factor(Month)5  -23.92314   11.59164  -2.064   0.04297 *

factor(Month)6    1.38438   11.60248   0.119   0.90539

factor(Month)7   13.48807   11.58957   1.164   0.24869

factor(Month)8  -13.71273   11.56734  -1.185   0.24008

factor(Month)9   -4.34787   11.52591  -0.377   0.70722
```

```
factor(Month)10   9.58664   11.41507   0.840   0.40404
factor(Month)11   0.94918   11.06043   0.086   0.93187
factor(Month)12  21.10805    9.93600   2.124   0.03739 *
---
Signif. codes:  0 ‘***’ 0.001 ‘**’ 0.01 ‘*’ 0.05 ‘.’ 0.1 ‘ ’ 1

Residual standard error: 18.3759 on 76 degrees of freedom
Multiple R-squared:  0.9818 ,  Adjusted R-squared:  0.9809
F-statistic: 254.6 on 4 and 76 DF,  p-value: < 1.077e-51

Durbin-Watson statistic
(original):    1.34809 , p-value: 1.01e-03
(transformed): 1.91947 , p-value: 2.787e-01> coef_estimates <- summary(mod2)$coefficients[, 1]
> std_errors <- summary(mod2)$coefficients[, 2]
> ci_lower <- coef_estimates - 1.96 * std_errors
> ci_upper <- coef_estimates + 1.96 * std_errors
> ci_mod2 <- cbind(coef_estimates, ci_lower, ci_upper)
> colnames(ci_mod2) <- c("Estimate", "CI Lower (95%)", "CI Upper (95%)")
```

```
> ci_mod2
                 Estimate CI Lower (95%) CI Upper (95%)
(Intercept)     167.4244393     143.075441     191.773437
Time             6.8722440       6.265264       7.479224
Intervention    -25.3339116     -49.570098      -1.097725
Post             4.1194143       2.979249       5.259580
factor(Month)2  -17.2499966     -37.100421       2.600428
factor(Month)3   14.1356664      -7.692571      35.963904
factor(Month)4  -11.4288541     -33.963381      11.105673
factor(Month)5  -23.9231441     -46.642755      -1.203533
factor(Month)6    1.3843818     -21.356488      24.125252
factor(Month)7   13.4880668      -9.227500      36.203634
factor(Month)8  -13.7127280     -36.384722       8.959266
factor(Month)9   -4.3478685     -26.938654      18.242917
factor(Month)10   9.5866426     -12.786902      31.960187
factor(Month)11   0.9491784     -20.729260      22.627617
factor(Month)12  21.1080459       1.633486      40.582605
```

###

Main ITS Analysis Results for the Number of Patients Prescribed Anxiolytics.

###

```
Residuals:
   Min     1Q Median     3Q    Max
-50.33 -12.35  -2.26  13.42  43.37


Coefficients:
               Estimate Std. Error t value Pr(>|t|)
(Intercept)    383.4929    11.4264  33.562  < 2e-16 ***
Time            -1.9382     0.2374  -8.164 1.22e-11 ***
Intervention   -33.9611    10.2274  -3.321  0.00146 **
Post             7.7470     0.4573  16.941  < 2e-16 ***
factor(Month)2   6.3631    12.5767   0.506  0.61456
factor(Month)3  18.4098    12.5490   1.467  0.14705
factor(Month)4  -4.8293    12.5251  -0.386  0.70104
factor(Month)5  -4.4969    12.5051  -0.360  0.72027
factor(Month)6   5.2641    12.4890   0.421  0.67474
```

```
factor(Month)7   24.0250    12.4767   1.926  0.05840 .
factor(Month)8    4.6431    12.4683   0.372  0.71078
factor(Month)9   -5.3102    12.4638  -0.426  0.67144
factor(Month)10  16.7364    12.4632   1.343  0.18385
factor(Month)11   5.6402    12.4666   0.452  0.65242
factor(Month)12  40.4774    12.9029   3.137  0.00254 **
---
Signif. codes:  0 ‘***’ 0.001 ‘**’ 0.01 ‘*’ 0.05 ‘.’ 0.1 ‘ ’ 1

Residual standard error: 22.35 on 67 degrees of freedom
Multiple R-squared:  0.8272,	Adjusted R-squared:  0.7911
F-statistic: 22.92 on 14 and 67 DF,  p-value: < 2.2e-16

> durbinWatsonTest(mod1)
 lag Autocorrelation D-W Statistic p-value
   1       0.3475059      1.225063       0
 Alternative hypothesis: rho != 0
> mod2 <- cochrane.orcutt(mod1)
```

```
> summary(mod2)  # Display summary of the Cochrane-Orcutt model

Call:

lm(formula = Outcome ~ Time + Intervention + Post + factor(Month),

    data = data)


                Estimate Std. Error t value  Pr(>|t|)

(Intercept)     379.06781   13.81283  27.443 < 2.2e-16 ***

Time            -1.83098    0.35496  -5.158 2.468e-06 ***

Intervention    -30.22835   13.87028  -2.179 0.0328787 *

Post            7.36575    0.66570  11.065 < 2.2e-16 ***

factor(Month)2    0.60360   10.29662   0.059 0.9534311

factor(Month)3   16.81845   11.58518   1.452 0.1513162

factor(Month)4   -4.82320   12.11093  -0.398 0.6917299

factor(Month)5   -3.85700   12.29100  -0.314 0.7546573

factor(Month)6    6.17661   12.33713   0.501 0.6182815

factor(Month)7   25.07485   12.33164   2.033 0.0460392 *

factor(Month)8    5.77950   12.29734   0.470 0.6399189

factor(Month)9   -4.10630   12.21909  -0.336 0.7378943
```

```
factor(Month)10  18.00077   12.02649   1.497 0.1392230

factor(Month)11   6.96231   11.50973   0.605 0.5473153

factor(Month)12  39.06186   10.05652   3.884 0.0002403 ***

---

Signif. codes:  0 ‘***’ 0.001 ‘**’ 0.01 ‘*’ 0.05 ‘.’ 0.1 ‘ ’ 1


Residual standard error: 19.0822 on 76 degrees of freedom

Multiple R-squared:  0.723 ,  Adjusted R-squared:  0.7084

F-statistic: 12.3 on 4 and 76 DF,  p-value: < 2.003e-13


Durbin-Watson statistic

(original):    1.22506 , p-value: 1.241e-04

(transformed): 1.88906 , p-value: 2.389e-01> coef_estimates <- summary(mod2)$coefficients[, 1]

> std_errors <- summary(mod2)$coefficients[, 2]

> ci_lower <- coef_estimates - 1.96 * std_errors

> ci_upper <- coef_estimates + 1.96 * std_errors

> ci_mod2 <- cbind(coef_estimates, ci_lower, ci_upper)

> colnames(ci_mod2) <- c("Estimate", "CI Lower (95%)", "CI Upper (95%)")
```

```
> ci_mod2
                  Estimate CI Lower (95%) CI Upper (95%)
(Intercept)     379.0678064    351.9946674     406.140945
Time            -1.8309779     -2.5267064      -1.135249
Intervention   -30.2283521    -57.4140924      -3.042612
Post             7.3657525      6.0609759       8.670529
factor(Month)2   0.6035985    -19.5777803      20.784977
factor(Month)3  16.8184543     -5.8884960      39.525405
factor(Month)4  -4.8232011    -28.5606211      18.914219
factor(Month)5  -3.8570010    -27.9473531      20.233351
factor(Month)6   6.1766090    -18.0041627      30.357381
factor(Month)7  25.0748501      0.9048288      49.244871
factor(Month)8   5.7794959    -18.3232832      29.882275
factor(Month)9  -4.1063044    -28.0557171      19.843108
factor(Month)10 18.0007673     -5.5711557      41.572690
factor(Month)11  6.9623102    -15.5967607      29.521381
factor(Month)12 39.0618613     19.3510825      58.772640
```

```
##################################################
Main ITS Analysis Results for the Number of Patients Prescribed Hypnotics and Sedatives.
##################################################
Residuals:
    Min      1Q  Median      3Q     Max
-46.266 -11.788  -1.488  11.614  44.281


Coefficients:
               Estimate Std. Error t value Pr(>|t|)
(Intercept)    184.9477     9.9357  18.614  < 2e-16 ***
Time             1.0577     0.2064   5.124 2.74e-06 ***
Intervention     5.9385     8.8931   0.668  0.50658
Post             6.9967     0.3976  17.595  < 2e-16 ***
factor(Month)2   -6.7548    10.9359  -0.618  0.53888
factor(Month)3   19.0460    10.9118   1.745  0.08549 .
factor(Month)4   -1.8674    10.8911  -0.171  0.86437
factor(Month)5  -10.2095    10.8737  -0.939  0.35115
factor(Month)6    5.7342    10.8596   0.528  0.59922
```

```
factor(Month)7   22.8208    10.8490   2.103  0.03918 *
factor(Month)8   29.4787    10.8417   2.719  0.00833 **
factor(Month)9   21.8510    10.8378   2.016  0.04779 *
factor(Month)10  22.2233    10.8373   2.051  0.04422 *
factor(Month)11  10.0241    10.8401   0.925  0.35843
factor(Month)12  18.5566    11.2195   1.654  0.10281
---
Signif. codes:  0 '***' 0.001 '**' 0.01 '*' 0.05 '.' 0.1 ' ' 1

Residual standard error: 19.43 on 67 degrees of freedom
Multiple R-squared:  0.9689,	Adjusted R-squared:  0.9624
F-statistic: 149.2 on 14 and 67 DF,  p-value: < 2.2e-16

> durbinWatsonTest(mod1)
 lag Autocorrelation D-W Statistic p-value
   1        0.569687     0.7920996       0
 Alternative hypothesis: rho != 0
```

```
> mod2 <- cochrane.orcutt(mod1)

> summary(mod2)

Call:

lm(formula = Outcome ~ Time + Intervention + Post + factor(Month),

    data = data)


                 Estimate Std. Error t value  Pr(>|t|)

(Intercept)     172.09129   13.82645  12.447 < 2.2e-16 ***

Time              1.48704    0.38466   3.866 0.0002555 ***

Intervention     -4.73712   13.09121  -0.362 0.7186167

Post              6.61819    0.71910   9.203 1.895e-13 ***

factor(Month)2  -11.85148    7.14392  -1.659 0.1018692

factor(Month)3   17.23484    8.53072   2.020 0.0474111 *

factor(Month)4   -1.86226    9.29320  -0.200 0.8417921

factor(Month)5   -9.24952    9.69952  -0.954 0.3437628

factor(Month)6    7.14364    9.89095   0.722 0.4726979

factor(Month)7   24.38333    9.93855   2.453 0.0167994 *

factor(Month)8   31.02067    9.86678   3.144 0.0024980 **
```

```
factor(Month)9   23.27037    9.65975   2.409 0.0187948 *
factor(Month)10  23.46030    9.25130   2.536 0.0135913 *
factor(Month)11  11.04374    8.48511   1.302 0.1975971
factor(Month)12  18.48673    6.84079   2.702 0.0087421 **
---
Signif. codes:  0 ‘***’ 0.001 ‘**’ 0.01 ‘*’ 0.05 ‘.’ 0.1 ‘ ’ 1

Residual standard error: 13.9676 on 76 degrees of freedom
Multiple R-squared:  0.9133 ,  Adjusted R-squared:  0.9087
F-statistic: 49.7 on 4 and 76 DF,  p-value: < 1.734e-29

Durbin-Watson statistic
(original):    0.79210 , p-value: 1.295e-09
(transformed): 2.17372 , p-value: 6.959e-01> coef_estimates <- summary(mod2)$coefficients[, 1]
> std_errors <- summary(mod2)$coefficients[, 2]
> ci_lower <- coef_estimates - 1.96 * std_errors
> ci_upper <- coef_estimates + 1.96 * std_errors
> ci_mod2 <- cbind(coef_estimates, ci_lower, ci_upper)
```

```
> colnames(ci_mod2) <- c("Estimate", "CI Lower (95%)", "CI Upper (95%)")

> ci_mod2

                Estimate CI Lower (95%) CI Upper (95%)

(Intercept)     172.091290    144.9914408     199.191139

Time            1.487039      0.7331034       2.240974

Intervention    -4.737115    -30.3958933      20.921663

Post            6.618193      5.2087561       8.027630

factor(Month)2  -11.851477    -25.8535623      2.150609

factor(Month)3   17.234841      0.5146337     33.955048

factor(Month)4   -1.862261    -20.0769379     16.352415

factor(Month)5   -9.249523    -28.2605916      9.761546

factor(Month)6    7.143642    -12.2426118     26.529896

factor(Month)7   24.383333      4.9037677     43.862899

factor(Month)8   31.020666     11.6817700     50.359563

factor(Month)9   23.270367      4.3372604     42.203473

factor(Month)10  23.460295      5.3277450     41.592846

factor(Month)11  11.043742     -5.5870646     27.674549

factor(Month)12  18.486728      5.0787790     31.894678
```

##

Main ITS Analysis Results for the Number of Patients Prescribed Psychostimulants.

##

Residuals:

```
    Min      1Q  Median      3Q     Max
-37.102  -8.290  -0.014   7.744  31.665
```

Coefficients:

```
              Estimate Std. Error t value Pr(>|t|)
(Intercept)    239.2892     7.7560  30.852  < 2e-16 ***
Time             2.7052     0.1611  16.787  < 2e-16 ***
Intervention   -63.2827     6.9421  -9.116 2.38e-13 ***
Post             0.8267     0.3104   2.663  0.00968 **
factor(Month)2   0.7719     8.5368   0.090  0.92822
factor(Month)3  18.9981     8.5180   2.230  0.02908 *
factor(Month)4   0.2242     8.5018   0.026  0.97904
factor(Month)5  -7.5496     8.4882  -0.889  0.37696
factor(Month)6   8.5337     8.4772   1.007  0.31772
```

```
factor(Month)7    2.0456     8.4689   0.242  0.80987
factor(Month)8  -15.5854     8.4632  -1.842  0.06997 .
factor(Month)9   -6.2164     8.4601  -0.735  0.46504
factor(Month)10  18.8669     8.4597   2.230  0.02909 *
factor(Month)11  10.9503     8.4620   1.294  0.20009
factor(Month)12  15.1475     8.7582   1.730  0.08832 .
---
Signif. codes:  0 ‘***’ 0.001 ‘**’ 0.01 ‘*’ 0.05 ‘.’ 0.1 ‘ ’ 1

Residual standard error: 15.17 on 67 degrees of freedom
Multiple R-squared:  0.9259,     Adjusted R-squared:  0.9105
F-statistic: 59.83 on 14 and 67 DF,  p-value: < 2.2e-16

> confint(mod1, level = 0.95)
                   2.5 %     97.5 %
(Intercept)     223.8082585 254.770218
Time              2.3835904   3.026901
Intervention    -77.1392649 -49.426156
```

```
Post             0.2071315   1.446282
factor(Month)2  -16.2675209  17.811332
factor(Month)3    1.9961474  35.999995
factor(Month)4  -16.7453627  17.193836
factor(Month)5  -24.4920808   9.392885
factor(Month)6   -8.3868891  25.454310
factor(Month)7  -14.8583794  18.949560
factor(Month)8  -32.4779956   1.307222
factor(Month)9  -23.1028913  10.670163
factor(Month)10   1.9812134  35.752675
factor(Month)11  -5.9399678  27.840473
factor(Month)12  -2.3338594  32.628822
>
> durbinWatsonTest(mod1)
 lag Autocorrelation D-W Statistic p-value
   1       0.2963641      1.390764   0.004
Alternative hypothesis: rho != 0
> mod2 <- cochrane.orcutt(mod1)
```

```
> summary(mod2)

Call:

lm(formula = Outcome ~ Time + Intervention + Post + factor(Month),
    data = data)


                Estimate Std. Error t value  Pr(>|t|)
(Intercept)     242.30211    9.90878  24.453 < 2.2e-16 ***
Time              2.55444    0.25632   9.966 8.657e-15 ***
Intervention    -50.34523    9.95728  -5.056 3.634e-06 ***
Post              0.59991    0.48055   1.248   0.21630
factor(Month)2   -1.85098    7.22840  -0.256   0.79869
factor(Month)3   17.11833    8.17323   2.094   0.04006 *
factor(Month)4   -1.21438    8.56934  -0.142   0.88774
factor(Month)5   -8.66490    8.71158  -0.995   0.32354
factor(Month)6    7.69579    8.75136   0.879   0.38238
factor(Month)7    1.46714    8.74939   0.168   0.86734
factor(Month)8  -15.91136    8.72293  -1.824   0.07267 .
factor(Month)9   -6.29259    8.66063  -0.727   0.47006
```

```
factor(Month)10  19.03941    8.51029   2.237   0.02865 *
factor(Month)11  11.37099    8.12073   1.400   0.16612
factor(Month)12  14.46153    7.05193   2.051   0.04427 *
---
Signif. codes:  0 ‘***’ 0.001 ‘**’ 0.01 ‘*’ 0.05 ‘.’ 0.1 ‘ ’ 1

Residual standard error: 13.4566 on 76 degrees of freedom
Multiple R-squared:  0.8426 ,  Adjusted R-squared:  0.8344
F-statistic: 25.2 on 4 and 76 DF,  p-value: < 3.791e-21

Durbin-Watson statistic
(original):    1.39076 , p-value: 1.913e-03
(transformed): 2.09088 , p-value: 5.548e-01> coef_estimates <- summary(mod2)$coefficients[, 1]
> std_errors <- summary(mod2)$coefficients[, 2]
> ci_lower <- coef_estimates - 1.96 * std_errors
> ci_upper <- coef_estimates + 1.96 * std_errors
> ci_mod2 <- cbind(coef_estimates, ci_lower, ci_upper)
> colnames(ci_mod2) <- c("Estimate", "CI Lower (95%)", "CI Upper (95%)")
```

```
> ci_mod2
                 Estimate CI Lower (95%) CI Upper (95%)
(Intercept)     242.3021128    222.8808983     261.723327
Time             2.5544370      2.0520523       3.056822
Intervention    -50.3452297    -69.8614965     -30.828963
Post             0.5999055     -0.3419644       1.541776
factor(Month)2   -1.8509778    -16.0186448      12.316689
factor(Month)3   17.1183336      1.0987947      33.137873
factor(Month)4   -1.2143849    -18.0102825      15.581513
factor(Month)5   -8.6648971    -25.7395990       8.409805
factor(Month)6    7.6957932     -9.4568637      24.848450
factor(Month)7    1.4671378    -15.6816609      18.615936
factor(Month)8  -15.9113624    -33.0083110       1.185586
factor(Month)9   -6.2925880    -23.2674185      10.682243
factor(Month)10  19.0394094      2.3592349      35.719584
factor(Month)11  11.3709921     -4.5456402      27.287624
factor(Month)12  14.4615346      0.6397483      28.283321
```

Supplementary Figure 1. Interrupted Time Series Analysis with Counterfactual Scenario for Antidepressant Prescriptions in Children Aged 6-11 Years.

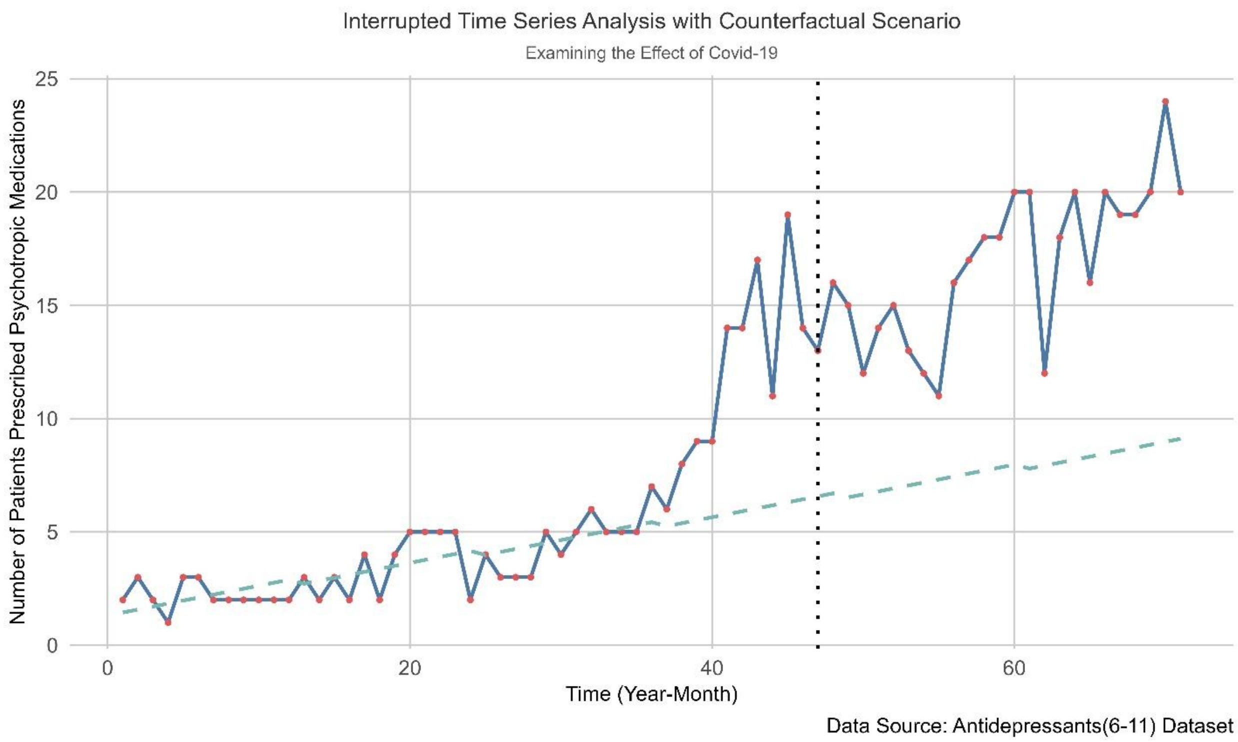

##

```
Residuals:
    Min      1Q  Median      3Q     Max 
-4.8617 -0.8563 -0.0869  0.9724  4.5166 

Coefficients:
                Estimate Std. Error t value Pr(>|t|)    
(Intercept)      2.52161    0.95648   2.636 0.010824 *  
Time             0.08851    0.02939   3.012 0.003893 ** 
Intervention     6.13927    0.91476   6.711 1.04e-08 ***
Post             0.18026    0.04367   4.128 0.000123 ***
factor(Month)2  -2.83848    1.08682  -2.612 0.011541 *  
factor(Month)3  -1.51712    1.08474  -1.399 0.167448    
factor(Month)4  -1.52909    1.08313  -1.412 0.163562    
factor(Month)5  -0.87440    1.08199  -0.808 0.422433    
factor(Month)6  -1.05304    1.08132  -0.974 0.334324    
factor(Month)7  -0.73167    1.08112  -0.677 0.501337    
factor(Month)8  -0.74365    1.08140  -0.688 0.494498    
factor(Month)9   0.57771    1.08214   0.534 0.595552    
factor(Month)10  0.39907    1.08336   0.368 0.713989    
factor(Month)11 -0.61290    1.08504  -0.565 0.574423    
factor(Month)12  0.08264    1.13505   0.073 0.942220    
---
Signif. codes:  0 ‘***’ 0.001 ‘**’ 0.01 ‘*’ 0.05 ‘.’ 0.1 ‘ ’ 1
```

```
Residual standard error: 1.868 on 56 degrees of freedom
Multiple R-squared:  0.9391,     Adjusted R-squared:  0.9238
F-statistic: 61.65 on 14 and 56 DF,  p-value: < 2.2e-16

> confint(mod1, level = 0.95)
                      2.5 %     97.5 %
(Intercept)      0.60555447  4.4376702
Time             0.02963887  0.1473827
Intervention     4.30679912  7.9717489
Post             0.09277443  0.2677417
factor(Month)2  -5.01563078 -0.6613208
factor(Month)3  -3.69011243  0.6558812
factor(Month)4  -3.69886274  0.6406853
factor(Month)5  -3.04188588  1.2930955
factor(Month)6  -3.21918480  1.1131148
factor(Month)7  -2.89742794  1.4340783
factor(Month)8  -2.90994914  1.4226533
factor(Month)9  -1.59008102  2.7455055
factor(Month)10 -1.77115497  2.5692999
factor(Month)11 -2.78650115  1.5606998
factor(Month)12 -2.19113074  2.3564080
###############################################################
```

Supplementary Figure 2. Interrupted Time Series Analysis with Counterfactual Scenario for Antidepressant Prescriptions in Adolescents Aged 12-17 Years.

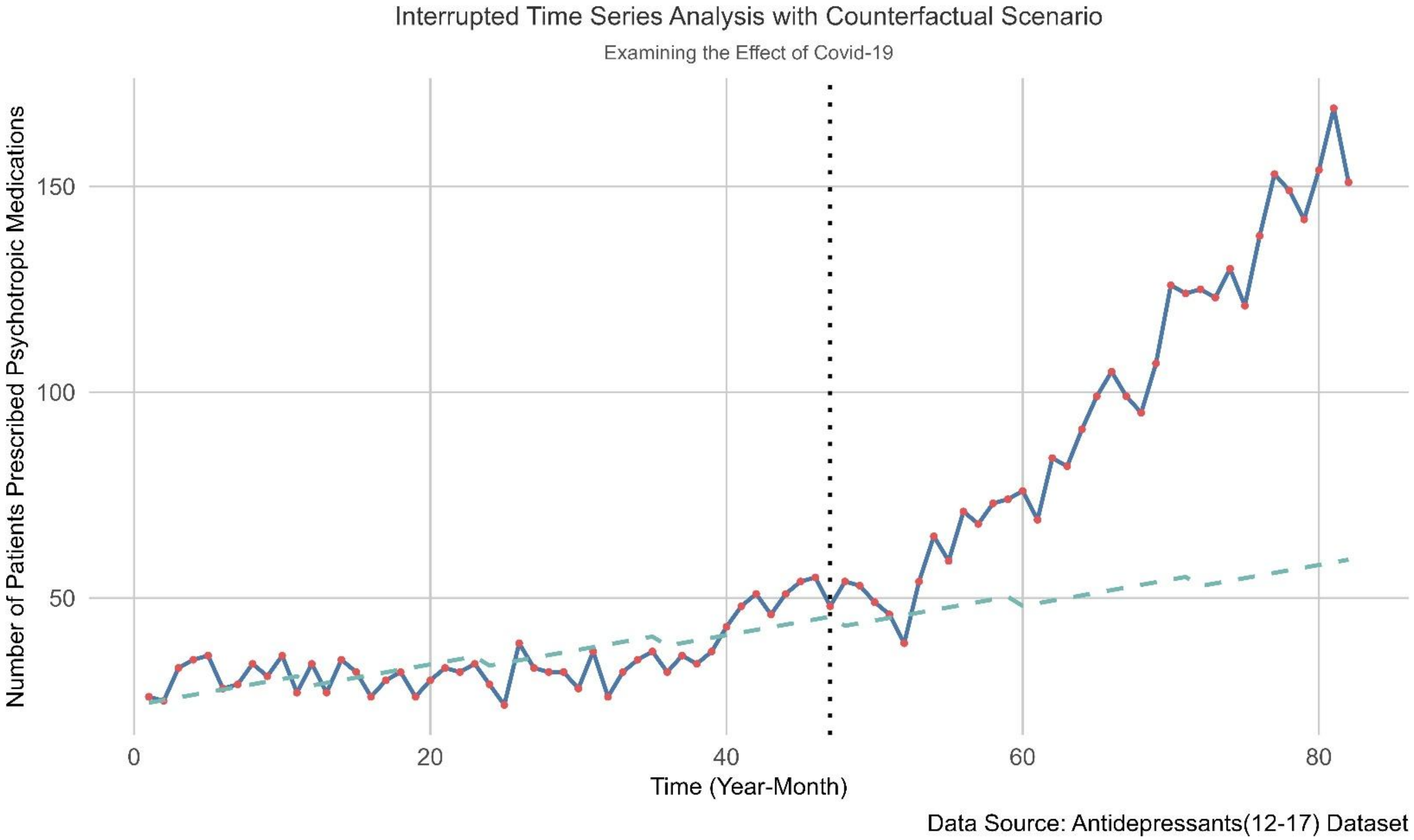

###

Residuals:

Min 1Q Median 3Q Max

```
-12.1109  -3.3502  -0.5387   3.8577  16.2948


Coefficients:

                Estimate Std. Error t value Pr(>|t|)

(Intercept)      25.34155    3.41788   7.414 2.73e-10 ***

Time              0.39465    0.07101   5.557 5.13e-07 ***

Intervention     -4.89113    3.05924  -1.599    0.115

Post              3.18473    0.13679  23.282  < 2e-16 ***

factor(Month)2   -3.08321    3.76196  -0.820    0.415

factor(Month)3    0.58583    3.75368   0.156    0.876

factor(Month)4   -2.88799    3.74654  -0.771    0.444

factor(Month)5   -1.79038    3.74055  -0.479    0.634

factor(Month)6    3.30723    3.73572   0.885    0.379

factor(Month)7    2.40484    3.73205   0.644    0.522

factor(Month)8   -2.21184    3.72954  -0.593    0.555

factor(Month)9   -0.68566    3.72820  -0.184    0.855

factor(Month)10   2.26909    3.72802   0.609    0.545

factor(Month)11   2.50956    3.72902   0.673    0.503

factor(Month)12   0.45623    3.85952   0.118    0.906
```

```
---
Signif. codes:  0 '***' 0.001 '**' 0.01 '*' 0.05 '.' 0.1 ' ' 1

Residual standard error: 6.684 on 67 degrees of freedom
Multiple R-squared:  0.9767,     Adjusted R-squared:  0.9719
F-statistic:   201 on 14 and 67 DF,  p-value: < 2.2e-16

> confint(mod1, level = 0.95)
                       2.5 %     97.5 %
(Intercept)      18.5194306 32.1636791
Time              0.2529054  0.5363983
Intervention    -10.9974052  1.2151469
Post              2.9116925  3.4577585
factor(Month)2  -10.5921026  4.4256913
factor(Month)3   -6.9065388  8.0782020
factor(Month)4  -10.3661141  4.5901375
factor(Month)5   -9.2565559  5.6757966
factor(Month)6   -4.1493038 10.7637617
factor(Month)7   -5.0443667  9.8540420
```

factor(Month)8   -9.6560372 5.2323584

factor(Month)9   -8.1271771 6.7558585

factor(Month)10 -5.1720746 9.7102591

factor(Month)11 -4.9335873 9.9527034

factor(Month)12 -7.2474119 8.1598659

##

Supplementary Figure 3. Interrupted Time Series Analysis with Counterfactual Scenario for Antidepressant Prescriptions in Young Adults Aged 18-22 Years.

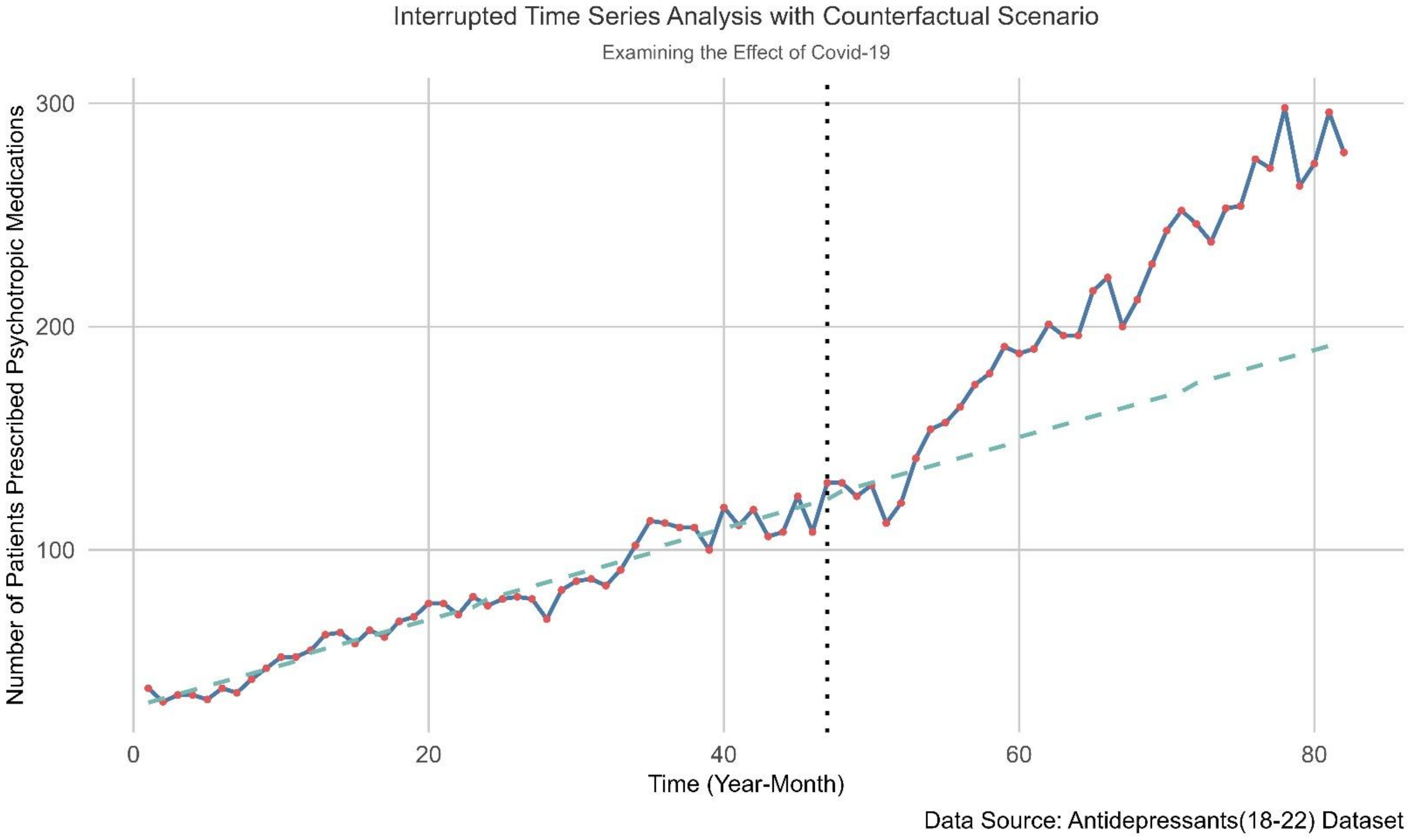

##

Residuals:

Min 1Q Median 3Q Max

```
-16.8695  -3.7059   0.1768   4.9794  17.4974


Coefficients:

                Estimate Std. Error t value Pr(>|t|)

(Intercept)      33.70728    3.93917   8.557 2.39e-12 ***

Time             1.97908    0.08185  24.181  < 2e-16 ***

Intervention     -2.27718    3.52583  -0.646  0.52058

Post             3.22302    0.15765  20.444  < 2e-16 ***

factor(Month)2   -2.53281    4.33572  -0.584  0.56107

factor(Month)3   -2.03604    4.32618  -0.471  0.63943

factor(Month)4  -10.25356    4.31795  -2.375  0.02044 *

factor(Month)5   -7.04250    4.31105  -1.634  0.10704

factor(Month)6   -5.26002    4.30548  -1.222  0.22610

factor(Month)7    1.23675    4.30125   0.288  0.77459

factor(Month)8  -11.40934    4.29836  -2.654  0.00992 **

factor(Month)9   -9.05543    4.29681  -2.107  0.03882 *

factor(Month)10  -1.41580    4.29661  -0.330  0.74279

factor(Month)11  -5.20474    4.29775  -1.211  0.23014

factor(Month)12   4.88675    4.44816   1.099  0.27587
```

Signif. codes: 0 '***' 0.001 '**' 0.01 '*' 0.05 '.' 0.1 ' ' 1

Residual standard error: 7.703 on 67 degrees of freedom

Multiple R-squared: 0.9916,   Adjusted R-squared: 0.9899

F-statistic: 568.2 on 14 and 67 DF, p-value: < 2.2e-16

```
> confint(mod1, level = 0.95)
                     2.5 %     97.5 %
(Intercept)      25.844664 41.5698903
Time              1.815714  2.1424448
Intervention     -9.314762  4.7604104
Post              2.908343  3.5376932
factor(Month)2  -11.186944  6.1213169
factor(Month)3  -10.671127  6.5990395
factor(Month)4  -18.872226 -1.6348936
factor(Month)5  -15.647398  1.5623898
factor(Month)6  -13.853800  3.3337595
factor(Month)7   -7.348584  9.8220831
```

factor(Month)8 -19.988902 -2.8297744

factor(Month)9 -17.631900 -0.4789506

factor(Month)10 -9.991869 7.1602718

factor(Month)11 -13.783094 3.3736075

factor(Month)12 -3.991822 13.7653262

##

Supplementary Figure 4. Interrupted Time Series Analysis with Counterfactual Scenario for Antipsychotic Prescriptions in Children Aged 6-11 Years.

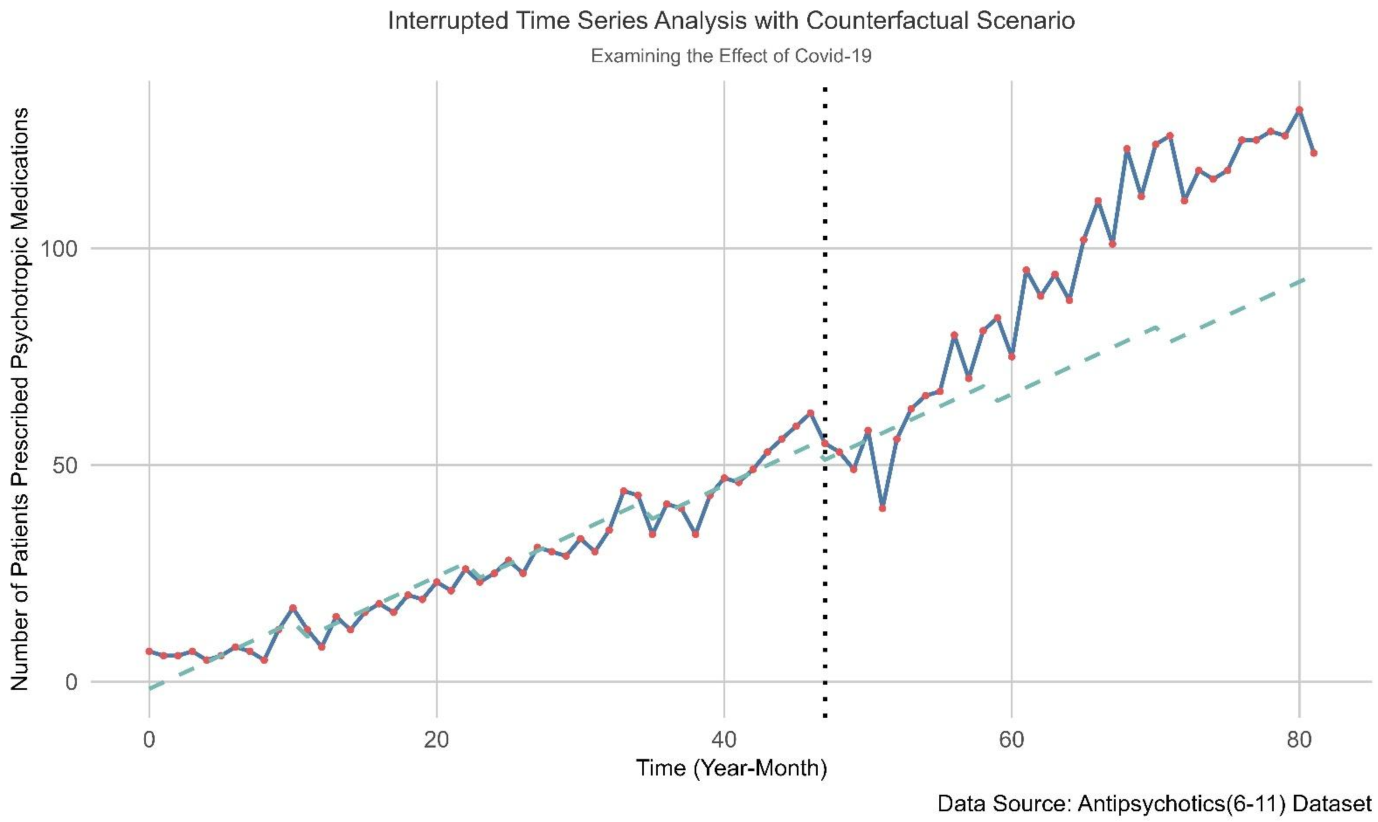

##

```
Residuals:
    Min       1Q   Median       3Q      Max
-16.7735  -2.8753  -0.5236   3.0051  15.4486

Coefficients:
               Estimate Std. Error t value Pr(>|t|)
(Intercept)      2.05690    2.96864   0.693    0.491
Time             1.11886    0.06243  17.922   <2e-16 ***
Intervention    -2.36583    2.68938  -0.880    0.382
Post             1.50443    0.12025  12.511   <2e-16 ***
factor(Month)2  -3.34473    3.30714  -1.011    0.315
factor(Month)3  -0.67977    3.29986  -0.206    0.837
factor(Month)4  -4.01481    3.29359  -1.219    0.227
factor(Month)5  -4.49272    3.28833  -1.366    0.176
factor(Month)6  -3.39919    3.28408  -1.035    0.304
factor(Month)7  -2.59137    3.28085  -0.790    0.432
factor(Month)8  -0.49785    3.27865  -0.152    0.880
factor(Month)9  -3.83289    3.27746  -1.169    0.246
factor(Month)10  1.68921    3.27731   0.515    0.608
factor(Month)11 -2.07441    3.27818  -0.633    0.529
factor(Month)12  4.78700    3.39291   1.411    0.163
---
Signif. codes:  0 ‘***’ 0.001 ‘**’ 0.01 ‘*’ 0.05 ‘.’ 0.1 ‘ ’ 1
```

```
Residual standard error: 5.876 on 67 degrees of freedom
Multiple R-squared:  0.9822,     Adjusted R-squared:  0.9785
F-statistic: 264.3 on 14 and 67 DF,  p-value: < 2.2e-16

> confint(mod1, level = 0.95)
                     2.5 %    97.5 %
(Intercept)      -3.8685203  7.982324
Time              0.9942512  1.243470
Intervention     -7.7338668  3.002202
Post              1.2644038  1.744451
factor(Month)2   -9.9458072  3.256353
factor(Month)3   -7.2663225  5.906781
factor(Month)4  -10.5888439  2.559215
factor(Month)5  -11.0562401  2.070809
factor(Month)6   -9.9542349  3.155859
factor(Month)7   -9.1399792  3.957230
factor(Month)8   -7.0420503  6.046356
factor(Month)9  -10.3747382  2.708956
factor(Month)10  -4.8523307  8.230747
factor(Month)11  -8.6176852  4.468871
factor(Month)12  -1.9852751 11.559281
################################################################
```

Supplementary Figure 5. Interrupted Time Series Analysis with Counterfactual Scenario for Antipsychotic Prescriptions in Adolescents Aged 12-17 Years.

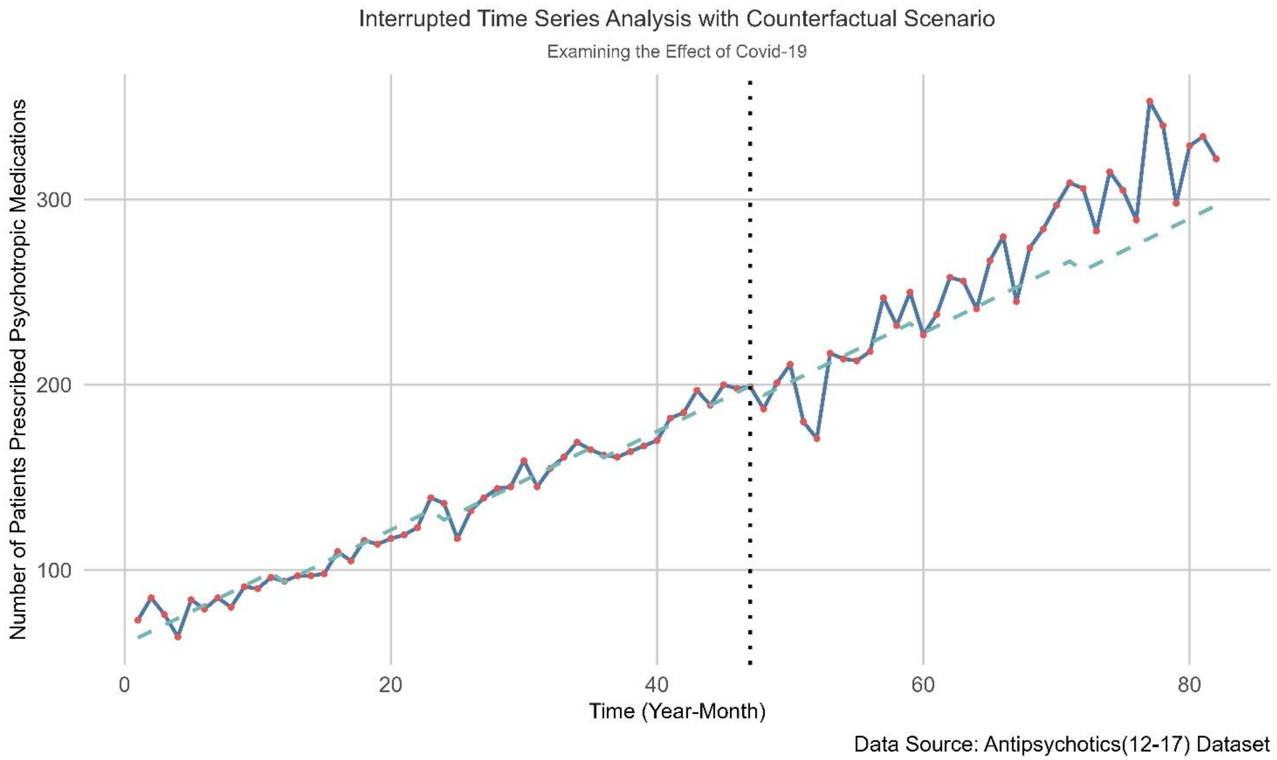

###

Residuals:

Min	1Q	Median	3Q	Max
-21.1337	-5.3235	-0.4255	5.8800	29.2691

Coefficients:

```
               Estimate Std. Error t value Pr(>|t|)
(Intercept)      61.7218     5.2638  11.726  < 2e-16 ***
Time              2.7940     0.1094  25.546  < 2e-16 ***
Intervention     -9.6500     4.7115  -2.048   0.0445 *
Post              1.6732     0.2107   7.943 3.06e-11 ***
factor(Month)2   -2.4251     5.7937  -0.419   0.6769
factor(Month)3    7.2067     5.7809   1.247   0.2169
factor(Month)4   -2.1615     5.7699  -0.375   0.7091
factor(Month)5  -10.2440     5.7607  -1.778   0.0799 .
factor(Month)6    9.6735     5.7533   1.681   0.0973 .
factor(Month)7    9.0196     5.7476   1.569   0.1213
factor(Month)8   -5.3486     5.7438  -0.931   0.3551
factor(Month)9    0.4260     5.7417   0.074   0.9411
factor(Month)10   7.4864     5.7414   1.304   0.1967
factor(Month)11   3.2610     5.7430   0.568   0.5720
factor(Month)12  11.0184     5.9439   1.854   0.0682 .
---
```

Signif. codes: 0 ‘***’ 0.001 ‘**’ 0.01 ‘*’ 0.05 ‘.’ 0.1 ‘ ’ 1

Residual standard error: 10.29 on 67 degrees of freedom

Multiple R-squared: 0.9858,   Adjusted R-squared: 0.9828

F-statistic: 331.4 on 14 and 67 DF, p-value: < 2.2e-16

```
> confint(mod1, level = 0.95)
                    2.5 %     97.5 %
(Intercept)      51.2152021 72.2283311
Time              2.5756686  3.0122682
Intervention    -19.0541117 -0.2458986
Post              1.2527261  2.0937072
factor(Month)2  -13.9893833  9.1391050
factor(Month)3   -4.3321353 18.7454488
factor(Month)4  -13.6784017  9.3553069
factor(Month)5  -21.7424883  1.2544139
factor(Month)6   -1.8101266 21.1570724
factor(Month)7   -2.4527587 20.4918676
factor(Month)8  -16.8132523  6.1159530
```

factor(Month)9 -11.0344719 11.8864786

factor(Month)10 -3.9735642 18.9463055

factor(Month)11 -8.2019582 14.7240055

factor(Month)12 -0.8457869 22.8825348

##

Supplementary Figure 6. Interrupted Time Series Analysis with Counterfactual Scenario for Antipsychotic Prescriptions in Young Adults Aged 18-22 Years.

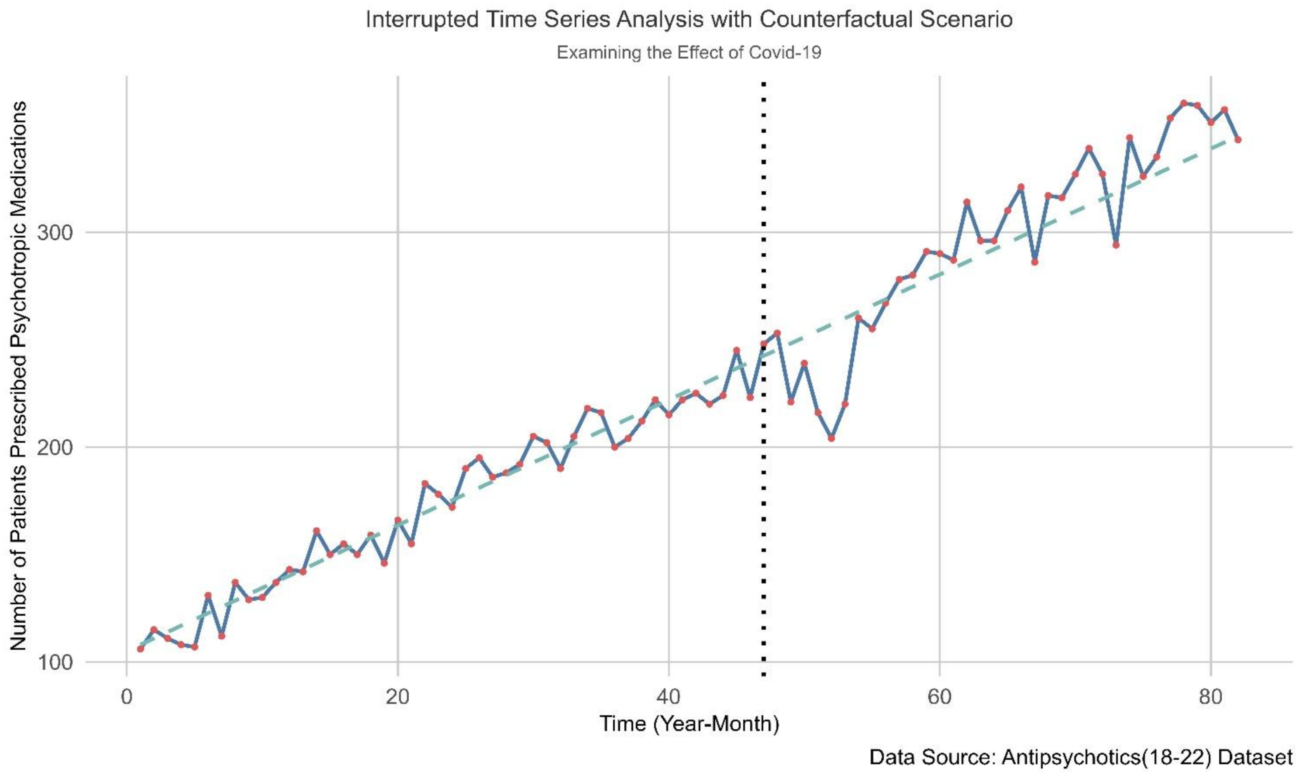

##

Residuals:

Min	1Q	Median	3Q	Max
-31.881	-6.759	0.716	7.948	18.824

Coefficients:

```
                 Estimate Std. Error t value Pr(>|t|)
(Intercept)     107.8176     5.9441  18.139  < 2e-16 ***
Time              2.8877     0.1235  23.382  < 2e-16 ***
Intervention    -15.7195     5.3203  -2.955  0.00432 **
Post              1.2302     0.2379   5.171 2.28e-06 ***
factor(Month)2   -7.9675     6.5424  -1.218  0.22756
factor(Month)3    8.0461     6.5280   1.233  0.22205
factor(Month)4   -5.7974     6.5156  -0.890  0.37677
factor(Month)5  -10.0695     6.5052  -1.548  0.12635
factor(Month)6   -5.9131     6.4968  -0.910  0.36600
factor(Month)7    5.9577     6.4904   0.918  0.36195
factor(Month)8   -9.0287     6.4861  -1.392  0.16852
factor(Month)9   -2.1580     6.4837  -0.333  0.74030
factor(Month)10  -0.8586     6.4834  -0.132  0.89503
factor(Month)11  -1.5593     6.4851  -0.240  0.81072
factor(Month)12   7.2978     6.7121   1.087  0.28082
---
```

```
Signif. codes:  0 ‘***’ 0.001 ‘**’ 0.01 ‘*’ 0.05 ‘.’ 0.1 ‘ ’ 1

Residual standard error: 11.62 on 67 degrees of freedom
Multiple R-squared:  0.9797,     Adjusted R-squared:  0.9754
F-statistic: 230.9 on 14 and 67 DF,  p-value: < 2.2e-16

> confint(mod1, level = 0.95)
                      2.5 %     97.5 %
(Intercept)      95.9531928 119.681942
Time              2.6412091   3.134232
Intervention    -26.3389044  -5.100022
Post              0.7553999   1.705065
factor(Month)2  -21.0262590   5.091227
factor(Month)3   -4.9839092  21.076093
factor(Month)4  -18.8026709   7.207786
factor(Month)5  -23.0539954   2.914898
factor(Month)6  -18.8807590   7.054593
factor(Month)7   -6.9972630  18.912599
factor(Month)8  -21.9749477   3.917501
```

factor(Month)9 -15.0995358 10.783591

factor(Month)10 -13.7996029 12.082303

factor(Month)11 -14.5037212 11.385067

factor(Month)12 -6.0996208 20.695217

##

Supplementary Figure 7. Interrupted Time Series Analysis with Counterfactual Scenario for Anxiolytic Prescriptions in Children Aged 6-11 Years.

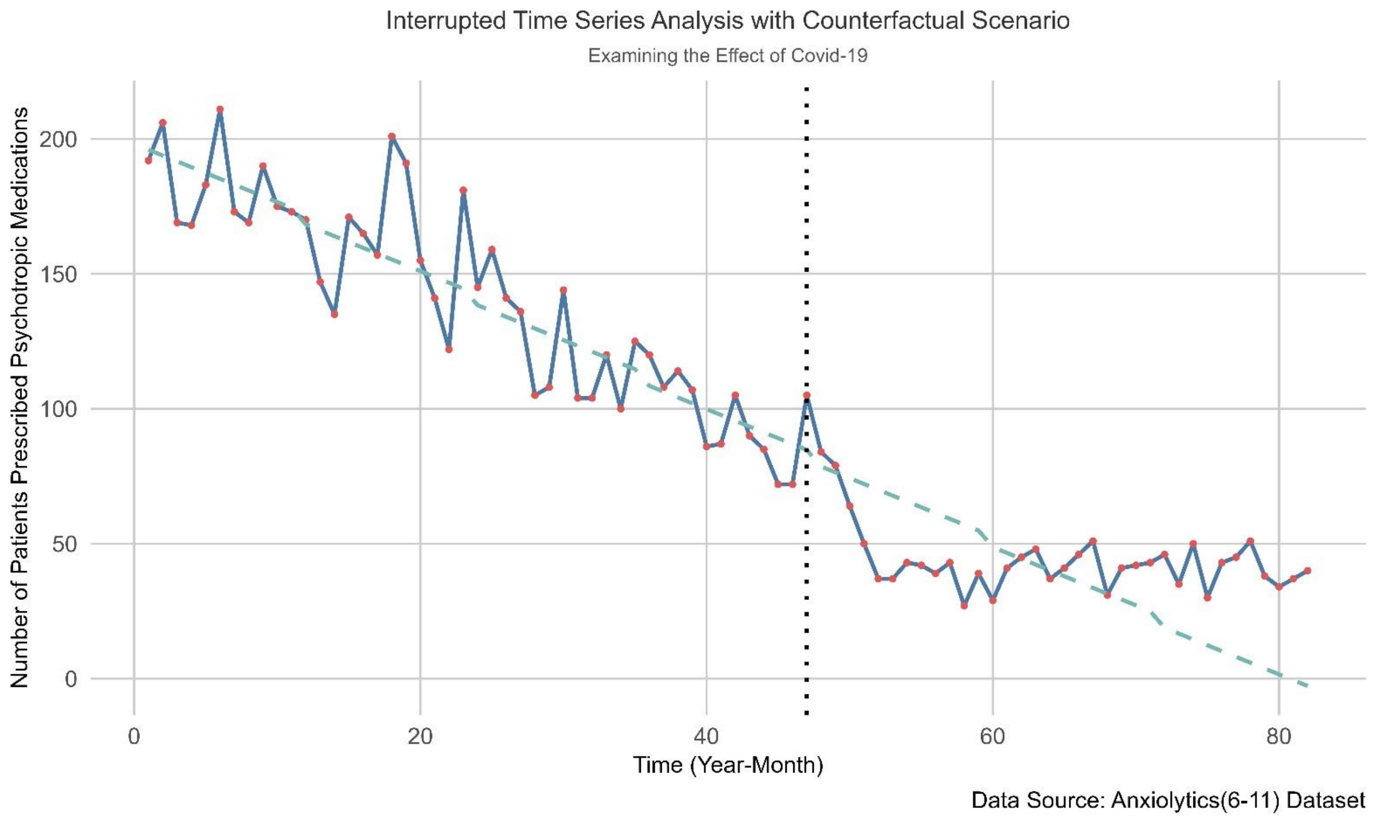

###

Residuals:

Min 1Q Median 3Q Max

-33.407 -7.451 0.733 5.942 36.853

Coefficients:

Estimate Std. Error t value Pr(>|t|)

(Intercept) 201.0429 6.8238 29.462 < 2e-16 ***

Time -2.4838 0.1418 -17.518 < 2e-16 ***

Intervention -31.4979 6.1078 -5.157 2.41e-06 ***

Post 2.2544 0.2731 8.255 8.36e-12 ***

factor(Month)2 1.4761 7.5108 0.197 0.8448

factor(Month)3 2.1366 7.4942 0.285 0.7765

factor(Month)4 -2.6315 7.4800 -0.352 0.7261

factor(Month)5 -11.1139 7.4680 -1.488 0.1414

factor(Month)6 -7.1677 7.4584 -0.961 0.3400

factor(Month)7 14.7784 7.4511 1.983 0.0514 .

factor(Month)8 0.2960 7.4460 0.040 0.9684

factor(Month)9 -8.4721 7.4434 -1.138 0.2591

factor(Month)10 -3.0973 7.4430 -0.416 0.6786

factor(Month)11 -11.0083 7.4450 -1.479 0.1439

factor(Month)12 10.2677 7.7056 1.333 0.1872

Signif. codes: 0 ‘***’ 0.001 ‘**’ 0.01 ‘*’ 0.05 ‘.’ 0.1 ‘ ’ 1

Residual standard error: 13.34 on 67 degrees of freedom

Multiple R-squared: 0.9539,   Adjusted R-squared: 0.9443

F-statistic: 99.08 on 14 and 67 DF, p-value: < 2.2e-16

```
> confint(mod1, level = 0.95)
                       2.5 %     97.5 %
(Intercept)     187.42246308 214.663254
Time             -2.76676707  -2.200772
Intervention    -43.68914457 -19.306740
Post              1.70926261   2.799485
factor(Month)2  -13.51543633  16.467643
factor(Month)3  -12.82197428  17.095115
factor(Month)4  -17.56163965  12.298570
factor(Month)5  -26.02017282   3.792322
factor(Month)6  -22.05473865   7.719250
factor(Month)7   -0.09392648  29.650800
```

```
factor(Month)8  -14.56632136  15.158414

factor(Month)9  -23.32907553   6.384958

factor(Month)10 -17.95362256  11.759010

factor(Month)11 -25.86853454   3.851998

factor(Month)12  -5.11265459  25.648031

################################################################
```

Supplementary Figure 8. Interrupted Time Series Analysis with Counterfactual Scenario for Anxiolytic Prescriptions in Adolescents Aged 12-17 Years.

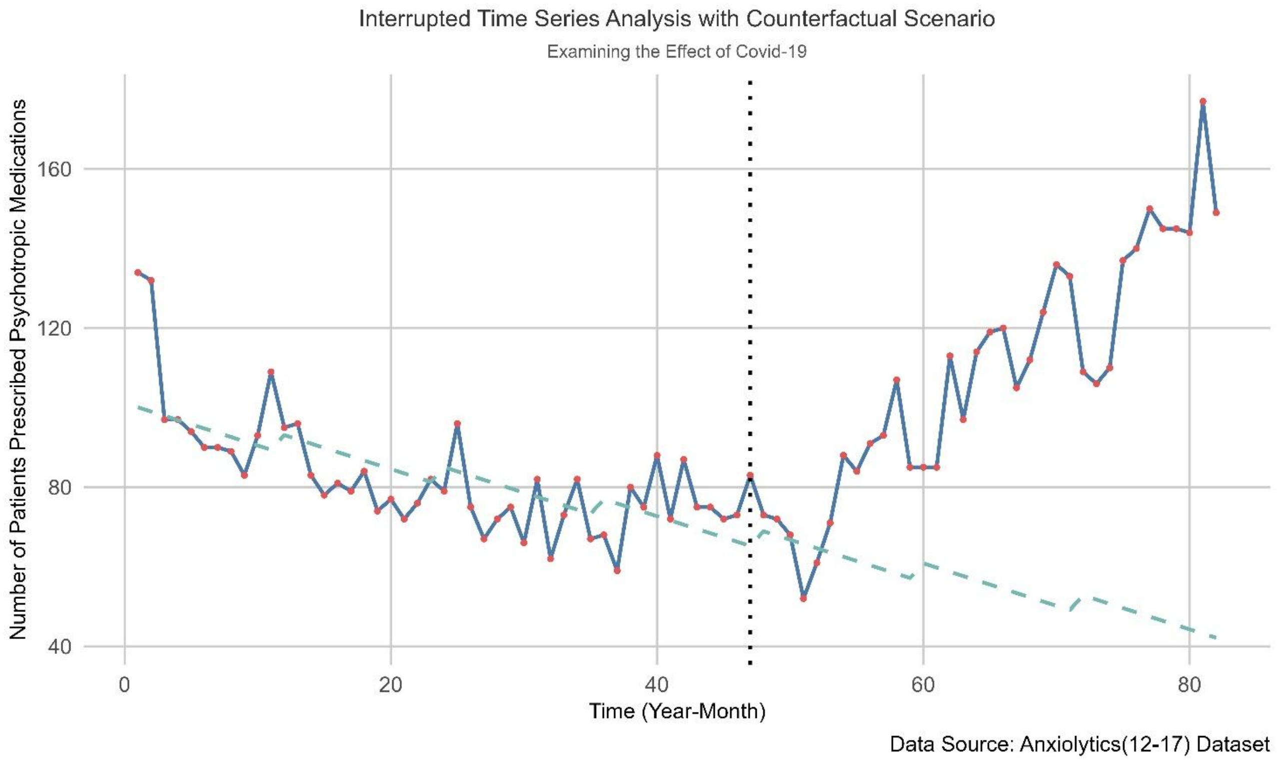

##

Residuals:

Min	1Q	Median	3Q	Max
-25.538	-7.454	-0.181	7.418	33.779

Coefficients:

	Estimate	Std. Error	t value	Pr(>|t|)	
(Intercept)	94.7351	6.0988	15.533	< 2e-16	***
Time	-0.6881	0.1267	-5.431	8.41e-07	***
Intervention	-0.9529	5.4588	-0.175	0.862	
Post	3.4090	0.2441	13.967	< 2e-16	***
factor(Month)2	6.1738	6.7127	0.920	0.361	
factor(Month)3	7.2581	6.6979	1.084	0.282	
factor(Month)4	-1.8005	6.6852	-0.269	0.789	
factor(Month)5	4.5695	6.6745	0.685	0.496	
factor(Month)6	4.7967	6.6659	0.720	0.474	
factor(Month)7	6.8810	6.6594	1.033	0.305	
factor(Month)8	2.5367	6.6549	0.381	0.704	
factor(Month)9	1.0495	6.6525	0.158	0.875	
factor(Month)10	6.5624	6.6522	0.987	0.327	
factor(Month)11	8.9324	6.6539	1.342	0.184	
factor(Month)12	8.7815	6.8868	1.275	0.207	

Signif. codes: 0 ‘***’ 0.001 ‘**’ 0.01 ‘*’ 0.05 ‘.’ 0.1 ‘ ’ 1

Residual standard error: 11.93 on 67 degrees of freedom

Multiple R-squared: 0.8228,   Adjusted R-squared: 0.7858

F-statistic: 22.22 on 14 and 67 DF, p-value: < 2.2e-16

```
> confint(mod1, level = 0.95)
                      2.5 %      97.5 %
(Intercept)      82.5619054 106.9082689
Time             -0.9410645  -0.4352087
Intervention    -11.8487265   9.9429635
Post              2.9217986   3.8961814
factor(Month)2   -7.2248128  19.5724616
factor(Month)3   -6.1110396  20.6272558
factor(Month)4  -15.1441954  11.5432647
factor(Month)5   -8.7528749  17.8919403
factor(Month)6   -8.5085265  18.1018737
factor(Month)7   -6.4111662  20.1730808
factor(Month)8  -10.7465203  15.8198596
```

factor(Month)9 -12.2288831 14.3279327

factor(Month)10 -6.7154017 19.8401616

factor(Month)11 -4.3489341 22.2136900

factor(Month)12 -4.9646018 22.5276553

##

Supplementary Figure 9. Interrupted Time Series Analysis with Counterfactual Scenario for Anxiolytic Prescriptions in Young Adults Aged 18-22 Years.

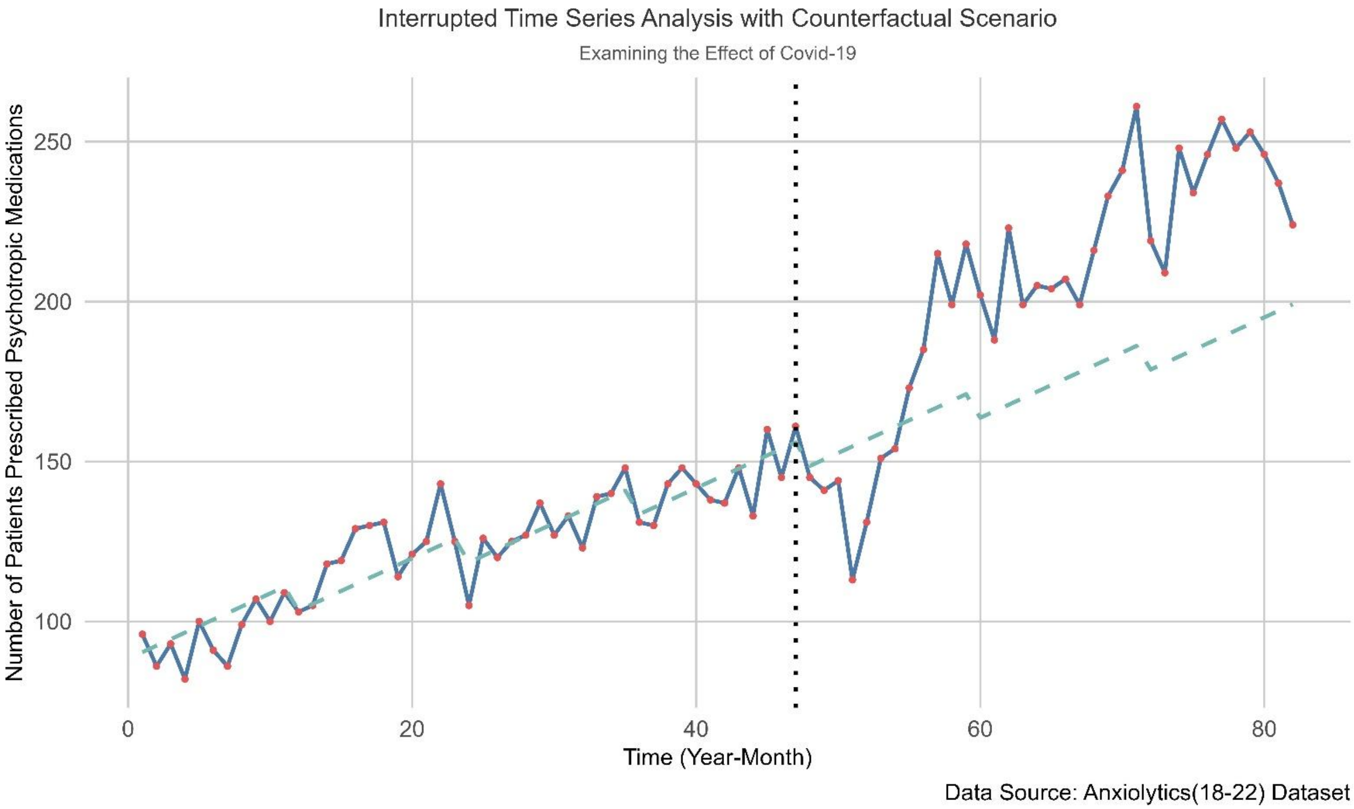

##

Residuals:

    Min   1Q Median   3Q   Max

-39.892 -6.438 1.264 6.528 28.535

Coefficients:

	Estimate	Std. Error	t value	Pr(>|t|)	
(Intercept)	87.7150	7.1246	12.312	< 2e-16	***
Time	1.2337	0.1480	8.334	6.02e-12	***
Intervention	-1.5103	6.3770	-0.237	0.81351	
Post	2.0836	0.2851	7.307	4.26e-10	***
factor(Month)2	-1.2868	7.8419	-0.164	0.87015	
factor(Month)3	9.0151	7.8246	1.152	0.25335	
factor(Month)4	-0.3973	7.8097	-0.051	0.95958	
factor(Month)5	2.0475	7.7972	0.263	0.79367	
factor(Month)6	7.6351	7.7872	0.980	0.33038	
factor(Month)7	2.3656	7.7795	0.304	0.76201	
factor(Month)8	1.8104	7.7743	0.233	0.81657	
factor(Month)9	2.1123	7.7715	0.272	0.78661	
factor(Month)10	13.2713	7.7711	1.708	0.09231	.
factor(Month)11	7.7161	7.7732	0.993	0.32445	
factor(Month)12	21.4282	8.0452	2.663	0.00968	**

```
---
Signif. codes:  0 '***' 0.001 '**' 0.01 '*' 0.05 '.' 0.1 ' ' 1

Residual standard error: 13.93 on 67 degrees of freedom
Multiple R-squared:  0.9368,     Adjusted R-squared:  0.9236
F-statistic: 70.98 on 14 and 67 DF,  p-value: < 2.2e-16

> confint(mod1, level = 0.95)
                      2.5 %     97.5 %
(Intercept)      73.4941687 101.935830
Time              0.9382088   1.529154
Intervention    -14.2389472  11.218319
Post              1.5144715   2.652755
factor(Month)2  -16.9392317  14.365608
factor(Month)3   -6.6028691  24.633070
factor(Month)4  -15.9855490  15.191004
factor(Month)5  -13.5158702  17.610865
factor(Month)6   -7.9081413  23.178390
factor(Month)7  -13.1623810  17.893598
```

```
factor(Month)8  -13.7071749  17.327931

factor(Month)9  -13.3996758  17.624257

factor(Month)10  -2.2398888  28.782581

factor(Month)11  -7.7992432  23.231475

factor(Month)12   5.3698569  37.486582

##############################################################
```

Supplementary Figure 10. Interrupted Time Series Analysis with Counterfactual Scenarios for Hypnotic and Sedative Prescriptions in Children Aged 6-11 Years.

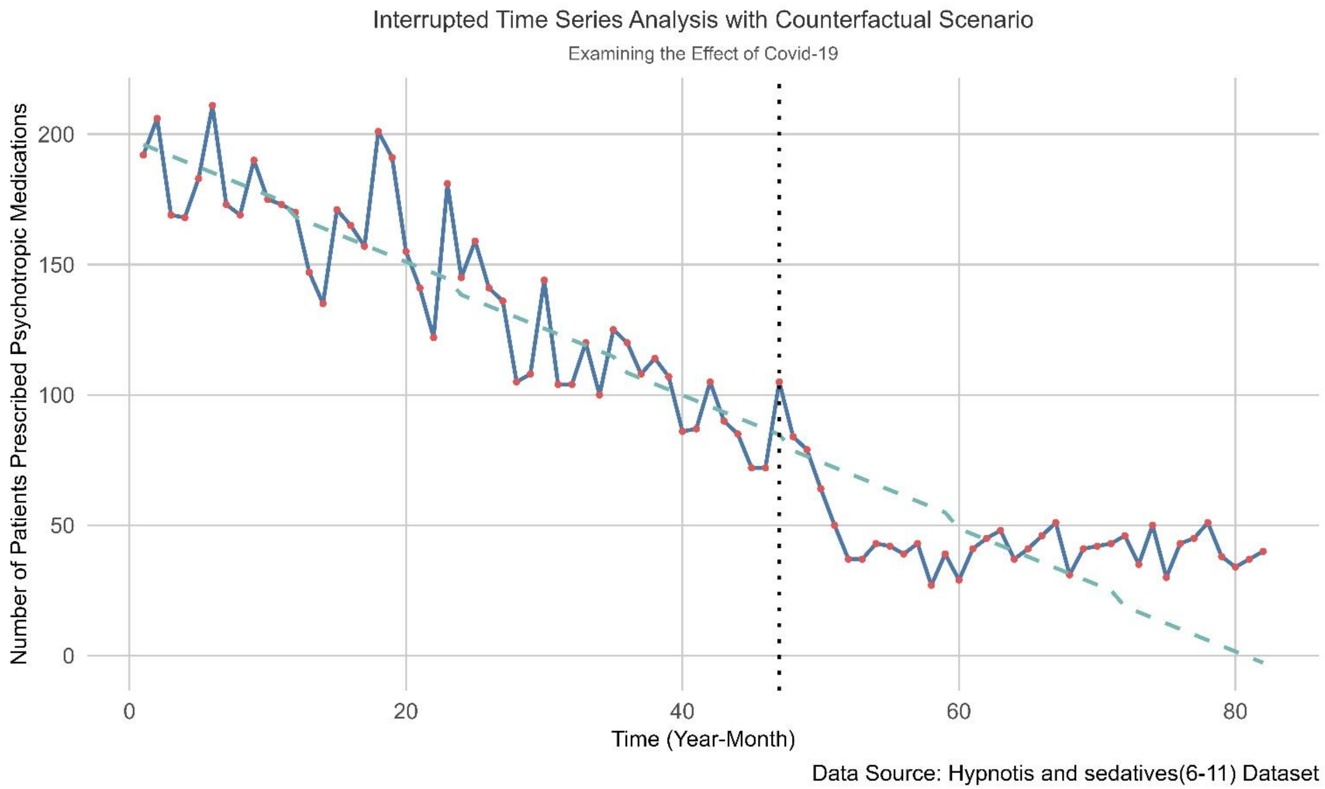

##

	Estimate	Std. Error	t value	Pr(>|t|)
(Intercept)	201.0429	6.8238	29.462	< 2e-16 ***
Time	-2.4838	0.1418	-17.518	< 2e-16 ***

Intervention	-31.4979	6.1078	-5.157	2.41e-06	***
Post	2.2544	0.2731	8.255	8.36e-12	***
factor(Month)2	1.4761	7.5108	0.197	0.8448	
factor(Month)3	2.1366	7.4942	0.285	0.7765	
factor(Month)4	-2.6315	7.4800	-0.352	0.7261	
factor(Month)5	-11.1139	7.4680	-1.488	0.1414	
factor(Month)6	-7.1677	7.4584	-0.961	0.3400	
factor(Month)7	14.7784	7.4511	1.983	0.0514	.
factor(Month)8	0.2960	7.4460	0.040	0.9684	
factor(Month)9	-8.4721	7.4434	-1.138	0.2591	
factor(Month)10	-3.0973	7.4430	-0.416	0.6786	
factor(Month)11	-11.0083	7.4450	-1.479	0.1439	
factor(Month)12	10.2677	7.7056	1.333	0.1872	

Signif. codes: 0 ‘***’ 0.001 ‘**’ 0.01 ‘*’ 0.05 ‘.’ 0.1 ‘ ’ 1

Residual standard error: 13.34 on 67 degrees of freedom

Multiple R-squared: 0.9539,   Adjusted R-squared: 0.9443

F-statistic: 99.08 on 14 and 67 DF, p-value: < 2.2e-16

```
> confint(mod1, level = 0.95)
                      2.5 %     97.5 %
(Intercept)     187.42246308 214.663254
Time             -2.76676707  -2.200772
Intervention    -43.68914457 -19.306740
Post              1.70926261   2.799485
factor(Month)2  -13.51543633  16.467643
factor(Month)3  -12.82197428  17.095115
factor(Month)4  -17.56163965  12.298570
factor(Month)5  -26.02017282   3.792322
factor(Month)6  -22.05473865   7.719250
factor(Month)7   -0.09392648  29.650800
factor(Month)8  -14.56632136  15.158414
factor(Month)9  -23.32907553   6.384958
factor(Month)10 -17.95362256  11.759010
factor(Month)11 -25.86853454   3.851998
factor(Month)12  -5.11265459  25.648031
##############################################################
```

Supplementary Figure 11. Interrupted Time Series Analysis with Counterfactual Scenarios for Hypnotic and Sedative Prescriptions in Adolescents Aged 12-17 Years.

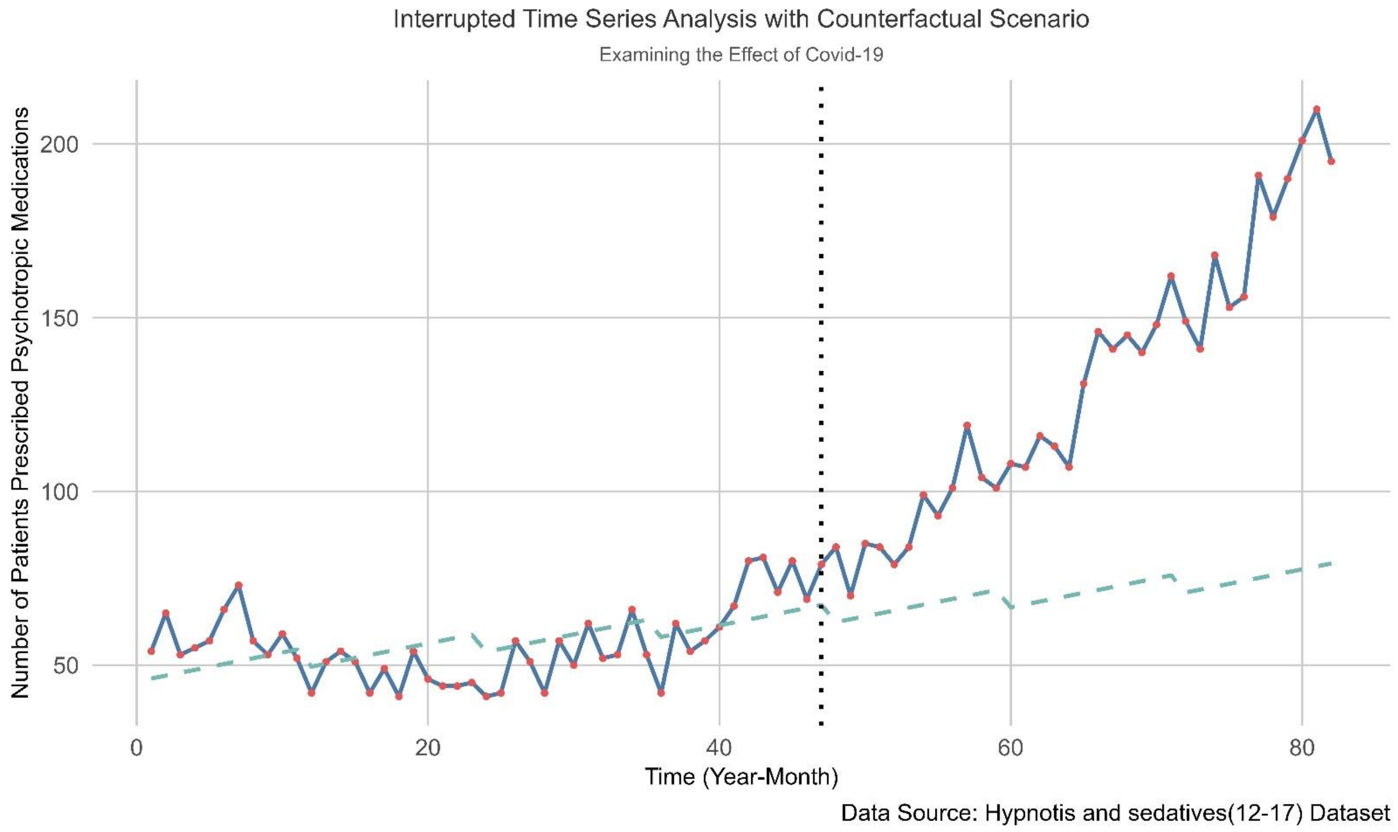

##

Estimate Std. Error t value Pr(>|t|)

(Intercept)	41.6939	5.0470	8.261	8.14e-12	***
Time	0.3564	0.1049	3.398	0.00115	**
Intervention	6.3373	4.5174	1.403	0.16528	
Post	3.3341	0.2020	16.506	< 2e-16	***
factor(Month)2	0.5437	5.5551	0.098	0.92232	
factor(Month)3	9.0442	5.5429	1.632	0.10744	
factor(Month)4	1.9732	5.5323	0.357	0.72246	
factor(Month)5	-2.6692	5.5235	-0.483	0.63050	
factor(Month)6	8.9742	5.5164	1.627	0.10847	
factor(Month)7	10.7603	5.5109	1.953	0.05505	.
factor(Month)8	13.6894	5.5072	2.486	0.01543	*
factor(Month)9	8.9041	5.5053	1.617	0.11049	
factor(Month)10	10.8332	5.5050	1.968	0.05322	.
factor(Month)11	7.0479	5.5065	1.280	0.20498	
factor(Month)12	5.8011	5.6992	1.018	0.31240	

Signif. codes: 0 ‘***’ 0.001 ‘**’ 0.01 ‘*’ 0.05 ‘.’ 0.1 ‘ ’ 1

Residual standard error: 9.87 on 67 degrees of freedom

Multiple R-squared: 0.9619, Adjusted R-squared: 0.954

F-statistic: 120.9 on 14 and 67 DF, p-value: < 2.2e-16

```
> confint(mod1, level = 0.95)
                   2.5 %     97.5 %
(Intercept)      31.6199613 51.7678023
Time              0.1470542  0.5656753
Intervention     -2.6795984 15.3541217
Post              2.9308911  3.7372418
factor(Month)2  -10.5443079 11.6317851
factor(Month)3   -2.0194400 20.1078449
factor(Month)4   -9.0693703 13.0158458
factor(Month)5  -13.6941182  8.3558072
factor(Month)6   -2.0365571 19.9848881
factor(Month)7   -0.2395574 21.7602447
factor(Month)8    2.6968709 24.6818871
factor(Month)9   -2.0844221 19.8926793
factor(Month)10  -0.1548686 21.8211964
factor(Month)11  -3.9430406 18.0388675
```

factor(Month)12 -5.5745595 17.1766667

##

Supplementary Figure 12. Interrupted Time Series Analysis with Counterfactual Scenario for Hypnotic and Sedative Prescriptions in Young Adults Aged 18-22 Years.

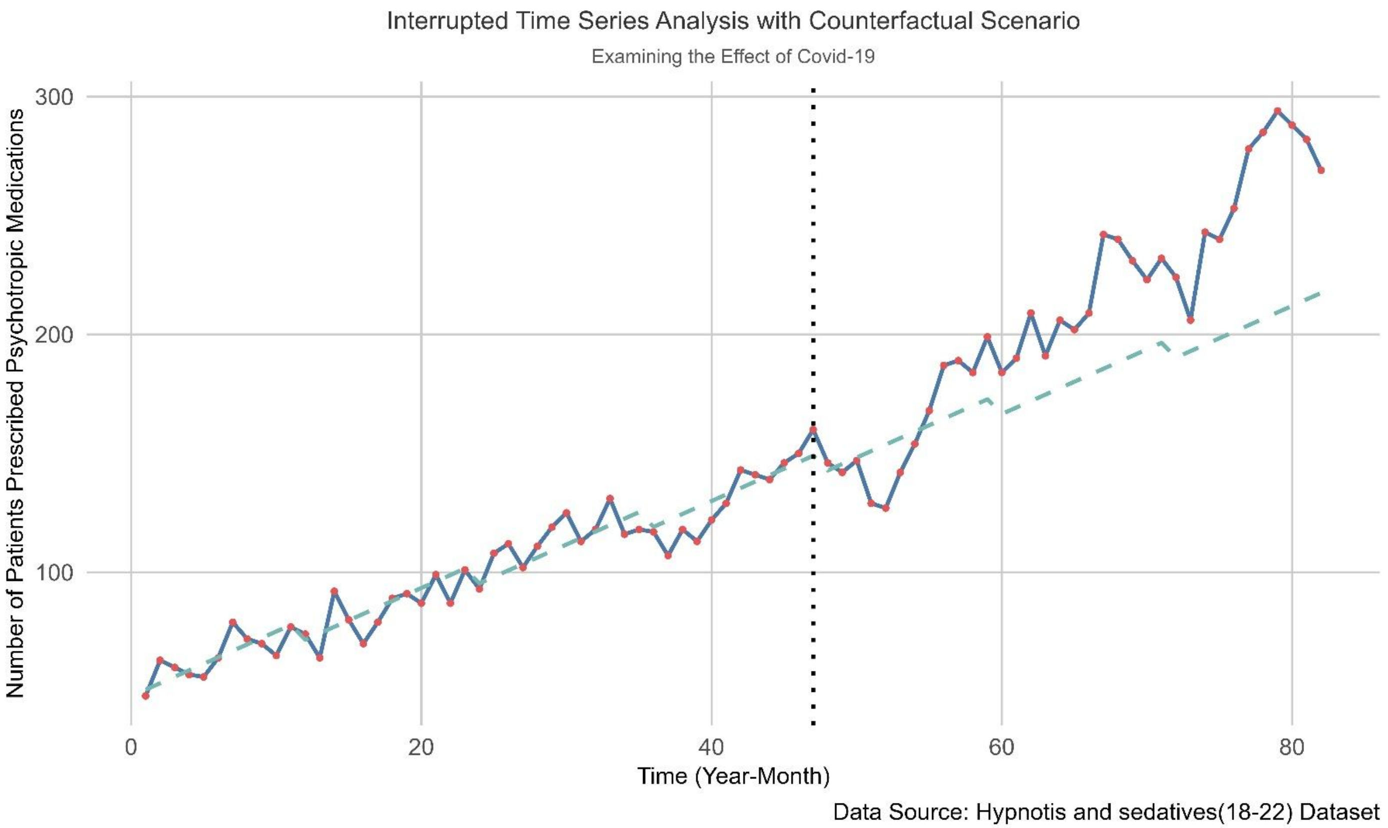

###

Estimate Std. Error t value Pr(>|t|)

(Intercept) 46.6699 4.9296 9.467 5.61e-14 ***

```
Time             1.9619     0.1024  19.154  < 2e-16 ***
Intervention   -10.3006     4.4124  -2.334  0.02258 *
Post             2.4759     0.1973  12.550  < 2e-16 ***
factor(Month)2   -4.0078     5.4259  -0.739  0.46271
factor(Month)3    9.9692     5.4140   1.841  0.06999 .
factor(Month)4   -2.9110     5.4037  -0.539  0.59188
factor(Month)5   -1.5054     5.3950  -0.279  0.78107
factor(Month)6    3.9001     5.3881   0.724  0.47168
factor(Month)7   10.0200     5.3828   1.861  0.06706 .
factor(Month)8   15.4255     5.3792   2.868  0.00552 **
factor(Month)9   12.8311     5.3772   2.386  0.01986 *
factor(Month)10  12.2366     5.3770   2.276  0.02606 *
factor(Month)11   1.4993     5.3784   0.279  0.78129
factor(Month)12  10.9539     5.5666   1.968  0.05323 .
---
Signif. codes:  0 ‘***’ 0.001 ‘**’ 0.01 ‘*’ 0.05 ‘.’ 0.1 ‘ ’ 1

Residual standard error: 9.64 on 67 degrees of freedom
Multiple R-squared:  0.9827,     Adjusted R-squared:  0.979
```

```
F-statistic: 271.1 on 14 and 67 DF,  p-value: < 2.2e-16


> confint(mod1, level = 0.95)
                      2.5 %    97.5 %
(Intercept)      36.8303367 56.509554
Time              1.7574601  2.166344
Intervention    -19.1077762 -1.493507
Post              2.0821506  2.869746
factor(Month)2  -14.8379348  6.822359
factor(Month)3   -0.8371214 20.775499
factor(Month)4  -13.6967421  7.874788
factor(Month)5  -12.2739588  9.263102
factor(Month)6   -6.8545015 14.654741
factor(Month)7   -0.7240975 20.764006
factor(Month)8    4.6886720 26.162333
factor(Month)9    2.0980858 23.564016
factor(Month)10   1.5041403 22.969058
factor(Month)11  -9.2360220 12.234603
factor(Month)12  -0.1571397 22.064910
```

###

Supplementary Figure 13. Interrupted Time Series Analysis with Counterfactual Scenarios for Psychostimulant Prescriptions in Children Aged 6-11 Years.

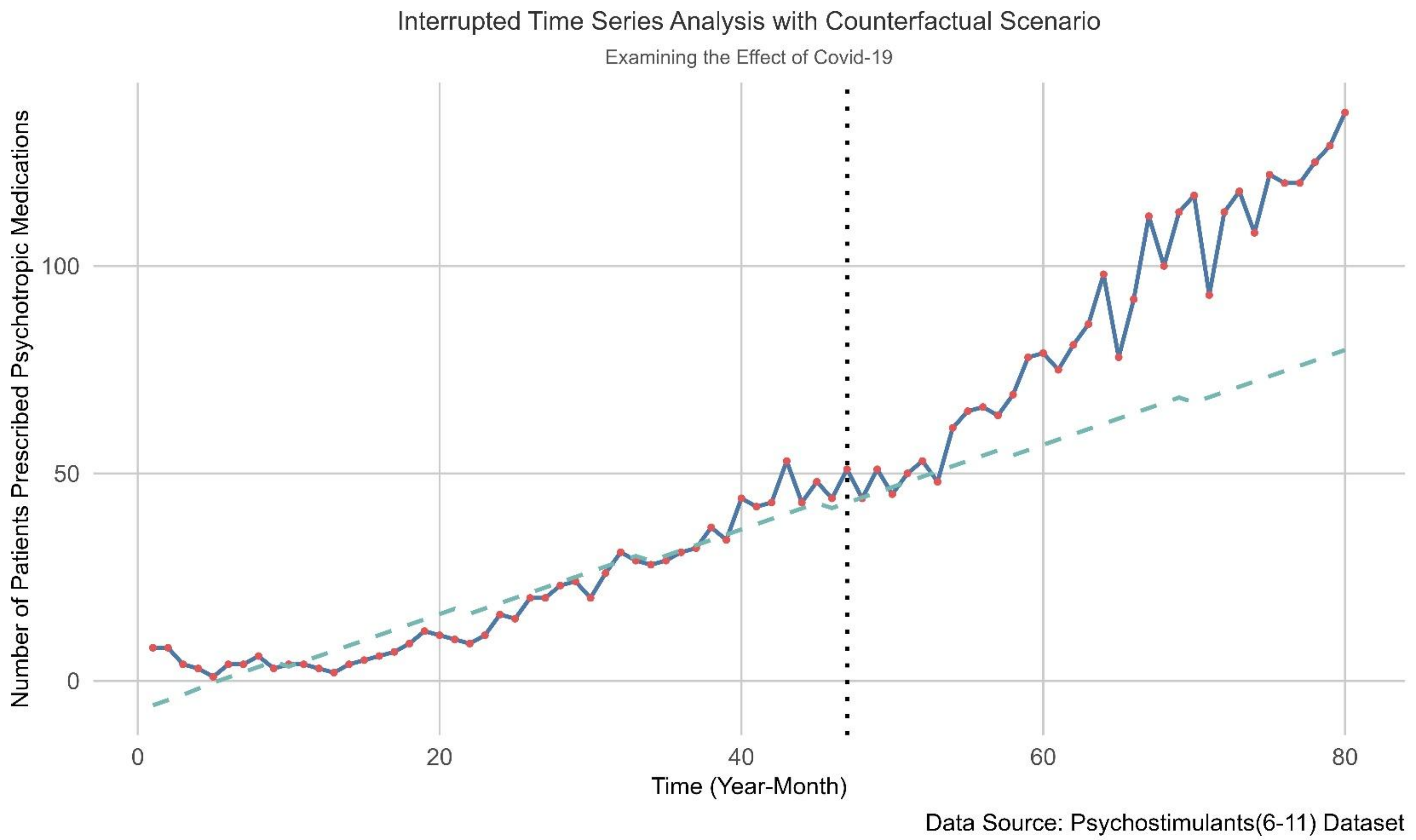

##

Estimate Std. Error t value Pr(>|t|)

(Intercept) -6.21504 3.07194 -2.023 0.0472 *

```
Time             1.06316    0.06801  15.633   <2e-16 ***

Intervention    -2.50134    2.76783  -0.904   0.3695

Post             1.70980    0.12499  13.680   <2e-16 ***

factor(Month)2   0.12209    3.37390   0.036   0.9712

factor(Month)3   1.18329    3.36616   0.352   0.7263

factor(Month)4  -0.18407    3.35950  -0.055   0.9565

factor(Month)5  -2.97041    3.47152  -0.856   0.3953

factor(Month)6  -0.33835    3.35028  -0.101   0.9199

factor(Month)7   1.36012    3.46838   0.392   0.6962

factor(Month)8  -3.77835    3.34500  -1.130   0.2628

factor(Month)9  -0.71715    3.34358  -0.214   0.8308

factor(Month)10  4.20120    3.34326   1.257   0.2134

factor(Month)11  1.40526    3.34404   0.420   0.6757

factor(Month)12  0.96643    3.46105   0.279   0.7810

---

Signif. codes:  0 ‘***’ 0.001 ‘**’ 0.01 ‘*’ 0.05 ‘.’ 0.1 ‘ ’ 1


Residual standard error: 5.994 on 65 degrees of freedom

Multiple R-squared:  0.9818,     Adjusted R-squared:  0.9779
```

F-statistic: 250.2 on 14 and 65 DF, p-value: < 2.2e-16

```
> confint(mod1, level = 0.95)
                      2.5 %      97.5 %
(Intercept)     -12.3501380 -0.07994282
Time              0.9273465  1.19898336
Intervention     -8.0290772  3.02639076
Post              1.4601877  1.95942076
factor(Month)2   -6.6160549  6.86023218
factor(Month)3   -5.5393944  7.90598109
factor(Month)4   -6.8934602  6.52531334
factor(Month)5   -9.9035219  3.96270129
factor(Month)6   -7.0293220  6.35261241
factor(Month)7   -5.5667173  8.28696186
factor(Month)8  -10.4587784  2.90207755
factor(Month)9   -7.3947348  5.96044332
factor(Month)10  -2.4757482 10.87815176
factor(Month)11  -5.2732477  8.08377492
factor(Month)12  -5.9457533  7.87861936
```

##

Supplementary Figure 14. Interrupted Time Series Analysis with Counterfactual Scenarios for Psychostimulant Prescriptions in Adolescents Aged 12-17 Years.

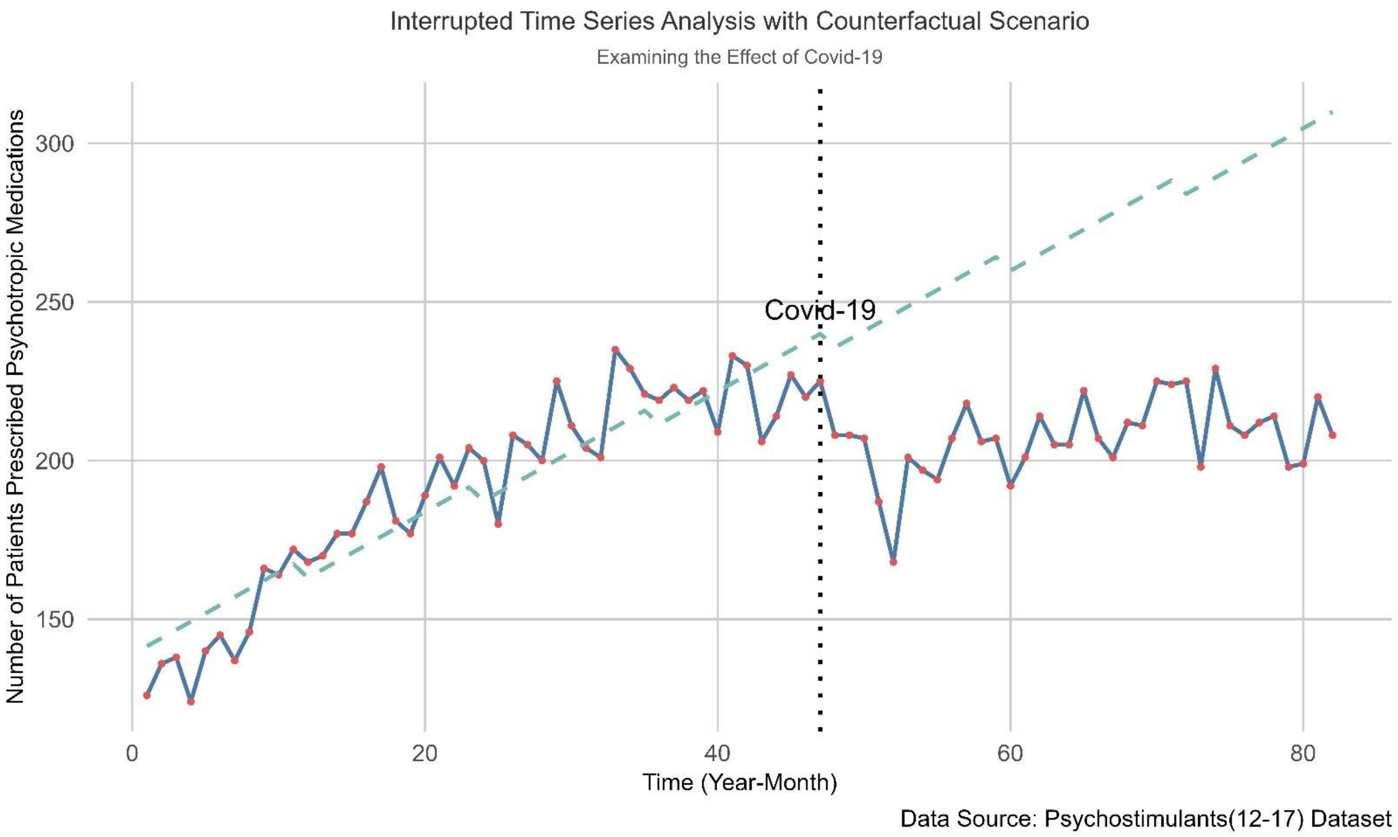

##

Estimate Std. Error t value Pr(>|t|)

(Intercept) 139.9457 5.8294 24.007 < 2e-16 ***

```
Time              2.0135     0.1211  16.624  < 2e-16 ***
Intervention    -41.8911     5.2177  -8.029 2.14e-11 ***
Post             -1.5087     0.2333  -6.467 1.35e-08 ***
factor(Month)2   -2.1617     6.4163  -0.337   0.7372
factor(Month)3    8.4714     6.4021   1.323   0.1903
factor(Month)4    0.6759     6.3900   0.106   0.9161
factor(Month)5   -6.9767     6.3798  -1.094   0.2781
factor(Month)6   10.2278     6.3715   1.605   0.1131
factor(Month)7    2.2894     6.3653   0.360   0.7202
factor(Month)8   -8.7918     6.3610  -1.382   0.1715
factor(Month)9   -2.8730     6.3587  -0.452   0.6529
factor(Month)10  11.4744     6.3584   1.805   0.0756 .
factor(Month)11   5.2503     6.3601   0.826   0.4120
factor(Month)12   8.3439     6.5827   1.268   0.2093
---
Signif. codes:  0 ‘***’ 0.001 ‘**’ 0.01 ‘*’ 0.05 ‘.’ 0.1 ‘ ’ 1

Residual standard error: 11.4 on 67 degrees of freedom
Multiple R-squared:  0.8446,     Adjusted R-squared:  0.8121
```

F-statistic: 26.01 on 14 and 67 DF, p-value: < 2.2e-16

```
> confint(mod1, level = 0.95)
                     2.5 %     97.5 %
(Intercept)     128.310091 151.581241
Time              1.771752   2.255267
Intervention    -52.305718 -31.476419
Post             -1.974390  -1.043039
factor(Month)2  -14.968587  10.645234
factor(Month)3   -4.307318  21.250129
factor(Month)4  -12.078512  13.430345
factor(Month)5  -19.710763   5.757332
factor(Month)6   -2.489804  22.945396
factor(Month)7  -10.415652  14.994550
factor(Month)8  -21.488316   3.904808
factor(Month)9  -15.564949   9.819033
factor(Month)10  -1.216982  24.165803
factor(Month)11  -7.444417  17.945117
factor(Month)12  -4.795117  21.482994
```

##

Supplementary Figure 15. Interrupted Time Series Analysis with Counterfactual Scenario for Psychostimulant Prescriptions in Young Adults Aged 18-22 Years.

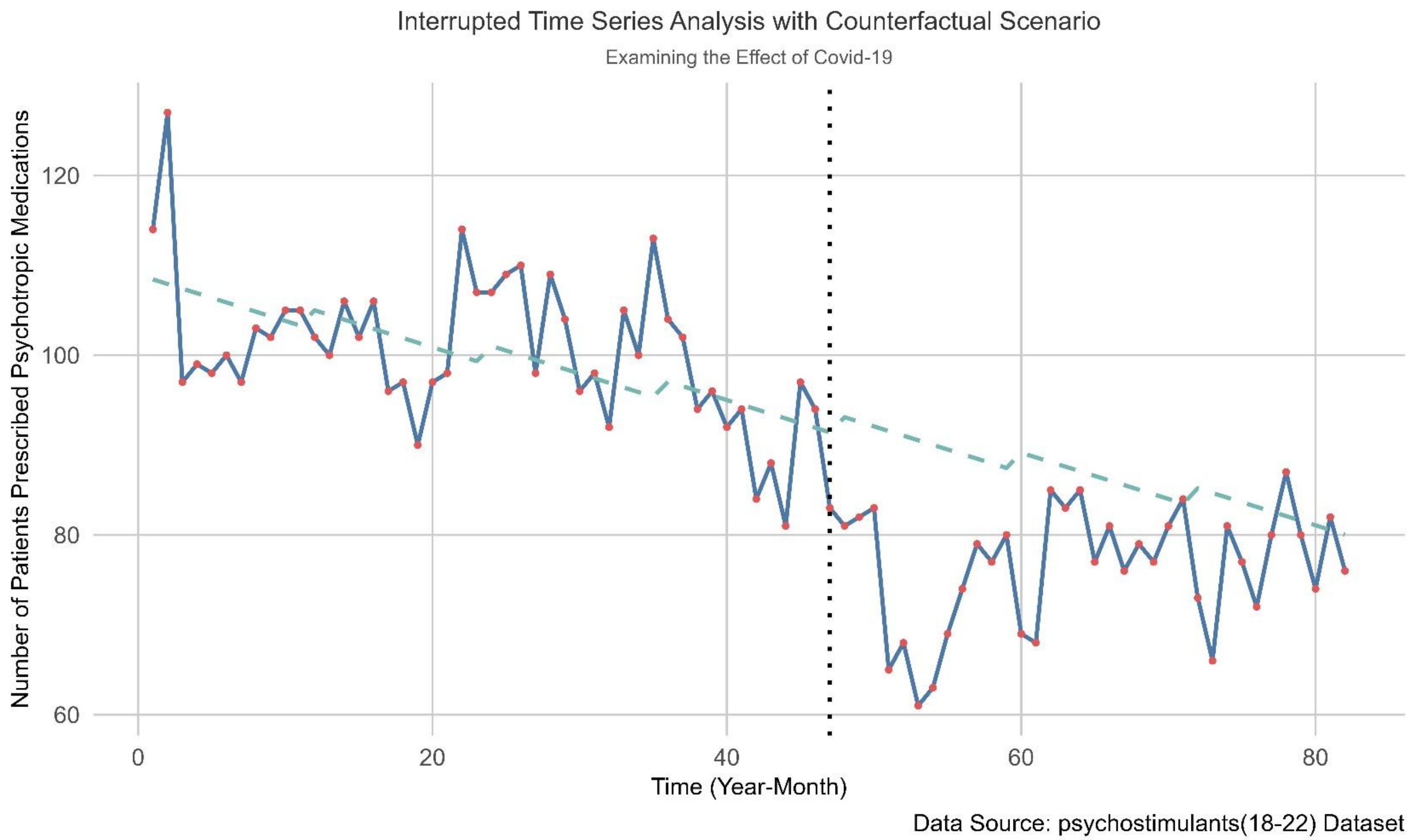

##

Estimate Std. Error t value Pr(>|t|)

(Intercept) 106.44000 3.42280 31.097 < 2e-16 ***

Time	-0.33099	0.07112	-4.654	1.59e-05	***
Intervention	-19.51801	3.06364	-6.371	1.99e-08	***
Post	0.58251	0.13699	4.252	6.71e-05	***
factor(Month)2	2.74713	3.76736	0.729	0.4684	
factor(Month)3	9.25705	3.75907	2.463	0.0164	*
factor(Month)4	-0.37590	3.75193	-0.100	0.9205	
factor(Month)5	1.56259	3.74593	0.417	0.6779	
factor(Month)6	-1.35607	3.74109	-0.362	0.7181	
factor(Month)7	-1.56044	3.73742	-0.418	0.6776	
factor(Month)8	-2.90766	3.73490	-0.779	0.4390	
factor(Month)9	-2.54061	3.73356	-0.680	0.4985	
factor(Month)10	3.25502	3.73338	0.872	0.3864	
factor(Month)11	4.33637	3.73438	1.161	0.2497	
factor(Month)12	5.86318	3.86507	1.517	0.1340	

Signif. codes: 0 ‘***’ 0.001 ‘**’ 0.01 ‘*’ 0.05 ‘.’ 0.1 ‘ ’ 1

Residual standard error: 6.694 on 67 degrees of freedom

Multiple R-squared: 0.8157, Adjusted R-squared: 0.7772

```
F-statistic: 21.18 on 14 and 67 DF,  p-value: < 2.2e-16


> confint(mod1, level = 0.95)
                      2.5 %      97.5 %
(Intercept)       99.6080693 113.2719304
Time              -0.4729389  -0.1890385
Intervention     -25.6330667 -13.4029599
Post               0.3090797   0.8559306
factor(Month)2    -4.7725585  10.2668224
factor(Month)3     1.7539069  16.7601871
factor(Month)4    -7.8647701   7.1129799
factor(Month)5    -5.9143169   9.0394997
factor(Month)6    -8.8233160   6.1111859
factor(Month)7    -9.0203477   5.8994763
factor(Month)8   -10.3625618   4.5472347
factor(Month)9    -9.9928200   4.9116087
factor(Month)10   -4.1968393  10.7068866
factor(Month)11   -3.1174770  11.7902116
factor(Month)12   -1.8515326  13.5778920
```

###

##

Results of subgroup analysis stratified by sex for antidepressant prescriptions.

##

Male:

```
Residuals:
    Min      1Q  Median      3Q     Max 
-12.2640  -3.1643   0.2055   3.7917  10.5170 


Coefficients:
               Estimate Std. Error t value Pr(>|t|)    
(Intercept)     20.7155     2.6423   7.840 4.68e-11 ***
Time             1.1946     0.0549  21.760  < 2e-16 ***
Intervention    -6.5566     2.3650  -2.772   0.0072 ** 
Post             2.2286     0.1057  21.075  < 2e-16 ***
factor(Month)2   -1.1398     2.9083  -0.392   0.6964    
factor(Month)3   -1.5753     2.9019  -0.543   0.5890    
factor(Month)4   -6.1536     2.8963  -2.125   0.0373 *  
factor(Month)5   -6.0176     2.8917  -2.081   0.0413 *  
```

factor(Month)6   -1.8817   2.8880 -0.652   0.5169

factor(Month)7   0.3971   2.8851   0.138   0.8909

factor(Month)8   -4.4669   2.8832 -1.549   0.1260

factor(Month)9   -3.4738   2.8822 -1.205   0.2323

factor(Month)10   0.8050   2.8820   0.279   0.7809

factor(Month)11 -0.4876   2.8828 -0.169   0.8662

factor(Month)12   2.9375   2.9837   0.985   0.3284

Signif. codes: 0 ‘***’ 0.001 ‘**’ 0.01 ‘*’ 0.05 ‘.’ 0.1 ‘ ’ 1

Residual standard error: 5.167 on 67 degrees of freedom

Multiple R-squared: 0.99,   Adjusted R-squared: 0.9879

F-statistic: 474.3 on 14 and 67 DF, p-value: < 2.2e-16

> confint(its_model_male, level = 0.95)

2.5 %   97.5 %

(Intercept)   15.441490 25.9894703

Time   1.085049 1.3042095

```
Intervention    -11.277193 -1.8360164
Post             2.017565  2.4397131
factor(Month)2   -6.944709  4.6651206
factor(Month)3   -7.367407  4.2168697
factor(Month)4  -11.934727 -0.3724743
factor(Month)5  -11.789535 -0.2457583
factor(Month)6   -7.646126  3.8827405
factor(Month)7   -5.361650  6.1558861
factor(Month)8  -10.221826  1.2879696
factor(Month)9   -9.226657  2.2789945
factor(Month)10  -4.947575  6.5575342
factor(Month)11  -6.241722  5.2664462
factor(Month)12  -3.017955  8.8929733
```

```
##################################################################
```

Female:

	Estimate	Std. Error	t value	Pr(>|t|)	
(Intercept)	33.14353	3.99311	8.300	6.92e-12	***
Time	1.05643	0.08297	12.733	< 2e-16	***
Intervention	8.40686	3.57411	2.352	0.0216	*
Post	3.56187	0.15981	22.288	< 2e-16	***
factor(Month)2	-6.72417	4.39509	-1.530	0.1307	
factor(Month)3	-1.16426	4.38542	-0.265	0.7915	
factor(Month)4	-7.89007	4.37708	-1.803	0.0760	.
factor(Month)5	-4.75873	4.37008	-1.089	0.2801	
factor(Month)6	-2.19883	4.36444	-0.504	0.6161	
factor(Month)7	0.64679	4.36015	0.148	0.8825	
factor(Month)8	-8.65044	4.35722	-1.985	0.0512	.
factor(Month)9	-5.51911	4.35565	-1.267	0.2095	
factor(Month)10	1.04080	4.35545	0.239	0.8119	
factor(Month)11	-2.25644	4.35660	-0.518	0.6062	
factor(Month)12	1.91039	4.50908	0.424	0.6732	

Signif. codes: 0 ‘***’ 0.001 ‘**’ 0.01 ‘*’ 0.05 ‘.’ 0.1 ‘ ’ 1

Residual standard error: 7.809 on 67 degrees of freedom

Multiple R-squared: 0.9876, Adjusted R-squared: 0.985

F-statistic: 380.1 on 14 and 67 DF, p-value: < 2.2e-16

	2.5 %	97.5 %
(Intercept)	25.1732469	41.11380739
Time	0.8908323	1.22203672
Intervention	1.2729085	15.54081970
Post	3.2428869	3.88085521
factor(Month)2	-15.4968039	2.04846821
factor(Month)3	-9.9175894	7.58906668
factor(Month)4	-16.6267553	0.84661691
factor(Month)5	-13.4814596	3.96399131
factor(Month)6	-10.9102866	6.51263132
factor(Month)7	-8.0561041	9.34969028
factor(Month)8	-17.3474913	0.04660474
factor(Month)9	-14.2130252	3.17480875
factor(Month)10	-7.6527088	9.73430525
factor(Month)11	-10.9522566	6.43938033

factor(Month)12 -7.0897613 10.91054437

###

Results of subgroup analysis stratified by sex for antipsychotic prescriptions.

###

Male:

	Estimate	Std. Error	t value	Pr(>|t|)	
(Intercept)	70.6710	4.5673	15.473	< 2e-16	***
Time	2.5191	0.0949	26.545	< 2e-16	***
Intervention	-5.1816	4.0880	-1.268	0.2094	
Post	1.4274	0.1828	7.809	5.32e-11	***
factor(Month)2	-5.8540	5.0271	-1.164	0.2484	
factor(Month)3	7.1581	5.0160	1.427	0.1582	
factor(Month)4	-0.1156	5.0065	-0.023	0.9816	
factor(Month)5	-6.2464	4.9985	-1.250	0.2158	
factor(Month)6	4.9085	4.9920	0.983	0.3290	
factor(Month)7	9.3491	4.9871	1.875	0.0652	.
factor(Month)8	3.2183	4.9838	0.646	0.5206	
factor(Month)9	1.9446	4.9820	0.390	0.6975	
factor(Month)10	6.2423	4.9817	1.253	0.2145	

```
factor(Month)11   0.8258     4.9831   0.166   0.8689

factor(Month)12   4.6615     5.1574   0.904   0.3693

---

Signif. codes:  0 ‘***’ 0.001 ‘**’ 0.01 ‘*’ 0.05 ‘.’ 0.1 ‘ ’ 1


Residual standard error: 8.932 on 67 degrees of freedom

Multiple R-squared:  0.9871,	Adjusted R-squared:  0.9844

F-statistic: 365.1 on 14 and 67 DF,  p-value: < 2.2e-16


>

> confint(its_model_male, level = 0.95)

                      2.5 %    97.5 %

(Intercept)      61.5546987 79.787411

Time              2.3296504  2.708480

Intervention    -13.3414115  2.978135

Post              1.0625753  1.792279

factor(Month)2  -15.8880467  4.180125

factor(Month)3   -2.8539249 17.170078
```

```
factor(Month)4  -10.1085668  9.877366
factor(Month)5  -16.2234184  3.730578
factor(Month)6   -5.0556373 14.872586
factor(Month)7   -0.6052355 19.303402
factor(Month)8   -6.7293650 13.165892
factor(Month)9   -7.9994606 11.888634
factor(Month)10  -3.7012399 16.185917
factor(Month)11  -9.1204178 10.772027
factor(Month)12  -5.6327770 14.955859
```

```
###############################################################
```

Female:

```
             Estimate Std. Error t value Pr(>|t|)
(Intercept)     87.0339     7.1709  12.137  < 2e-16 ***
Time            3.8074     0.1490  25.554  < 2e-16 ***
Intervention    -23.7528     6.4185  -3.701 0.000436 ***
Post            2.2600     0.2870   7.875 4.05e-11 ***
```

```
factor(Month)2   -8.2076     7.8928  -1.040 0.302135
factor(Month)3    6.3022     7.8754   0.800 0.426404
factor(Month)4   -9.9023     7.8605  -1.260 0.212125
factor(Month)5  -18.5355     7.8479  -2.362 0.021099 *
factor(Month)6   -4.3114     7.8378  -0.550 0.584092
factor(Month)7    2.9126     7.8301   0.372 0.711083
factor(Month)8  -14.0062     7.8248  -1.790 0.077976 .
factor(Month)9   -4.7822     7.8220  -0.611 0.543019
factor(Month)10   2.2990     7.8216   0.294 0.769721
factor(Month)11   0.8087     7.8237   0.103 0.917977
factor(Month)12  12.8941     8.0975   1.592 0.116014
---
Signif. codes:  0 '***' 0.001 '**' 0.01 '*' 0.05 '.' 0.1 ' ' 1

Residual standard error: 14.02 on 67 degrees of freedom
Multiple R-squared:  0.9845,     Adjusted R-squared:  0.9812
F-statistic: 303.4 on 14 and 67 DF,  p-value: < 2.2e-16
```

```
> summary(its_model_female)

Call:
lm(formula = Outcome ~ Time + Intervention + Post + factor(Month),
    data = data_female)

Residuals:
    Min      1Q  Median      3Q     Max
-39.876  -7.216   0.428   8.721  22.780

Coefficients:
               Estimate Std. Error t value Pr(>|t|)
(Intercept)     87.0339     7.1709  12.137  < 2e-16 ***
Time             3.8074     0.1490  25.554  < 2e-16 ***
Intervention   -23.7528     6.4185  -3.701 0.000436 ***
Post             2.2600     0.2870   7.875 4.05e-11 ***
factor(Month)2  -8.2076     7.8928  -1.040 0.302135
factor(Month)3   6.3022     7.8754   0.800 0.426404
```

```
factor(Month)4   -9.9023     7.8605  -1.260 0.212125
factor(Month)5  -18.5355     7.8479  -2.362 0.021099 *
factor(Month)6   -4.3114     7.8378  -0.550 0.584092
factor(Month)7    2.9126     7.8301   0.372 0.711083
factor(Month)8  -14.0062     7.8248  -1.790 0.077976 .
factor(Month)9   -4.7822     7.8220  -0.611 0.543019
factor(Month)10   2.2990     7.8216   0.294 0.769721
factor(Month)11   0.8087     7.8237   0.103 0.917977
factor(Month)12  12.8941     8.0975   1.592 0.116014
---
Signif. codes:  0 '***' 0.001 '**' 0.01 '*' 0.05 '.' 0.1 ' ' 1

Residual standard error: 14.02 on 67 degrees of freedom
Multiple R-squared:  0.9845,      Adjusted R-squared:  0.9812
F-statistic: 303.4 on 14 and 67 DF,  p-value: < 2.2e-16

> confint(its_model_female, level = 0.95)
                    2.5 %     97.5 %
```

(Intercept)	72.720684	101.347148
Time	3.509994	4.104779
Intervention	-36.564108	-10.941430
Post	1.687181	2.832860
factor(Month)2	-23.961679	7.546567
factor(Month)3	-9.417258	22.021641
factor(Month)4	-25.591909	5.787217
factor(Month)5	-34.199948	-2.870963
factor(Month)6	-19.955682	11.332838
factor(Month)7	-12.716273	18.541496
factor(Month)8	-29.624592	1.612168
factor(Month)9	-20.394936	10.830579
factor(Month)10	-13.313023	17.911019
factor(Month)11	-14.807427	16.424918
factor(Month)12	-3.268644	29.056763

##

Results of subgroup analysis stratified by sex for anxiolytic prescriptions.

##

Male:

```
    Min       1Q   Median       3Q      Max
-17.1337  -6.2565   0.4271   5.2259  18.0243


Coefficients:
              Estimate Std. Error t value Pr(>|t|)
(Intercept)     148.39204    4.71833  31.450  < 2e-16 ***
Time           -0.63878    0.09803  -6.516 1.10e-08 ***
Intervention    -20.72262    4.22323  -4.907 6.22e-06 ***
Post            2.64901    0.18884  14.028  < 2e-16 ***
factor(Month)2    7.64343    5.19332   1.472  0.14576
factor(Month)3   15.86121    5.18189   3.061  0.00317 **
factor(Month)4   -1.34958    5.17203  -0.261  0.79494
factor(Month)5   -2.70323    5.16377  -0.523  0.60235
factor(Month)6    2.37169    5.15710   0.460  0.64708
```

```
factor(Month)7    9.87519    5.15203   1.917  0.05954 .
factor(Month)8    2.80725    5.14857   0.545  0.58739
factor(Month)9    2.31075    5.14671   0.449  0.65490
factor(Month)10   5.38567    5.14647   1.046  0.29910
factor(Month)11   1.31773    5.14784   0.256  0.79875
factor(Month)12  11.57755    5.32800   2.173  0.03332 *
---
Signif. codes:  0 ‘***’ 0.001 ‘**’ 0.01 ‘*’ 0.05 ‘.’ 0.1 ‘ ’ 1

Residual standard error: 9.227 on 67 degrees of freedom
Multiple R-squared:  0.7706,     Adjusted R-squared:  0.7227
F-statistic: 16.08 on 14 and 67 DF,  p-value: 2.973e-16

> confint(its_model_male, level = 0.95)
                  2.5 %      97.5 %
(Intercept)    138.9742179 157.8098654
Time            -0.8344614  -0.4431044
Intervention   -29.1522267 -12.2930111
```

Post	2.2720911	3.0259257
factor(Month)2	-2.7224671	18.0093359
factor(Month)3	5.5181269	26.2043006
factor(Month)4	-11.6730008	8.9738440
factor(Month)5	-13.0101539	7.6036986
factor(Month)6	-7.9219190	12.6653082
factor(Month)7	-0.4083086	20.1586850
factor(Month)8	-7.4693321	13.0838386
factor(Month)9	-7.9621388	12.5836325
factor(Month)10	-4.8867322	15.6580703
factor(Month)11	-8.9573985	11.5928666
factor(Month)12	0.9428138	22.2122926

##

Female:

	Estimate	Std. Error	t value	Pr(>|t|)	
(Intercept)	197.8779	7.5377	26.252	< 2e-16	***
Time	-0.9685	0.1566	-6.184	4.24e-08	***

Intervention	-12.2518	6.7467	-1.816	0.0739	.
Post	4.1263	0.3017	13.678	< 2e-16	***
factor(Month)2	-1.7285	8.2965	-0.208	0.8356	
factor(Month)3	1.6145	8.2782	0.195	0.8460	
factor(Month)4	-2.3283	8.2625	-0.282	0.7790	
factor(Month)5	-0.5568	8.2493	-0.067	0.9464	
factor(Month)6	0.9290	8.2386	0.113	0.9106	
factor(Month)7	11.5577	8.2305	1.404	0.1649	
factor(Month)8	1.0435	8.2250	0.127	0.8994	
factor(Month)9	-6.3279	8.2220	-0.770	0.4442	
factor(Month)10	7.8722	8.2216	0.957	0.3418	
factor(Month)11	1.2152	8.2238	0.148	0.8830	
factor(Month)12	21.9069	8.5116	2.574	0.0123	*

Signif. codes: 0 ‘***’ 0.001 ‘**’ 0.01 ‘*’ 0.05 ‘.’ 0.1 ‘ ’ 1

Residual standard error: 14.74 on 67 degrees of freedom

Multiple R-squared: 0.7735, Adjusted R-squared: 0.7262

F-statistic: 16.35 on 14 and 67 DF, p-value: < 2.2e-16

```
> confint(its_model_female, level = 0.95)
                     2.5 %      97.5 %
(Intercept)     182.832688 212.9231491
Time             -1.281080  -0.6558762
Intervention    -25.718339   1.2147175
Post              3.524116   4.7283874
factor(Month)2  -18.288277  14.8313441
factor(Month)3  -14.908888  18.1378385
factor(Month)4  -18.820247  14.1636513
factor(Month)5  -17.022381  15.9088112
factor(Month)6  -15.515315  17.3733425
factor(Month)7   -4.870497  27.9858365
factor(Month)8  -15.373657  17.4605939
factor(Month)9  -22.739091  10.0833393
factor(Month)10  -8.538233  24.2826498
factor(Month)11 -15.199655  17.6299546
factor(Month)12   4.917653  38.8962257
>
```

##

Results of subgroup analysis stratified by sex for hypnotic and sedative prescriptions.

##

Male:

```
    Min       1Q   Median       3Q      Max
-24.4660  -6.3129  -0.0891   6.6051  26.7143


Coefficients:
               Estimate Std. Error t value Pr(>|t|)
(Intercept)    84.06180    5.57165  15.087   <2e-16 ***
Time            0.23383    0.11576   2.020   0.0474 *
Intervention    4.52679    4.98702   0.908   0.3673
Post            2.84264    0.22299  12.748   <2e-16 ***
factor(Month)2  -2.41581    6.13254  -0.394   0.6949
factor(Month)3   7.70351    6.11904   1.259   0.2124
factor(Month)4  -4.74860    6.10741  -0.778   0.4396
factor(Month)5  -7.62928    6.09765  -1.251   0.2152
factor(Month)6  -0.08139    6.08978  -0.013   0.9894
```

```
factor(Month)7   8.89507    6.08379   1.462   0.1484
factor(Month)8  13.30011    6.07970   2.188   0.0322 *
factor(Month)9   9.56228    6.07751   1.573   0.1203
factor(Month)10  9.25303    6.07723   1.523   0.1326
factor(Month)11 -0.34193    6.07884  -0.056   0.9553
factor(Month)12  3.01471    6.29159   0.479   0.6334
---
Signif. codes:  0 ‘***’ 0.001 ‘**’ 0.01 ‘*’ 0.05 ‘.’ 0.1 ‘ ’ 1

Residual standard error: 10.9 on 67 degrees of freedom
Multiple R-squared:  0.933,	Adjusted R-squared:  0.919
F-statistic: 66.68 on 14 and 67 DF,  p-value: < 2.2e-16

> confint(its_model_male, level = 0.95)
                   2.5 %     97.5 %
(Intercept)     72.940741570 95.1828677
Time             0.002764628  0.4648996
Intervention    -5.427339294 14.4809118
```

Post 2.397560331 3.2877280

factor(Month)2 -14.656418225 9.8247885

factor(Month)3 -4.510157078 19.9171681

factor(Month)4 -16.939044479 7.4418391

factor(Month)5 -19.800244576 4.5416799

factor(Month)6 -12.236632498 12.0738514

factor(Month)7 -3.248222846 21.0383682

factor(Month)8 1.164973235 25.4352415

factor(Month)9 -2.568480466 21.6930502

factor(Month)10 -2.877159475 21.3832271

factor(Month)11 -12.475350044 11.7914870

factor(Month)12 -9.543347765 15.5727748

##

Female:

Estimate Std. Error t value Pr(>|t|)

(Intercept) 84.3098 5.9670 14.129 < 2e-16 ***

Time 0.8034 0.1240 6.480 1.27e-08 ***

Intervention 0.5994 5.3409 0.112 0.91098

Post 3.2200 0.2388 13.484 < 2e-16 ***

factor(Month)2 -0.8533 6.5677 -0.130 0.89702

factor(Month)3 11.1061 6.5532 1.695 0.09477 .

factor(Month)4 4.4941 6.5408 0.687 0.49440

factor(Month)5 0.8821 6.5303 0.135 0.89295

factor(Month)6 8.2701 6.5219 1.268 0.20917

factor(Month)7 13.0867 6.5155 2.009 0.04862 *

factor(Month)8 18.7604 6.5111 2.881 0.00532 **

factor(Month)9 14.8627 6.5088 2.283 0.02558 *

factor(Month)10 13.9650 6.5085 2.146 0.03553 *

factor(Month)11 10.2101 6.5102 1.568 0.12152

factor(Month)12 14.5434 6.7380 2.158 0.03448 *

Signif. codes: 0 ‘***’ 0.001 ‘**’ 0.01 ‘*’ 0.05 ‘.’ 0.1 ‘ ’ 1

Residual standard error: 11.67 on 67 degrees of freedom

Multiple R-squared: 0.9593, Adjusted R-squared: 0.9508

F-statistic: 112.9 on 14 and 67 DF, p-value: < 2.2e-16

```
> confint(its_model_female, level = 0.95)
                      2.5 %    97.5 %
(Intercept)      72.39957527 96.219965
Time              0.55595587  1.050883
Intervention    -10.06108212 11.259825
Post              2.74336065  3.696693
factor(Month)2  -13.96248043 12.255871
factor(Month)3   -1.97420189 24.186445
factor(Month)4   -8.56133586 17.549574
factor(Month)5  -12.15247657 13.916710
factor(Month)6   -4.74764328 21.287871
factor(Month)7    0.08171979 26.091646
factor(Month)8    5.76417215 31.756618
factor(Month)9    1.87113417 27.854222
factor(Month)10   0.97403005 26.955893
factor(Month)11  -2.78428365 23.204487
```

factor(Month)12   1.09426841 27.992589

###

Results of subgroup analysis stratified by sex for psychostimulant prescriptions.

###

Male:

```
               Estimate Std. Error t value Pr(>|t|)
(Intercept)     99.61497    4.19379  23.753  < 2e-16 ***
Time             1.08522    0.08714  12.454  < 2e-16 ***
Intervention   -23.57689    3.75373  -6.281 2.86e-08 ***
Post             0.47022    0.16784   2.802  0.00664 **
factor(Month)2  -4.93930    4.61597  -1.070  0.28844
factor(Month)3   5.63110    4.60581   1.223  0.22576
factor(Month)4  -1.22708    4.59705  -0.267  0.79035
factor(Month)5  -7.22811    4.58971  -1.575  0.12000
factor(Month)6  -1.08629    4.58378  -0.237  0.81339
factor(Month)7  -2.51589    4.57927  -0.549  0.58455
factor(Month)8  -8.94549    4.57620  -1.955  0.05478 .
factor(Month)9  -7.37510    4.57455  -1.612  0.11162
factor(Month)10  2.76673    4.57433   0.605  0.54733
```

```
factor(Month)11   1.90855    4.57555   0.417  0.67792
factor(Month)12   4.57530    4.73568   0.966  0.33745
---
Signif. codes:  0 ‘***’ 0.001 ‘**’ 0.01 ‘*’ 0.05 ‘.’ 0.1 ‘ ’ 1

Residual standard error: 8.201 on 67 degrees of freedom
Multiple R-squared:  0.8915,	Adjusted R-squared:  0.8688
F-statistic: 39.32 on 14 and 67 DF,  p-value: < 2.2e-16

> confint(its_model_male, level = 0.95)
                    2.5 %      97.5 %
(Intercept)      91.2441386 107.9858088
Time              0.9112994   1.2591489
Intervention    -31.0693659 -16.0844058
Post              0.1352041   0.8052341
factor(Month)2  -14.1528134   4.2742148
factor(Month)3   -3.5621388  14.8243328
factor(Month)4  -10.4028356   7.9486793
```

factor(Month)5 -16.3892057   1.9329847

factor(Month)6 -10.2355482   8.0629769

factor(Month)7 -11.6561598   6.6243810

factor(Month)8 -18.0796205   0.1886342

factor(Month)9 -16.5059358   1.7557421

factor(Month)10 -6.3636804 11.8971363

factor(Month)11 -7.2242832 11.0413888

factor(Month)12 -4.8771679 14.0277622

##

Female:

	Estimate	Std. Error	t value	Pr(>|t|)	
(Intercept)	119.3417	5.4239	22.003	< 2e-16	***
Time	1.4979	0.1127	13.292	< 2e-16	***
Intervention	-34.9040	4.8548	-7.190	6.93e-10	***
Post	0.1552	0.2171	0.715	0.4771	
factor(Month)2	5.8238	5.9699	0.976	0.3328	
factor(Month)3	13.2593	5.9568	2.226	0.0294	*
factor(Month)4	1.8377	5.9455	0.309	0.7582	

```
factor(Month)5    0.4161    5.9359   0.070   0.9443
factor(Month)6    7.7088    5.9283   1.300   0.1979
factor(Month)7    4.2872    5.9225   0.724   0.4717
factor(Month)8   -4.7059    5.9185  -0.795   0.4294
factor(Month)9    2.5868    5.9163   0.437   0.6633
factor(Month)10  15.3081    5.9161   2.588   0.0118 *
factor(Month)11   8.6008    5.9176   1.453   0.1508
factor(Month)12  10.8830    6.1247   1.777   0.0801 .
---
Signif. codes:  0 '***' 0.001 '**' 0.01 '*' 0.05 '.' 0.1 ' ' 1

Residual standard error: 10.61 on 67 degrees of freedom
Multiple R-squared:  0.8672,	Adjusted R-squared:  0.8394
F-statistic: 31.25 on 14 and 67 DF,  p-value: < 2.2e-16

> confint(its_model_female, level = 0.95)
                    2.5 %      97.5 %
(Intercept)     108.5155503 130.1678472
```

Time	1.2729987	1.7228786
Intervention	-44.5941689	-25.2138561
Post	-0.2780663	0.5884954
factor(Month)2	-6.0922409	17.7397592
factor(Month)3	1.3695263	25.1490737
factor(Month)4	-10.0294707	13.7048667
factor(Month)5	-11.4321098	12.2643017
factor(Month)6	-4.1241227	19.5416820
factor(Month)7	-7.5340951	16.1084504
factor(Month)8	-16.5191807	7.1074748
factor(Month)9	-9.2222441	14.3959056
factor(Month)10	3.4995679	27.1166038
factor(Month)11	-3.2108881	20.4124272
factor(Month)12	-1.3420293	23.1080497

##

Results of subgroup analysis stratified by covid-19 time point (Dec 2019) for antidepressant prescriptions.

##

	Estimate	Std. Error	t value	Pr(>|t|)	
(Intercept)	61.1740	5.8703	10.421	1.18e-15	***
Time	2.4438	0.1317	18.562	< 2e-16	***
Intervention	-9.5529	5.3350	-1.791	0.0779	.
Post	6.4559	0.2306	27.993	< 2e-16	***
factor(Month)2	-9.3783	6.5300	-1.436	0.1556	
factor(Month)3	-4.0175	6.5285	-0.615	0.5404	
factor(Month)4	-15.6567	6.5289	-2.398	0.0193	*
factor(Month)5	-10.7244	6.5313	-1.642	0.1053	
factor(Month)6	-3.9350	6.5357	-0.602	0.5492	
factor(Month)7	1.9972	6.5420	0.305	0.7611	
factor(Month)8	-15.2134	6.5503	-2.323	0.0232	*
factor(Month)9	-10.1382	6.5605	-1.545	0.1270	
factor(Month)10	0.3654	6.5726	0.056	0.9558	
factor(Month)11	-3.9880	6.5867	-0.605	0.5469	

```
factor(Month)12   6.0051     6.7623   0.888   0.3777
---
Signif. codes:  0 '***' 0.001 '**' 0.01 '*' 0.05 '.' 0.1 ' ' 1

Residual standard error: 11.71 on 67 degrees of freedom
Multiple R-squared:  0.9924,     Adjusted R-squared:  0.9908
F-statistic: 623.9 on 14 and 67 DF,  p-value: < 2.2e-16

> confint(mod1, level = 0.95)
                    2.5 %    97.5 %
(Intercept)      49.456853 72.891228
Time              2.181008  2.706567
Intervention    -20.201520  1.095767
Post              5.995565  6.916229
factor(Month)2  -22.412280  3.655639
factor(Month)3  -17.048401  9.013417
factor(Month)4  -28.688441 -2.624887
factor(Month)5  -23.760970  2.312156
factor(Month)6  -16.980270  9.110255
```

factor(Month)7 -11.060618 15.055117

factor(Month)8 -28.287718 -2.138984

factor(Month)9 -23.232982 2.956509

factor(Month)10 -12.753537 13.484434

factor(Month)11 -17.135075 9.159057

factor(Month)12 -7.492612 19.502750

###

Results of subgroup analysis stratified by covid-19 time point (Jan 2020) for antidepressant prescriptions.

###

```
                Estimate Std. Error t value Pr(>|t|)
(Intercept)      61.26143    5.78408  10.591 5.94e-16 ***
Time              2.45690    0.12663  19.402  < 2e-16 ***
Intervention     -5.01394    5.29084  -0.948   0.3467
Post              6.50926    0.23246  28.001  < 2e-16 ***
factor(Month)2   -9.42679    6.46429  -1.458   0.1494
factor(Month)3   -4.10195    6.46322  -0.635   0.5278
factor(Month)4  -15.77711    6.46412  -2.441   0.0173 *
factor(Month)5  -10.88084    6.46700  -1.683   0.0971 .
factor(Month)6   -4.12743    6.47185  -0.638   0.5258
factor(Month)7    1.76884    6.47866   0.273   0.7857
factor(Month)8  -15.47775    6.48744  -2.386   0.0199 *
factor(Month)9  -10.43863    6.49817  -1.606   0.1129
factor(Month)10   0.02907    6.51084   0.004   0.9965
factor(Month)11  -4.36037    6.52545  -0.668   0.5063
```

```
factor(Month)12   4.12434    6.73987   0.612   0.5427
---
Signif. codes:  0 ‘***’ 0.001 ‘**’ 0.01 ‘*’ 0.05 ‘.’ 0.1 ‘ ’ 1

Residual standard error: 11.59 on 67 degrees of freedom
Multiple R-squared:  0.9925,     Adjusted R-squared:  0.991
F-statistic: 636.8 on 14 and 67 DF,  p-value: < 2.2e-16

> confint(mod1, level = 0.95)
                     2.5 %    97.5 %
(Intercept)      49.716363 72.806506
Time              2.204143  2.709664
Intervention    -15.574489  5.546613
Post              6.045268  6.973261
factor(Month)2  -22.329579  3.475992
factor(Month)3  -17.002597  8.798691
factor(Month)4  -28.679557 -2.874668
factor(Month)5  -23.789030  2.027342
factor(Month)6  -17.045296  8.790431
```

```
factor(Month)7  -11.162631 14.700305
factor(Month)8  -28.426739 -2.528765
factor(Month)9  -23.409030  2.531778
factor(Month)10 -12.966629 13.024772
factor(Month)11 -17.385228  8.664480
factor(Month)12  -9.328503 17.577173
```

##

Results of subgroup analysis stratified by covid-19 time point (Dec 2019) for antipsychotic prescriptions.

##

	Estimate	Std. Error	t value	Pr(>|t|)	
(Intercept)	170.4774	10.5547	16.152	< 2e-16	***
Time	6.8005	0.2193	31.010	< 2e-16	***
Intervention	-27.7353	9.4472	-2.936	0.00455	**
Post	4.4079	0.4224	10.435	1.11e-15	***
factor(Month)2	-13.7374	11.6172	-1.183	0.24119	
factor(Month)3	14.5730	11.5916	1.257	0.21305	
factor(Month)4	-11.9738	11.5696	-1.035	0.30442	
factor(Month)5	-24.8063	11.5511	-2.148	0.03537	*
factor(Month)6	0.3612	11.5362	0.031	0.97512	
factor(Month)7	12.3858	11.5249	1.075	0.28636	
factor(Month)8	-14.8752	11.5171	-1.292	0.20094	
factor(Month)9	-5.5649	11.5130	-0.483	0.63042	
factor(Month)10	8.3169	11.5124	0.722	0.47254	
factor(Month)11	-0.3727	11.5155	-0.032	0.97428	

```
factor(Month)12  23.1032    11.9185   1.938  0.05679 .
---
Signif. codes:  0 ‘***’ 0.001 ‘**’ 0.01 ‘*’ 0.05 ‘.’ 0.1 ‘ ’ 1

Residual standard error: 20.64 on 67 degrees of freedom
Multiple R-squared:  0.9904,     Adjusted R-squared:  0.9884
F-statistic: 493.8 on 14 and 67 DF,  p-value: < 2.2e-16

> confint(mod1, level = 0.95)
                      2.5 %     97.5 %
(Intercept)     149.4101248 191.544625
Time              6.3628257   7.238274
Intervention    -46.5919562  -8.878645
Post              3.5647293   5.251023
factor(Month)2  -36.9254401   9.450676
factor(Month)3   -8.5640444  37.710000
factor(Month)4  -35.0668384  11.119229
factor(Month)5  -47.8624341  -1.750169
factor(Month)6  -22.6651512  23.387555
```

```
factor(Month)7  -10.6178744  35.389570
factor(Month)8  -37.8634820   8.113041
factor(Month)9  -28.5448456  17.415125
factor(Month)10 -14.6619731  31.295831
factor(Month)11 -23.3577225  22.612301
factor(Month)12  -0.6862608  46.892611
```

##

Results of subgroup analysis stratified by covid-19 time point (Jan 2020) for antipsychotic prescriptions.

##

	Estimate	Std. Error	t value	Pr(>|t|)	
(Intercept)	175.0826	10.4188	16.804	< 2e-16	***
Time	6.8038	0.2281	29.828	< 2e-16	***
Intervention	-29.3491	9.5303	-3.080	0.0030	**
Post	4.2779	0.4187	10.216	2.68e-15	***
factor(Month)2	-18.9347	11.6441	-1.626	0.1086	
factor(Month)3	9.4282	11.6421	0.810	0.4209	
factor(Month)4	-17.0661	11.6438	-1.466	0.1474	
factor(Month)5	-29.8461	11.6489	-2.562	0.0127	*
factor(Month)6	-4.6261	11.6577	-0.397	0.6928	
factor(Month)7	7.4511	11.6700	0.638	0.5253	
factor(Month)8	-19.7575	11.6858	-1.691	0.0955	.
factor(Month)9	-10.3946	11.7051	-0.888	0.3777	
factor(Month)10	3.5397	11.7279	0.302	0.7637	
factor(Month)11	-5.0974	11.7542	-0.434	0.6659	

```
factor(Month)12  18.1715    12.1405   1.497   0.1391
---
Signif. codes:  0 '***' 0.001 '**' 0.01 '*' 0.05 '.' 0.1 ' ' 1

Residual standard error: 20.88 on 67 degrees of freedom
Multiple R-squared:  0.9902,     Adjusted R-squared:  0.9881
F-statistic: 482.3 on 14 and 67 DF,  p-value: < 2.2e-16

> confint(mod1, level = 0.95)
                    2.5 %     97.5 %
(Intercept)     154.286612 195.878666
Time              6.348467   7.259057
Intervention    -48.371678 -10.326438
Post              3.442079   5.113664
factor(Month)2  -42.176320   4.307010
factor(Month)3  -13.809598  32.666016
factor(Month)4  -40.307120   6.174982
factor(Month)5  -53.097455  -6.594668
factor(Month)6  -27.894879  18.642771
```

```
factor(Month)7  -15.842234  30.744427
factor(Month)8  -43.082354   3.567419
factor(Month)9  -33.758068  12.968863
factor(Month)10 -19.869342  26.948723
factor(Month)11 -28.558991  18.364101
factor(Month)12  -6.060926  42.404012
```

##

Results of subgroup analysis stratified by covid-19 time point (Jan 2020) for anxiolytic prescriptions.

##

	Estimate	Std. Error	t value	Pr(>|t|)	
(Intercept)	389.9296	11.5029	33.899	< 2e-16	***
Time	-1.9755	0.2518	-7.844	4.59e-11	***
Intervention	-35.1641	10.5219	-3.342	0.00136	**
Post	7.5483	0.4623	16.328	< 2e-16	***
factor(Month)2	-0.3923	12.8556	-0.031	0.97575	
factor(Month)3	11.7768	12.8535	0.916	0.36283	
factor(Month)4	-11.3398	12.8553	-0.882	0.38087	
factor(Month)5	-10.8850	12.8610	-0.846	0.40037	
factor(Month)6	-1.0016	12.8706	-0.078	0.93820	
factor(Month)7	17.8818	12.8842	1.388	0.16977	
factor(Month)8	-1.3777	12.9016	-0.107	0.91528	
factor(Month)9	-11.2086	12.9230	-0.867	0.38885	
factor(Month)10	10.9605	12.9482	0.846	0.40029	
factor(Month)11	-0.0133	12.9772	-0.001	0.99919	

```
factor(Month)12  34.5132    13.4037   2.575  0.01224 *
---
Signif. codes:  0 ‘***’ 0.001 ‘**’ 0.01 ‘*’ 0.05 ‘.’ 0.1 ‘ ’ 1

Residual standard error: 23.06 on 67 degrees of freedom
Multiple R-squared:  0.8161,     Adjusted R-squared:  0.7777
F-statistic: 21.24 on 14 and 67 DF,  p-value: < 2.2e-16

> confint(mod1, level = 0.95)
                    2.5 %      97.5 %
(Intercept)     366.969762 412.889354
Time             -2.478151  -1.472816
Intervention    -56.166022 -14.162280
Post              6.625491   8.471000
factor(Month)2  -26.052169  25.267623
factor(Month)3  -13.878818  37.432455
factor(Month)4  -36.999021  14.319414
factor(Month)5  -36.555634  14.785639
factor(Month)6  -26.691502  24.688262
```

```
factor(Month)7   -7.835179  43.598695
factor(Month)8  -27.129498  24.374056
factor(Month)9  -37.002999  14.585740
factor(Month)10 -14.884214  36.805140
factor(Month)11 -25.915957  25.889353
factor(Month)12   7.759450  61.267031
```

##

Results of subgroup analysis stratified by covid-19 time point (Dec 2019) for anxiolytic prescriptions.

##

	Estimate	Std. Error	t value	Pr(>|t|)	
(Intercept)	389.6808	11.9824	32.521	< 2e-16	***
Time	-2.0304	0.2687	-7.556	1.52e-10	***
Intervention	-34.9815	10.8896	-3.212	0.00202	**
Post	7.3467	0.4707	15.606	< 2e-16	***
factor(Month)2	-0.2154	13.3289	-0.016	0.98715	
factor(Month)3	12.0950	13.3258	0.908	0.36732	
factor(Month)4	-10.8803	13.3267	-0.816	0.41715	
factor(Month)5	-10.2842	13.3316	-0.771	0.44317	
factor(Month)6	-0.2595	13.3405	-0.019	0.98454	
factor(Month)7	18.7651	13.3534	1.405	0.16456	
factor(Month)8	-0.3530	13.3703	-0.026	0.97901	
factor(Month)9	-10.0426	13.3911	-0.750	0.45591	
factor(Month)10	12.2678	13.4159	0.914	0.36377	
factor(Month)11	1.4353	13.4446	0.107	0.91530	

```
factor(Month)12  41.4763    13.8032   3.005  0.00374 **
---
Signif. codes:  0 ‘***’ 0.001 ‘**’ 0.01 ‘*’ 0.05 ‘.’ 0.1 ‘ ’ 1

Residual standard error: 23.9 on 67 degrees of freedom
Multiple R-squared:  0.8023,     Adjusted R-squared:  0.761
F-statistic: 19.42 on 14 and 67 DF,  p-value: < 2.2e-16

> confint(mod1, level = 0.95)
                     2.5 %     97.5 %
(Intercept)     365.763875 413.597716
Time             -2.566776  -1.494015
Intervention    -56.717358 -13.245704
Post              6.407033   8.286275
factor(Month)2  -26.820119  26.389265
factor(Month)3  -14.503492  38.693440
factor(Month)4  -37.480578  15.719899
factor(Month)5  -36.894231  16.325783
factor(Month)6  -26.887301  26.368227
```

```
factor(Month)7   -7.888344  45.418642
factor(Month)8  -27.040192  26.334150
factor(Month)9  -36.771388  16.686147
factor(Month)10 -14.510465  39.046027
factor(Month)11 -25.400239  28.270888
factor(Month)12  13.925033  69.027497
```

##

Results of subgroup analysis stratified by covid-19 time point (Jan 2020) for hypnotic and sedative prescriptions.

##

	Estimate	Std. Error	t value	Pr(>|t|)	
(Intercept)	186.5580	9.6320	19.369	< 2e-16	***
Time	0.9267	0.2109	4.395	4.05e-05	***
Intervention	8.2696	8.8106	0.939	0.35131	
Post	6.8973	0.3871	17.817	< 2e-16	***
factor(Month)2	-6.9625	10.7647	-0.647	0.51998	
factor(Month)3	19.0119	10.7629	1.766	0.08188	.
factor(Month)4	-1.7279	10.7644	-0.161	0.87295	
factor(Month)5	-9.8964	10.7692	-0.919	0.36142	
factor(Month)6	6.2209	10.7773	0.577	0.56573	
factor(Month)7	23.4810	10.7887	2.176	0.03305	*
factor(Month)8	30.3125	10.8033	2.806	0.00656	**
factor(Month)9	22.8584	10.8211	2.112	0.03838	*
factor(Month)10	23.4042	10.8422	2.159	0.03447	*
factor(Month)11	11.3786	10.8666	1.047	0.29881	

```
factor(Month)12  19.7708    11.2236   1.762  0.08271 .
---
Signif. codes:  0 ‘***’ 0.001 ‘**’ 0.01 ‘*’ 0.05 ‘.’ 0.1 ‘ ’ 1

Residual standard error: 19.31 on 67 degrees of freedom
Multiple R-squared:  0.9693,     Adjusted R-squared:  0.9629
F-statistic: 151.1 on 14 and 67 DF,  p-value: < 2.2e-16

> confint(mod1, level = 0.95)
                      2.5 %     97.5 %
(Intercept)     167.3324949 205.783568
Time              0.5058317   1.347655
Intervention     -9.3164930  25.855617
Post              6.1246515   7.670001
factor(Month)2  -28.4489422  14.524024
factor(Month)3   -2.4709732  40.494859
factor(Month)4  -23.2138553  19.757975
factor(Month)5  -31.3918715  11.599082
factor(Month)6  -15.2907274  27.732456
```

factor(Month)7	1.9467346	45.015228
factor(Month)8	8.7491066	51.875946
factor(Month)9	1.2592720	44.457443
factor(Month)10	1.7629774	45.045399
factor(Month)11	-10.3111683	33.068350
factor(Month)12	-2.6316833	42.173242

##

Results of subgroup analysis stratified by covid-19 time point (Dec 2019) for hypnotic and sedative prescriptions.

##

	Estimate	Std. Error	t value	Pr(>|t|)	
(Intercept)	187.8677	9.6109	19.547	< 2e-16	***
Time	0.8195	0.2155	3.802	0.000313	***
Intervention	8.5272	8.7345	0.976	0.332444	
Post	6.8384	0.3776	18.111	< 2e-16	***
factor(Month)2	-7.0171	10.6910	-0.656	0.513846	
factor(Month)3	19.0898	10.6885	1.786	0.078622	.
factor(Month)4	-1.5176	10.6892	-0.142	0.887530	
factor(Month)5	-9.5535	10.6931	-0.893	0.374828	
factor(Month)6	6.6962	10.7003	0.626	0.533572	
factor(Month)7	24.0889	10.7106	2.249	0.027800	*
factor(Month)8	31.0529	10.7242	2.896	0.005105	**
factor(Month)9	23.7312	10.7409	2.209	0.030567	*
factor(Month)10	24.4095	10.7608	2.268	0.026533	*
factor(Month)11	12.5164	10.7838	1.161	0.249894	

```
factor(Month)12  19.4054    11.0714   1.753 0.084219 .
---
Signif. codes:  0 ‘***’ 0.001 ‘**’ 0.01 ‘*’ 0.05 ‘.’ 0.1 ‘ ’ 1

Residual standard error: 19.17 on 67 degrees of freedom
Multiple R-squared:  0.9697,     Adjusted R-squared:  0.9634
F-statistic: 153.3 on 14 and 67 DF,  p-value: < 2.2e-16

> confint(mod1, level = 0.95)
                  2.5 %     97.5 %
(Intercept)     168.6841818 207.051203
Time              0.3892775   1.249728
Intervention     -8.9068975  25.961259
Post              6.0847410   7.592061
factor(Month)2  -28.3564031  14.322285
factor(Month)3   -2.2445122  40.424188
factor(Month)4  -22.8533224  19.818221
factor(Month)5  -30.8971181  11.790096
factor(Month)6  -14.6616066  28.054093
```

factor(Month)7	2.7103678	45.467341
factor(Month)8	9.6473950	52.458393
factor(Month)9	2.2923562	45.170083
factor(Month)10	2.9309954	45.888095
factor(Month)11	-9.0080811	34.040966
factor(Month)12	-2.6931839	41.503923

##

Results of subgroup analysis stratified by time point (Jan 2020) for psychostimulant prescriptions.

##

```
                Estimate Std. Error t value Pr(>|t|)
(Intercept)     247.4104     7.2346  34.198  < 2e-16 ***
Time              2.8073     0.1584  17.724  < 2e-16 ***
Intervention    -65.2730     6.6176  -9.864 1.12e-14 ***
Post              0.6368     0.2908   2.190  0.03201 *
factor(Month)2   -9.5674     8.0854  -1.183  0.24087
factor(Month)3    8.6382     8.0840   1.069  0.28911
factor(Month)4  -10.1563     8.0851  -1.256  0.21342
factor(Month)5  -17.9507     8.0887  -2.219  0.02986 *
factor(Month)6   -1.8881     8.0948  -0.233  0.81628
factor(Month)7   -8.3968     8.1033  -1.036  0.30383
factor(Month)8  -26.0484     8.1143  -3.210  0.00204 **
factor(Month)9  -16.7000     8.1277  -2.055  0.04381 *
factor(Month)10   8.3627     8.1436   1.027  0.30816
factor(Month)11   0.4254     8.1618   0.052  0.95859
```

```
factor(Month)12   4.3074     8.4300   0.511  0.61106
---
Signif. codes:  0 ‘***’ 0.001 ‘**’ 0.01 ‘*’ 0.05 ‘.’ 0.1 ‘ ’ 1

Residual standard error: 14.5 on 67 degrees of freedom
Multiple R-squared:  0.9323,     Adjusted R-squared:  0.9182
F-statistic: 65.92 on 14 and 67 DF,  p-value: < 2.2e-16

> confint(mod1, level = 0.95)
                      2.5 %     97.5 %
(Intercept)     232.97016873 261.850666
Time              2.49112966   3.123421
Intervention    -78.48188385 -52.064207
Post              0.05640246   1.217110
factor(Month)2  -25.70581278   6.571066
factor(Month)3   -7.49759052  24.773930
factor(Month)4  -26.29429955   5.981726
factor(Month)5  -34.09593781  -1.805549
factor(Month)6  -18.04535586  14.269241
```

factor(Month)7	-24.57111409	7.777515
factor(Month)8	-42.24462558	-9.852173
factor(Month)9	-32.92301335	-0.476984
factor(Month)10	-7.89196758	24.617343
factor(Month)11	-15.86574571	16.716493
factor(Month)12	-12.51907588	21.133782

##

Results of subgroup analysis stratified by time point (Dec 2019) for psychostimulant prescriptions.

##

	Estimate	Std. Error	t value	Pr(>|t|)	
(Intercept)	245.0373	7.2311	33.887	< 2e-16	***
Time	2.8891	0.1622	17.815	< 2e-16	***
Intervention	-65.2943	6.5717	-9.936	8.32e-15	***
Post	0.4256	0.2841	1.498	0.13877	
factor(Month)2	-9.2197	8.0437	-1.146	0.25579	
factor(Month)3	8.9945	8.0418	1.118	0.26737	
factor(Month)4	-9.7914	8.0424	-1.217	0.22769	
factor(Month)5	-17.5772	8.0453	-2.185	0.03241	*
factor(Month)6	-1.5059	8.0507	-0.187	0.85219	
factor(Month)7	-8.0060	8.0585	-0.993	0.32405	
factor(Month)8	-25.6489	8.0687	-3.179	0.00224	**
factor(Month)9	-16.2919	8.0812	-2.016	0.04781	*
factor(Month)10	8.7794	8.0962	1.084	0.28208	
factor(Month)11	0.8507	8.1135	0.105	0.91681	

```
factor(Month)12  15.2686     8.3299   1.833  0.07125 .
---
Signif. codes:  0 ‘***’ 0.001 ‘**’ 0.01 ‘*’ 0.05 ‘.’ 0.1 ‘ ’ 1

Residual standard error: 14.43 on 67 degrees of freedom
Multiple R-squared:  0.933,       Adjusted R-squared:  0.919
F-statistic: 66.65 on 14 and 67 DF,  p-value: < 2.2e-16

> confint(mod1, level = 0.95)
                     2.5 %      97.5 %
(Intercept)     230.6039802 259.4706073
Time              2.5654317   3.2128184
Intervention    -78.4113293 -52.1771825
Post             -0.1414117   0.9926674
factor(Month)2  -25.2750439   6.8356002
factor(Month)3   -7.0571092  25.0460201
factor(Month)4  -25.8440014   6.2612668
factor(Month)5  -33.6357195  -1.5186606
factor(Month)6  -17.5751152  14.5633755
```

```
factor(Month)7  -24.0907505   8.0787938
factor(Month)8  -41.7540399  -9.5438480
factor(Month)9  -32.4221081  -0.1617109
factor(Month)10  -7.3806473  24.9394685
factor(Month)11 -15.3439167  17.0453782
factor(Month)12  -1.3579314  31.8951428
```

Supplementary Figure 16. Residual versus Fitted Plot (Antidepressant Prescriptions).

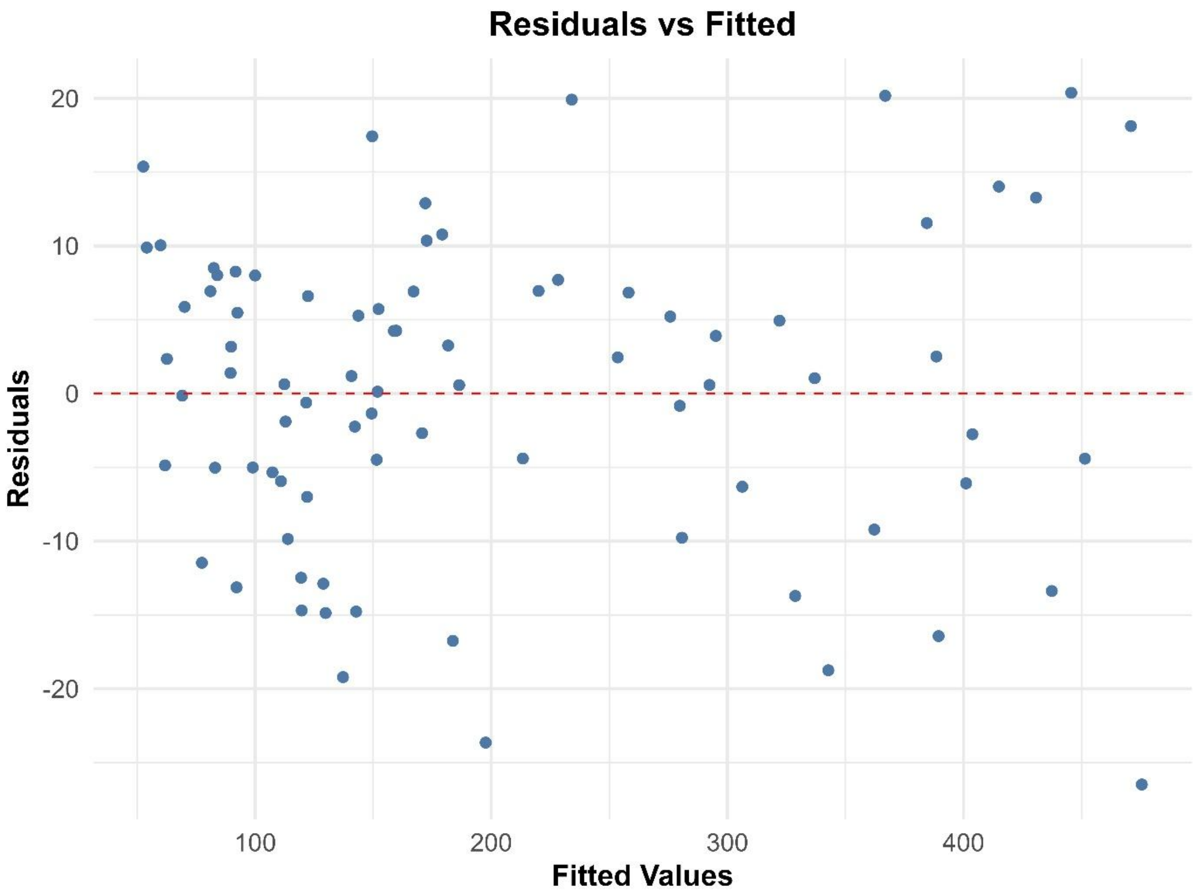

Supplementary Figure 17. Residual versus Fitted Plot (Antipsychotic Prescriptions).

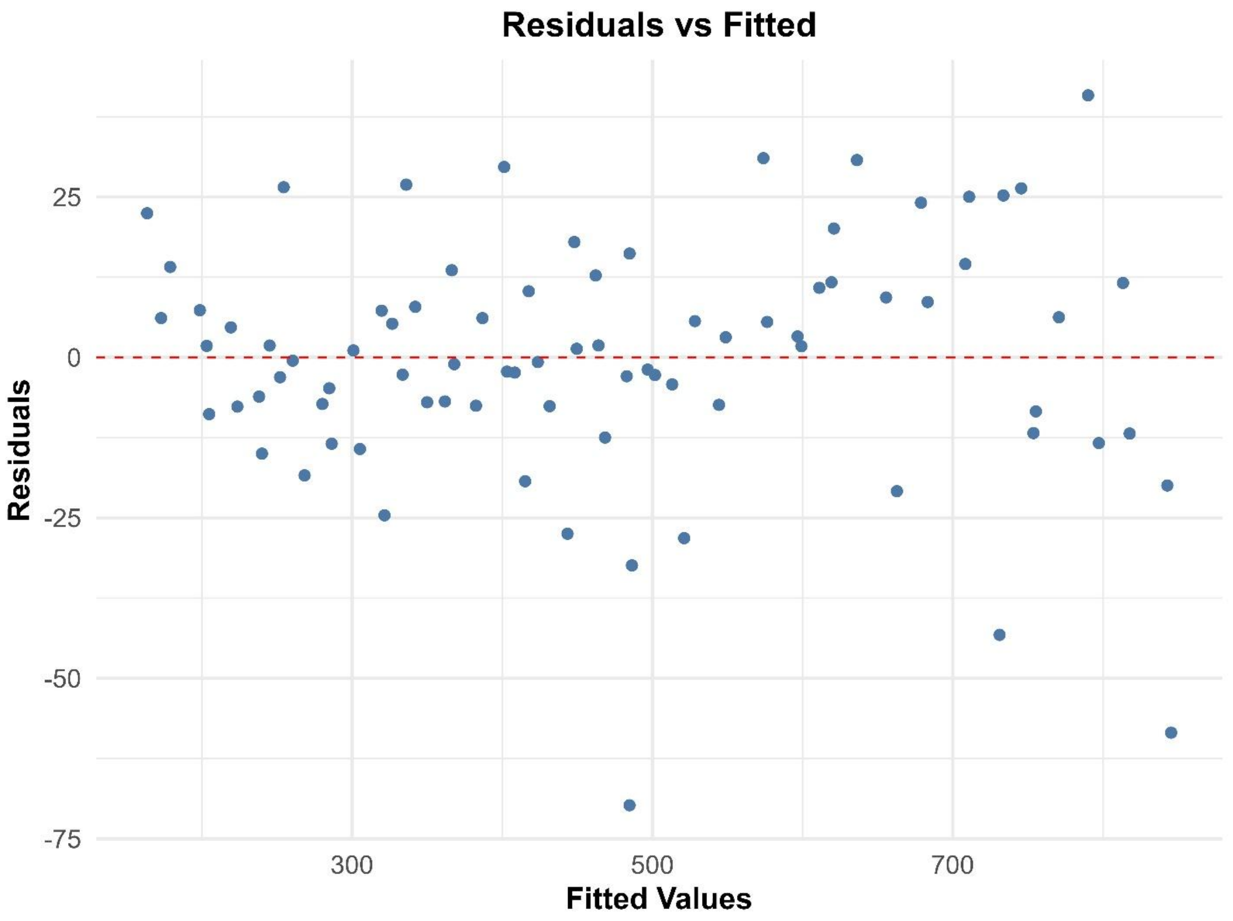

Supplementary Figure 18. Residual versus Fitted Plot (Anxiolytic Prescriptions).

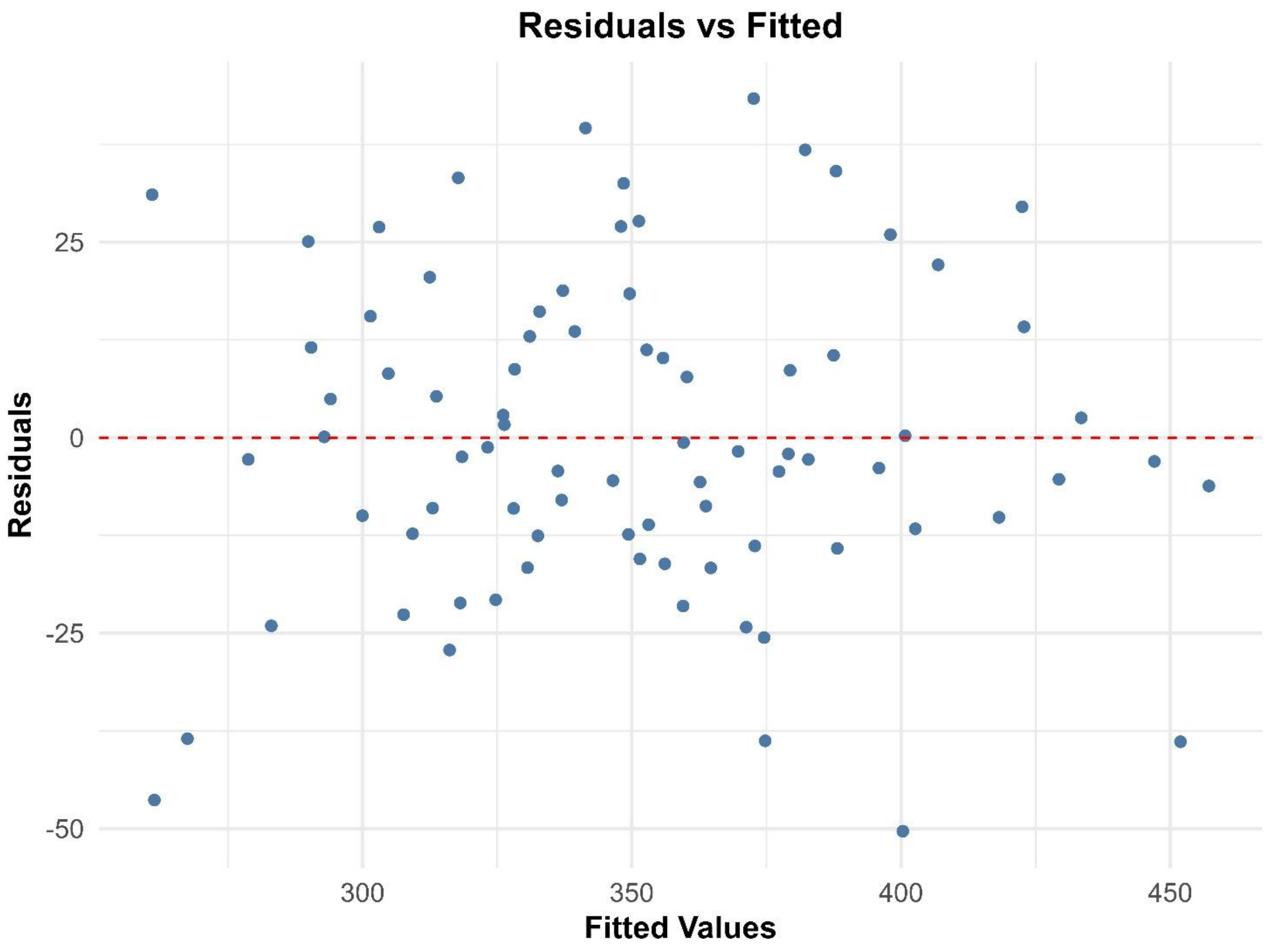

Supplementary Figure 19. Residual versus Fitted Plot (Hypnotic and Sedative Prescriptions).

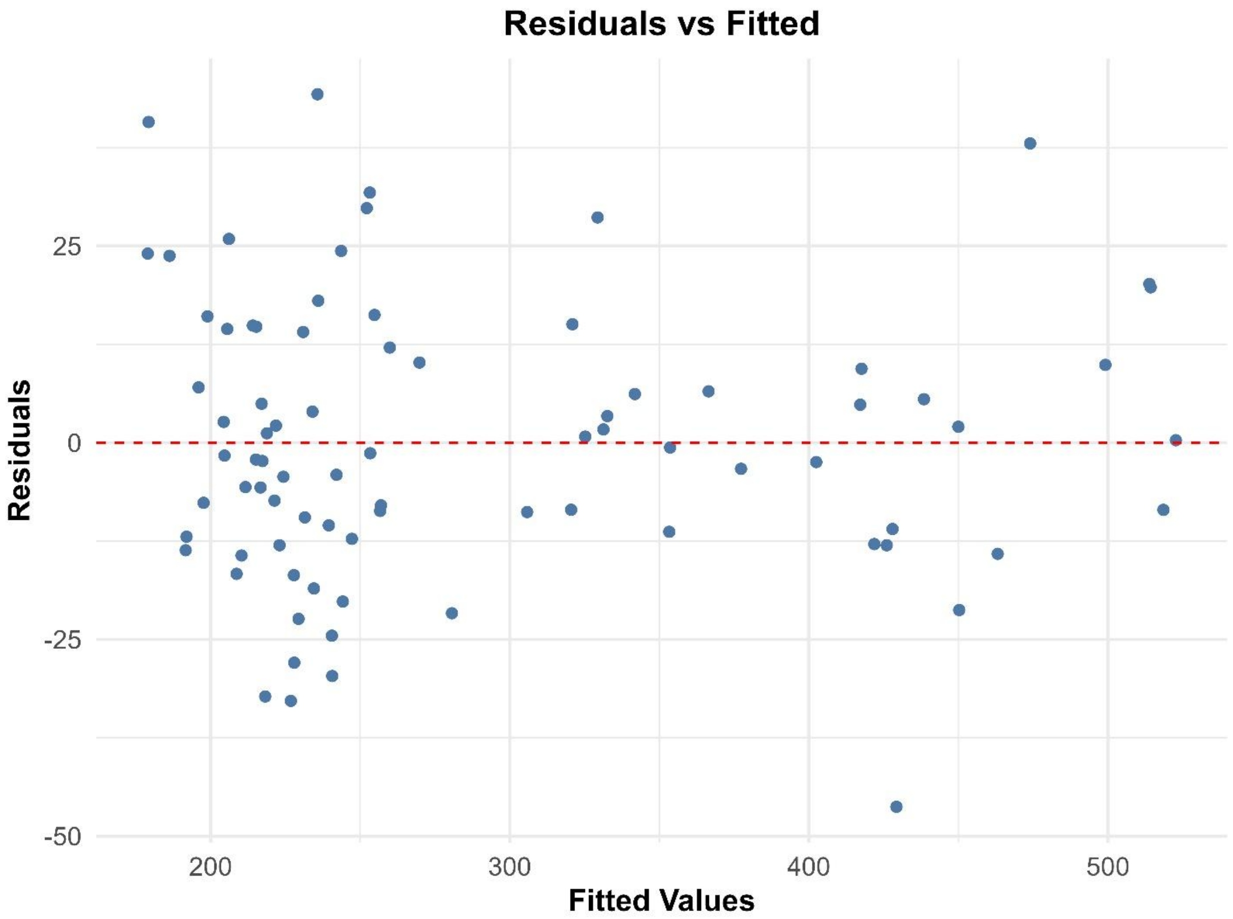

Supplementary Figure 20. Residual versus Fitted Plot (Psychostimulant Prescriptions).

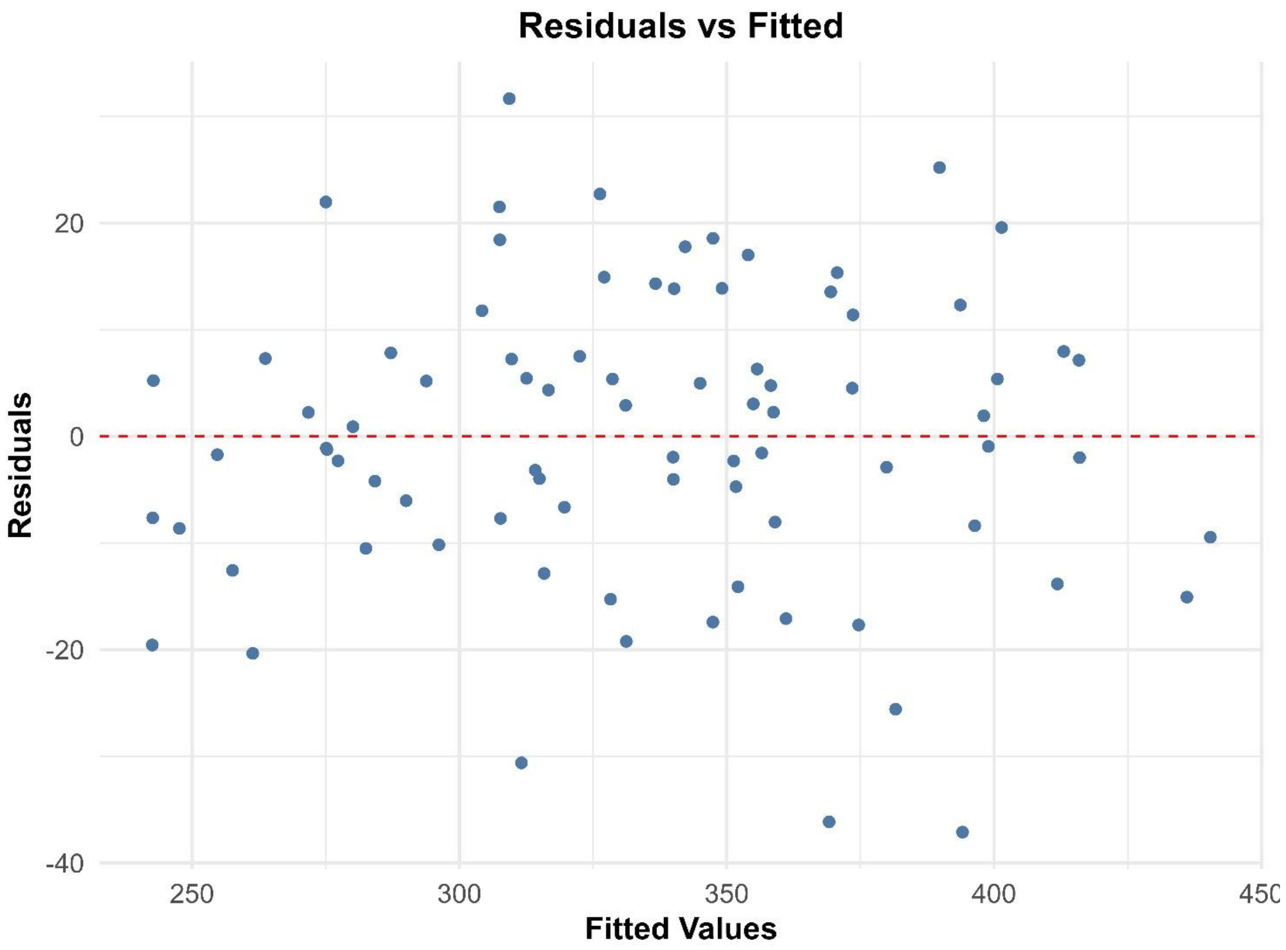

Supplementary Figure 21. Autocorrelation Function Plot (Antidepressant Prescriptions).

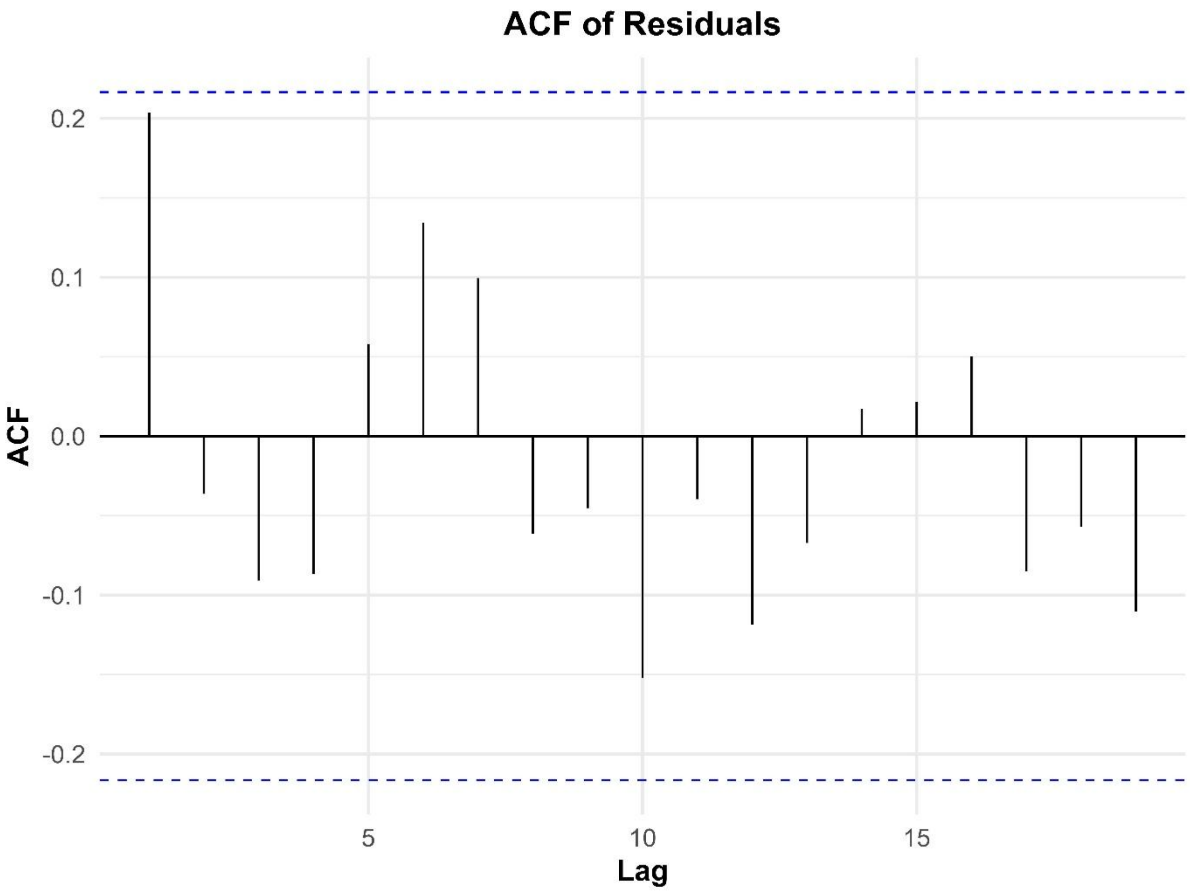

Supplementary Figure 22. Autocorrelation Function Plot (Antipsychotic Prescriptions).

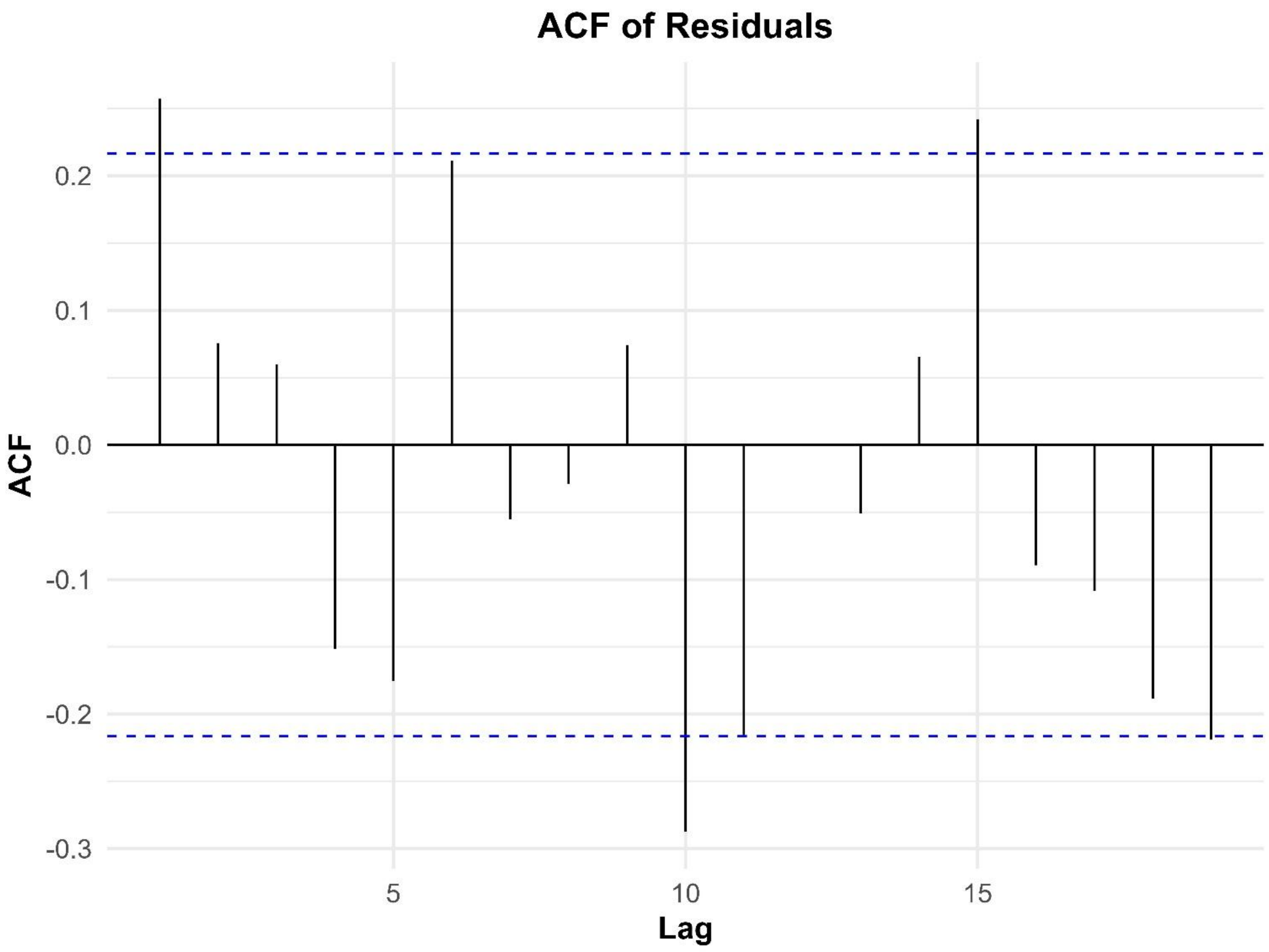

Supplementary Figure 23. Autocorrelation Function Plot (Anxiolytic Prescriptions).

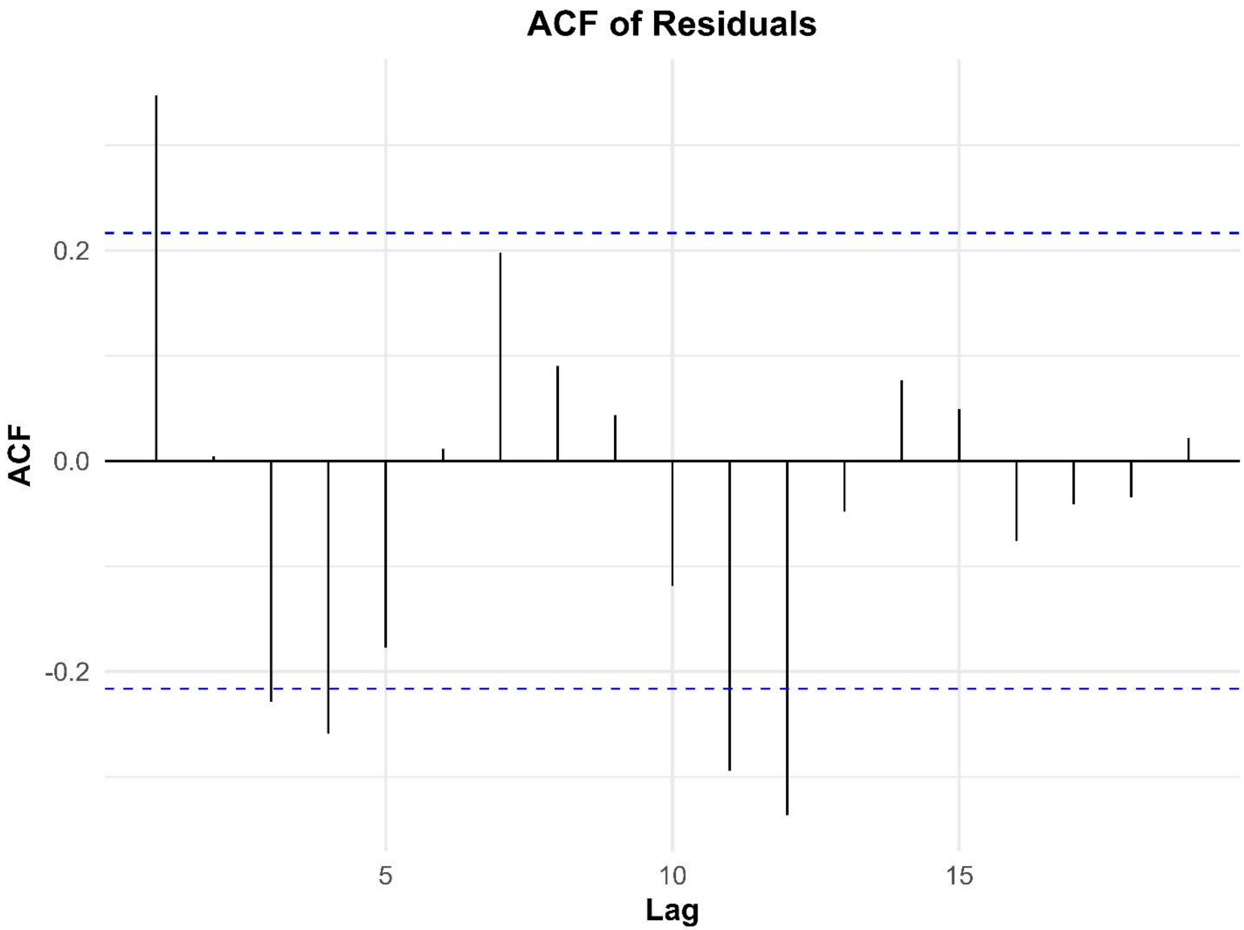

Supplementary Figure 24. Autocorrelation Function Plot (Hypnotic and Sedative Prescriptions).

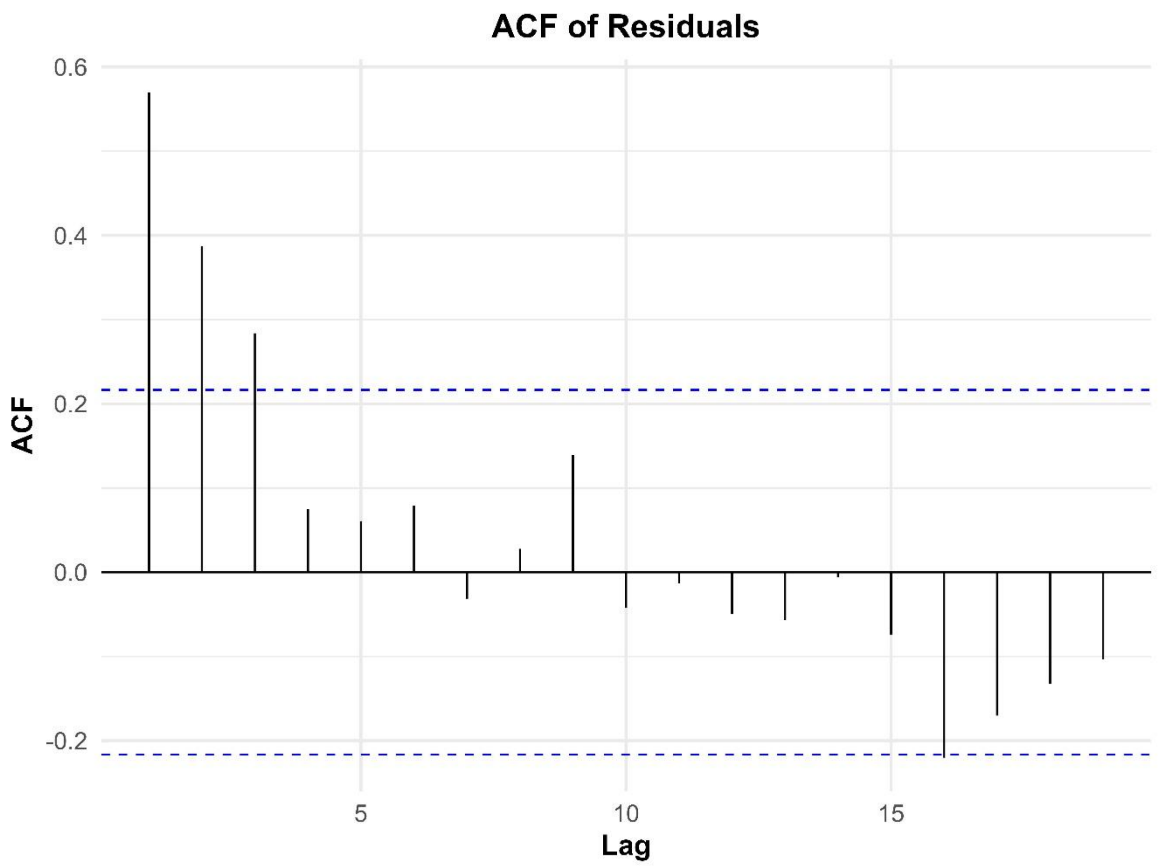

Supplementary Figure 25. Autocorrelation Function Plot (Psychostimulant Prescriptions).

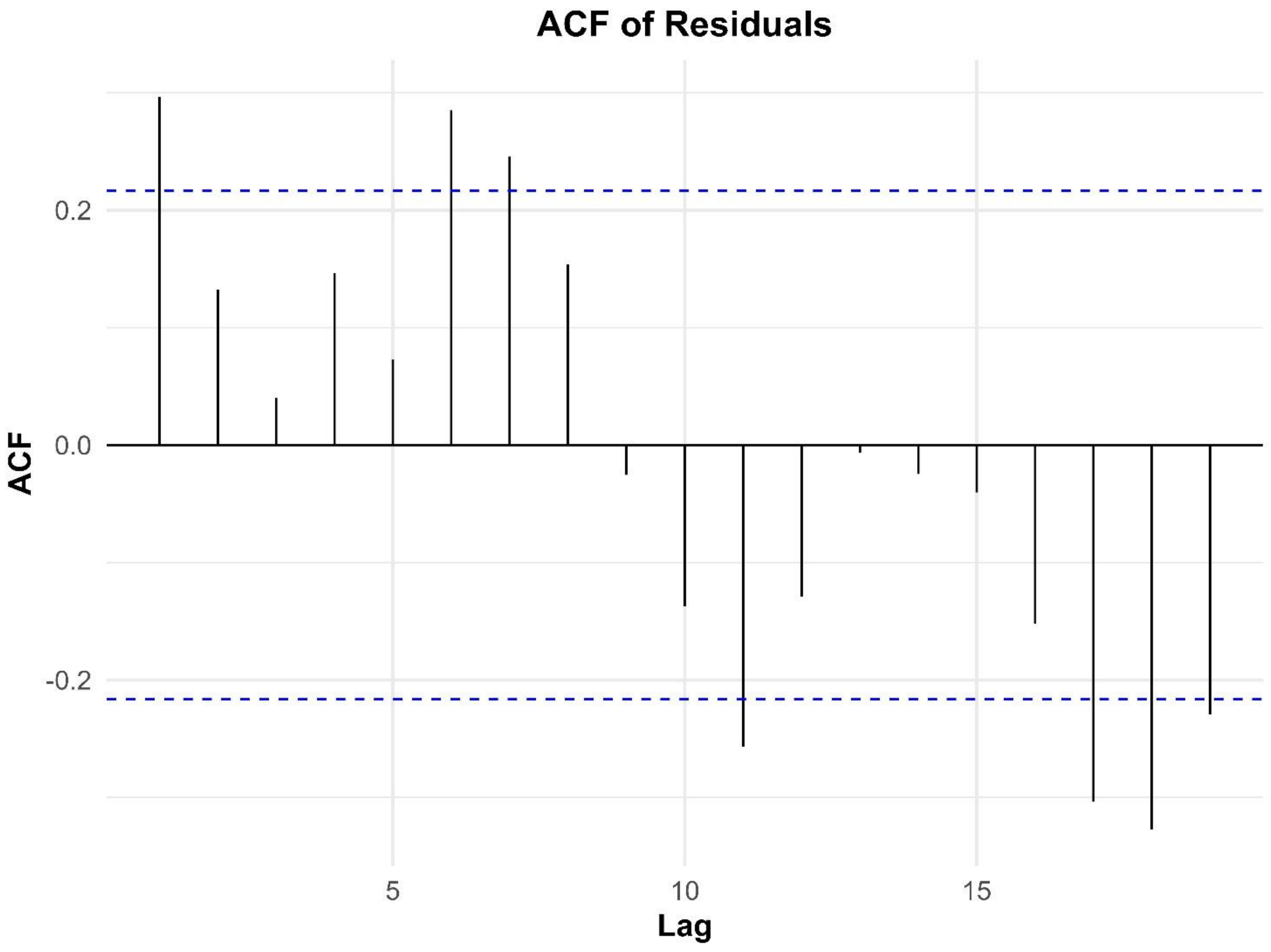

Supplementary Figure 26. Cumulative Impact Plot of the Intervention Over Time (Antidepressant Prescriptions).

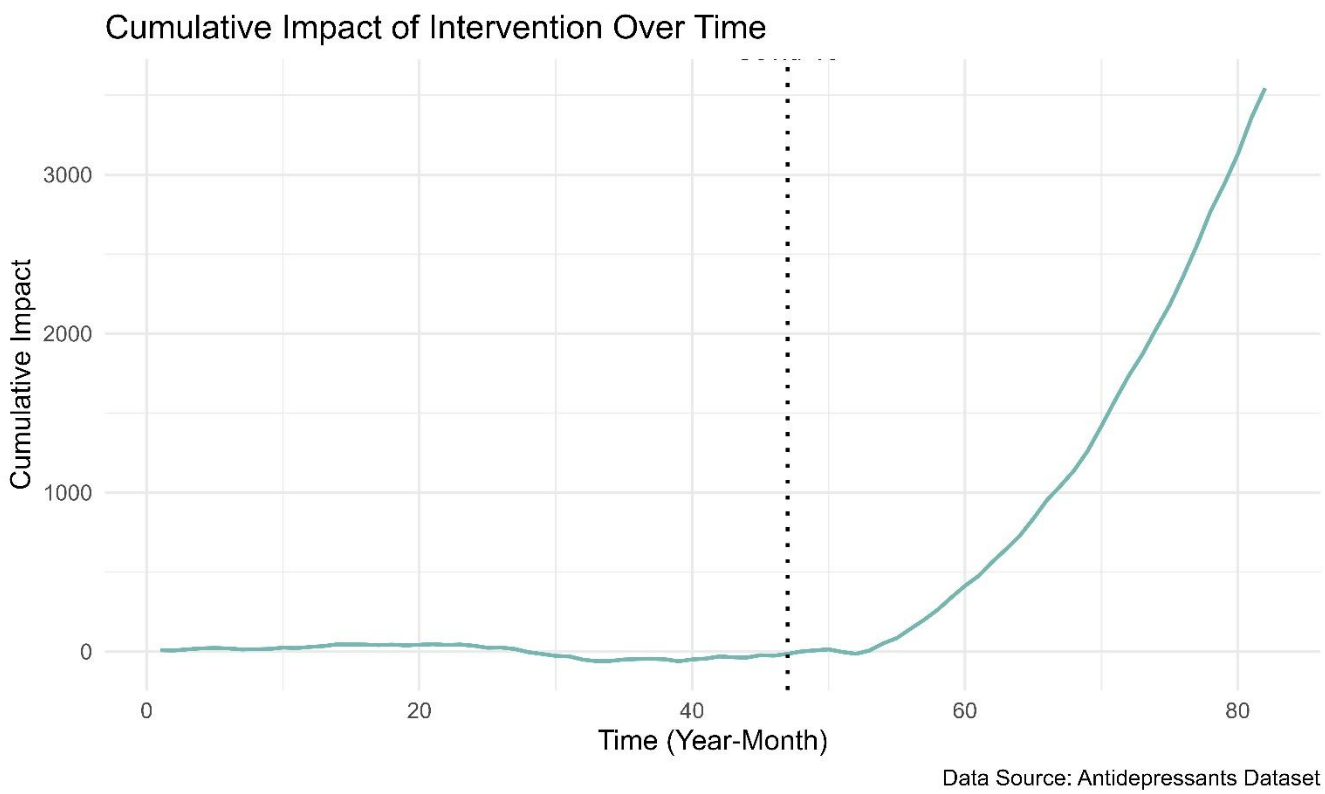

Supplementary Figure 27. Cumulative Impact Plot of the Intervention Over Time (Antipsychotic Prescriptions).

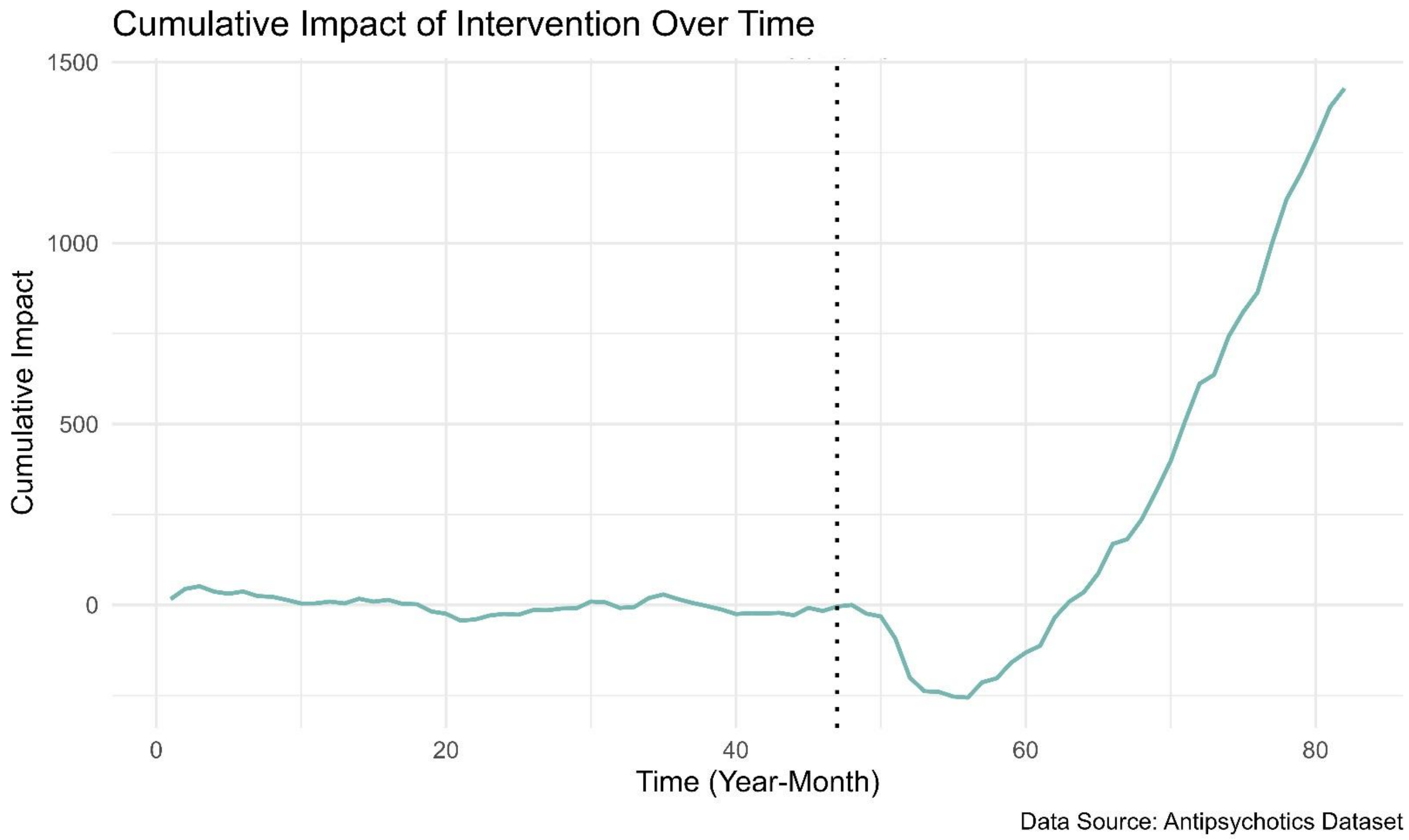

Supplementary Figure 28. Cumulative Impact Plot of the Intervention Over Time (Anxiolytic Prescriptions).

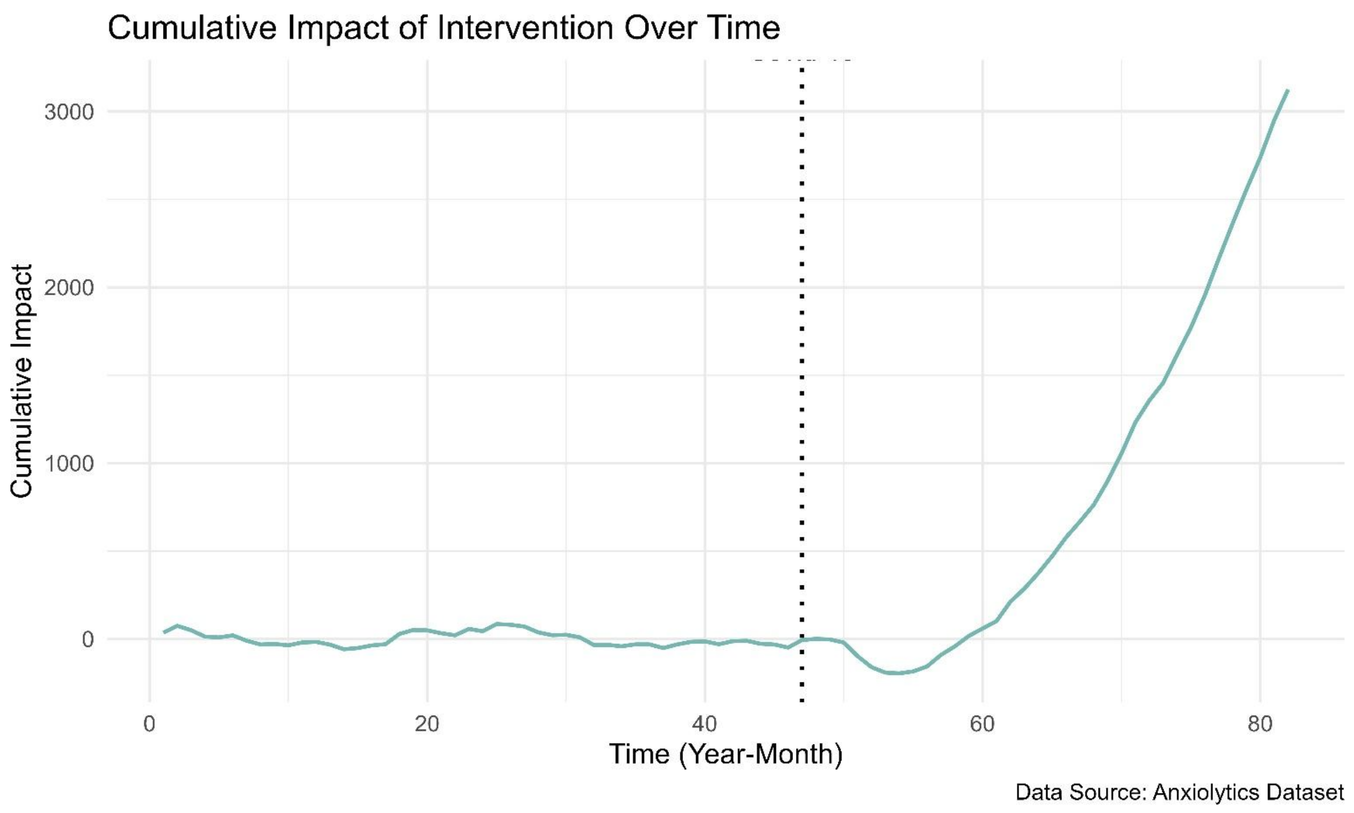

Supplementary Figure 29. Cumulative Impact Plot of the Intervention Over Time (Hypnotic and Sedative

Prescriptions).

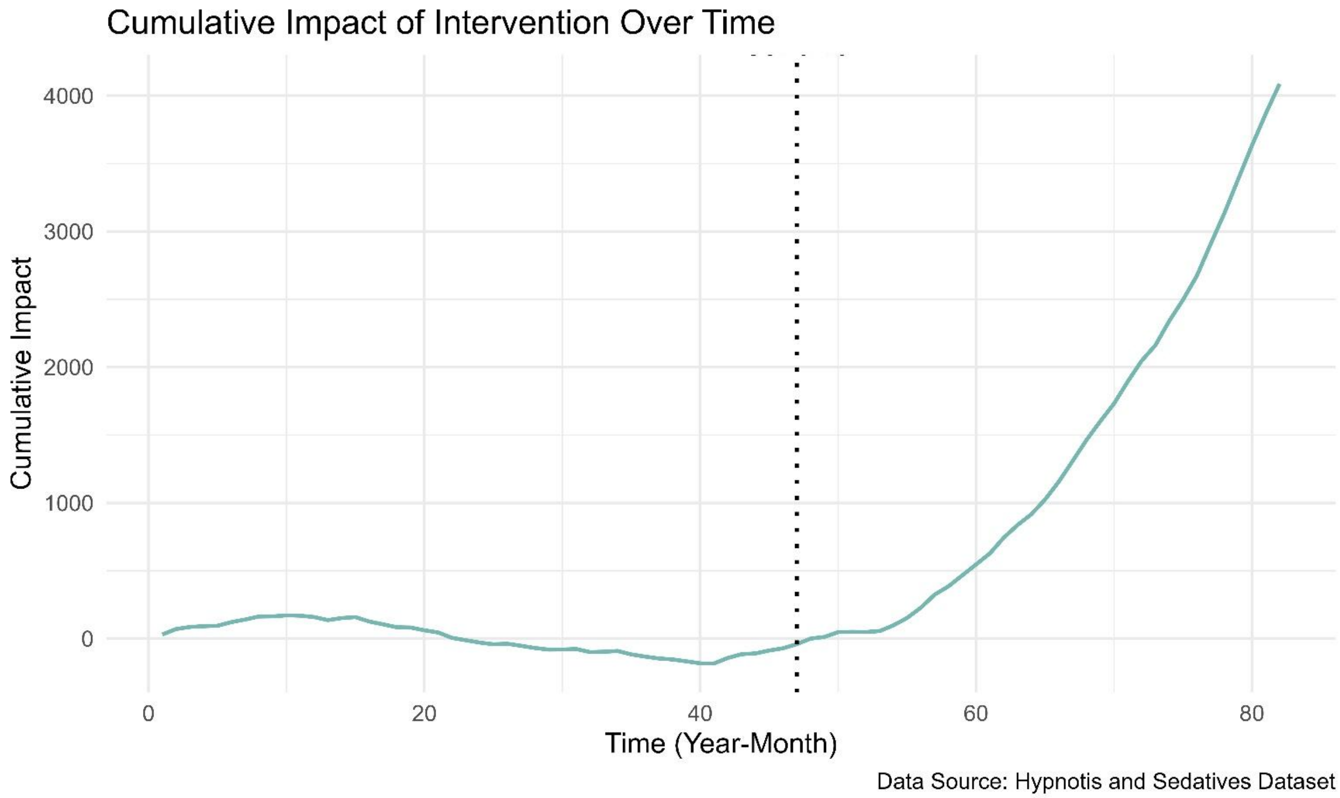

Supplementary Figure 30. Cumulative Impact Plot of the Intervention Over Time (Psychostimulant Prescriptions).

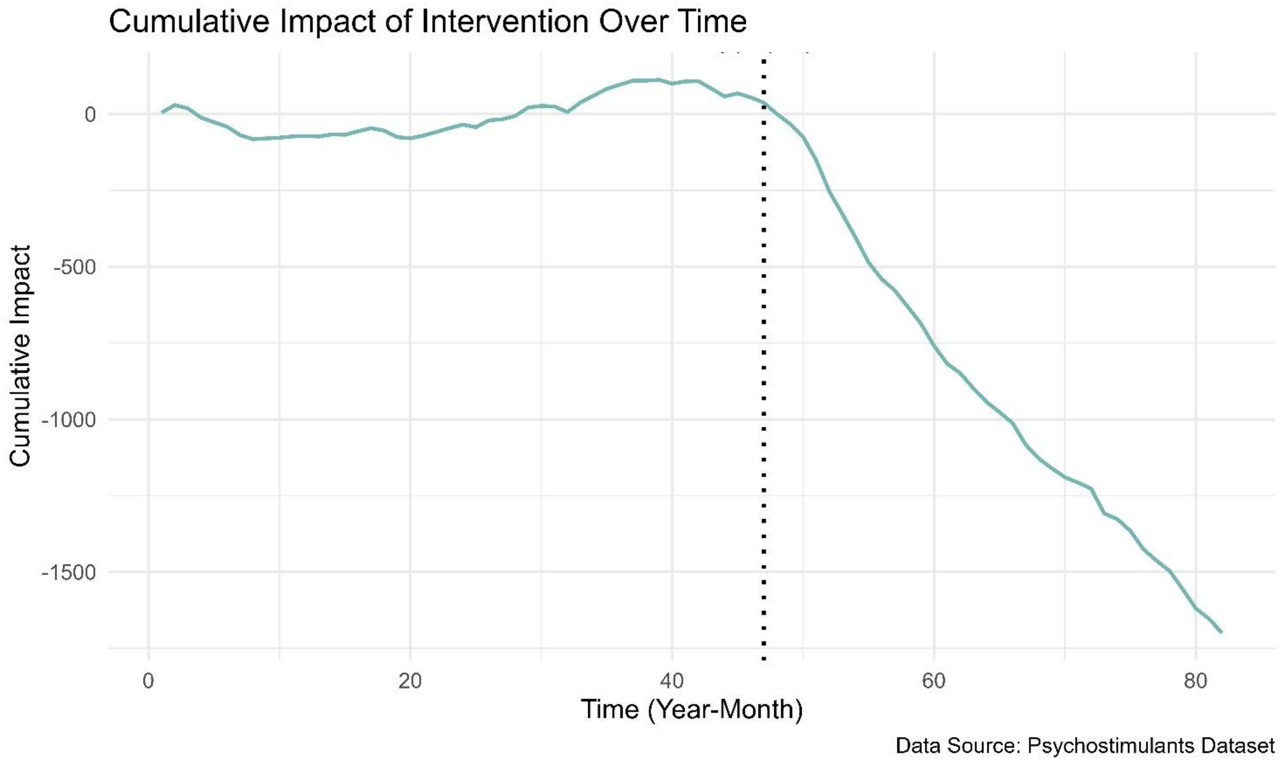

Supplementary Figure 31. Residuals Over Time (Antidepressant Prescriptions).

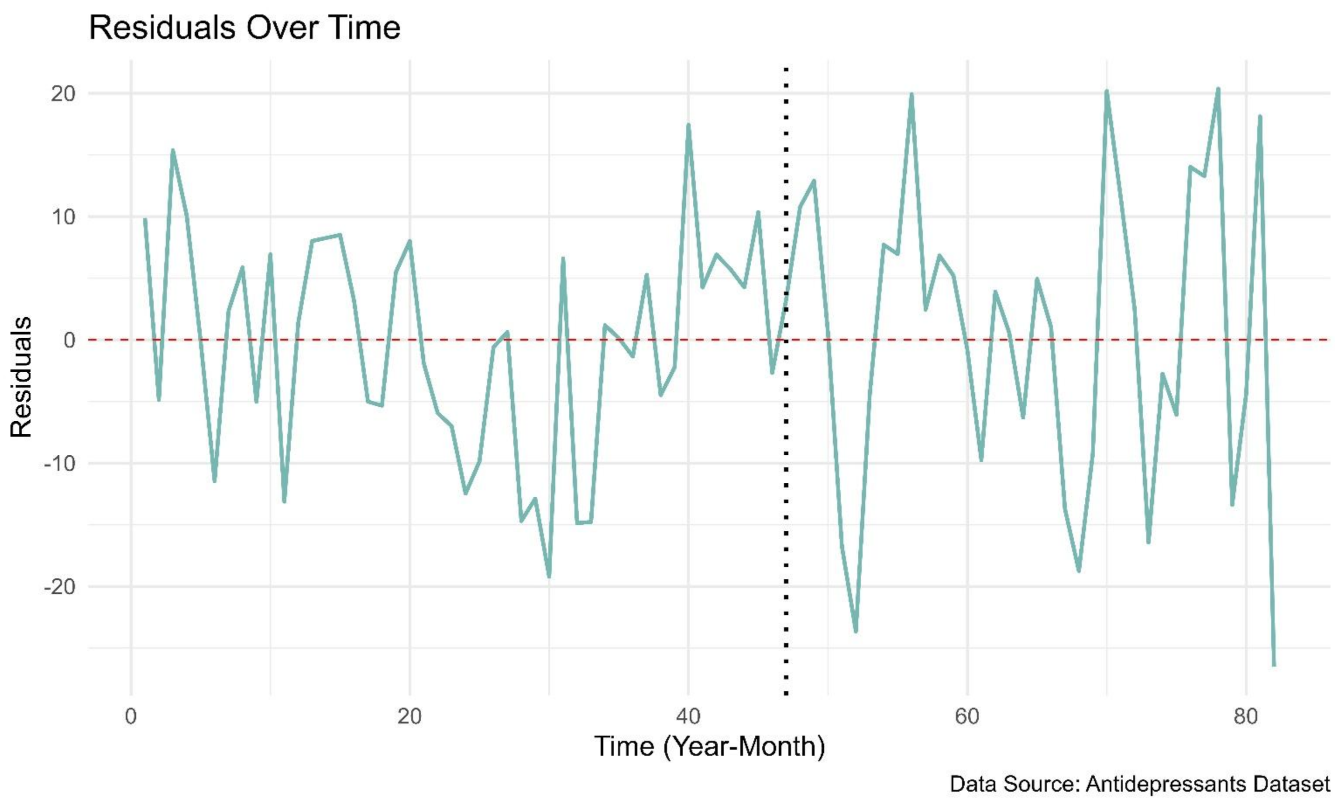

Supplementary Figure 32. Residuals Over Time (Antipsychotic Prescriptions).

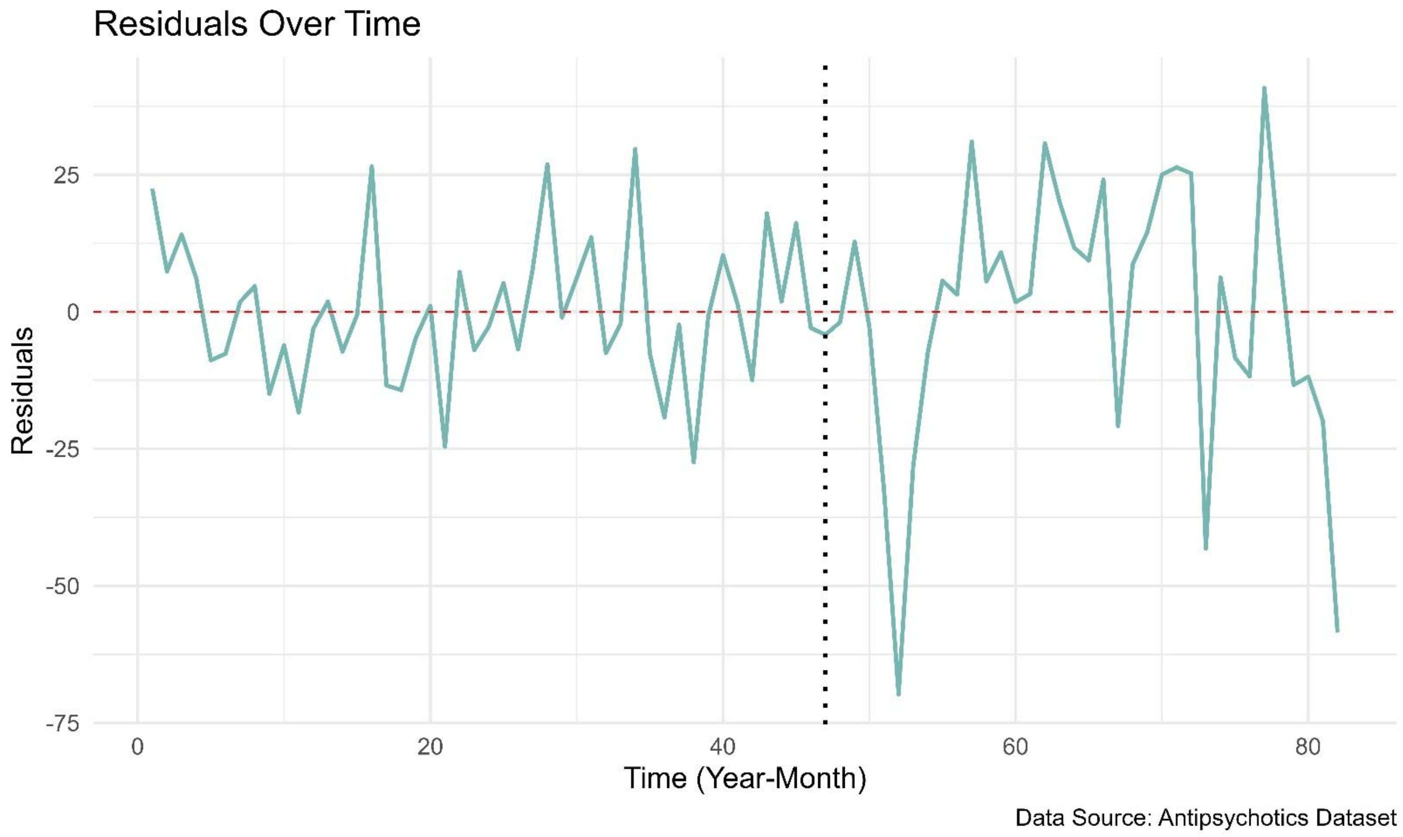

Supplementary Figure 33. Residuals Over Time (Anxiolytic Prescriptions).

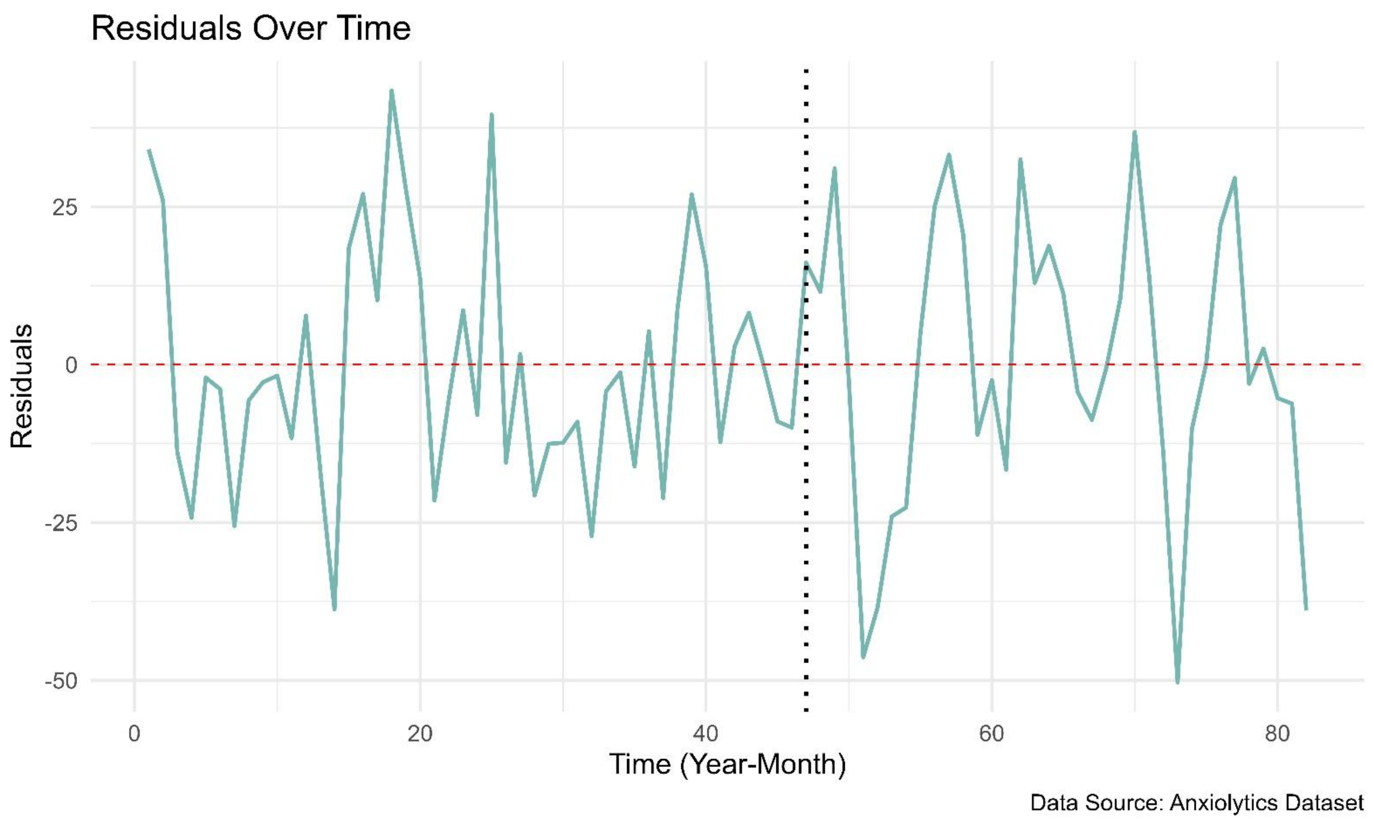

Supplementary Figure 34. Residuals Over Time (Hypnotic and Sedative Prescriptions).

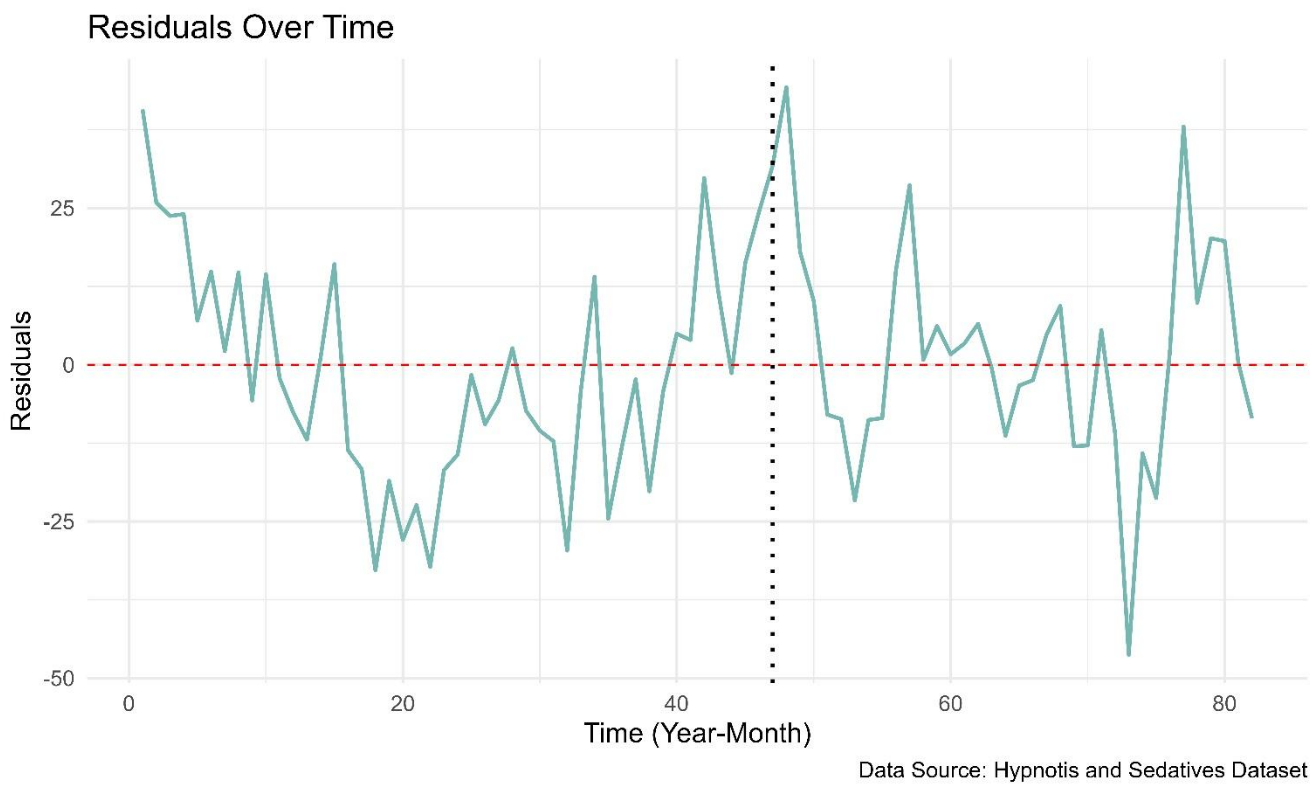

Supplementary Figure 35. Residuals Over Time (Psychostimulant Prescriptions).

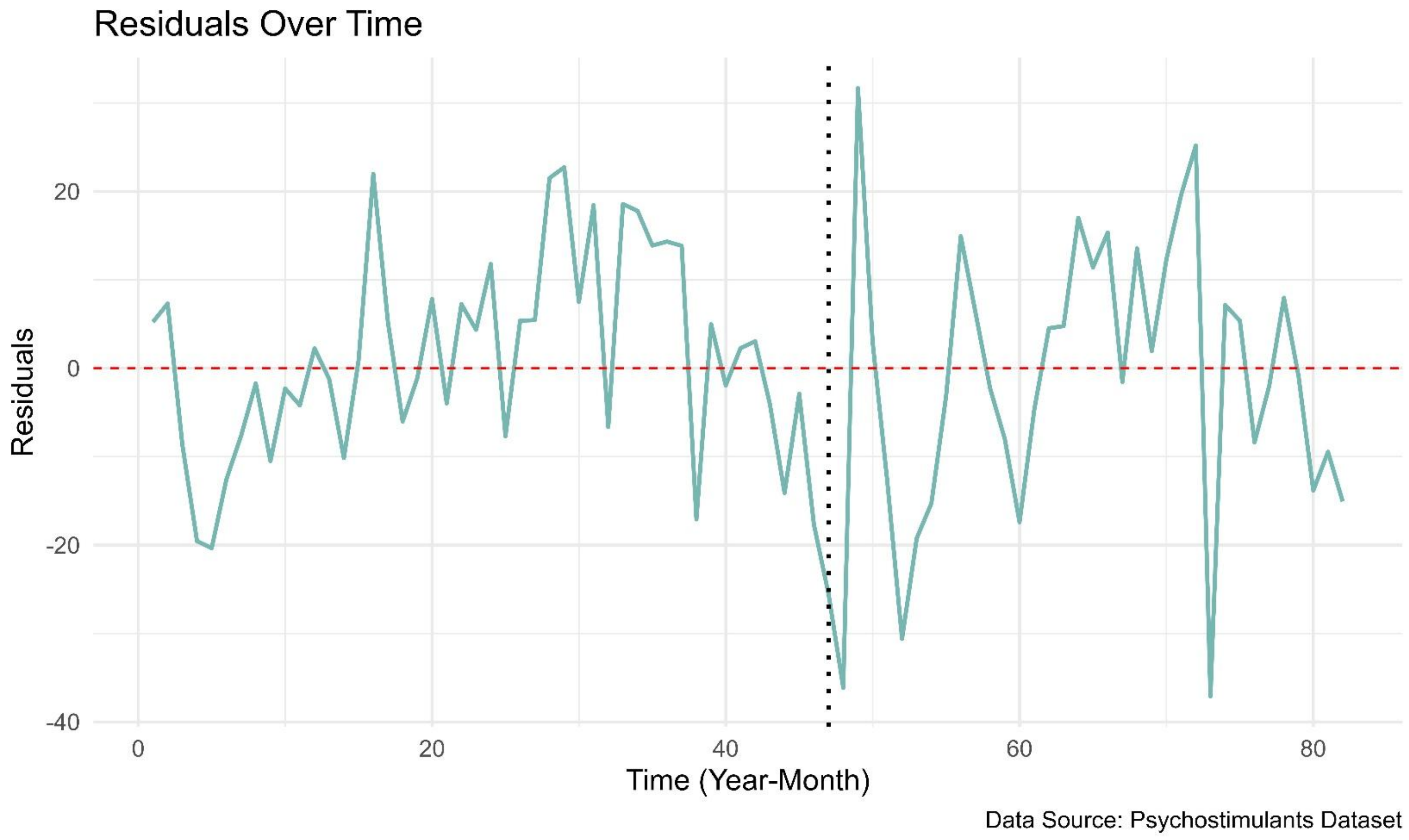

Supplementary Figure 36. Segmented Regression Plot (Antidepressant Prescriptions).

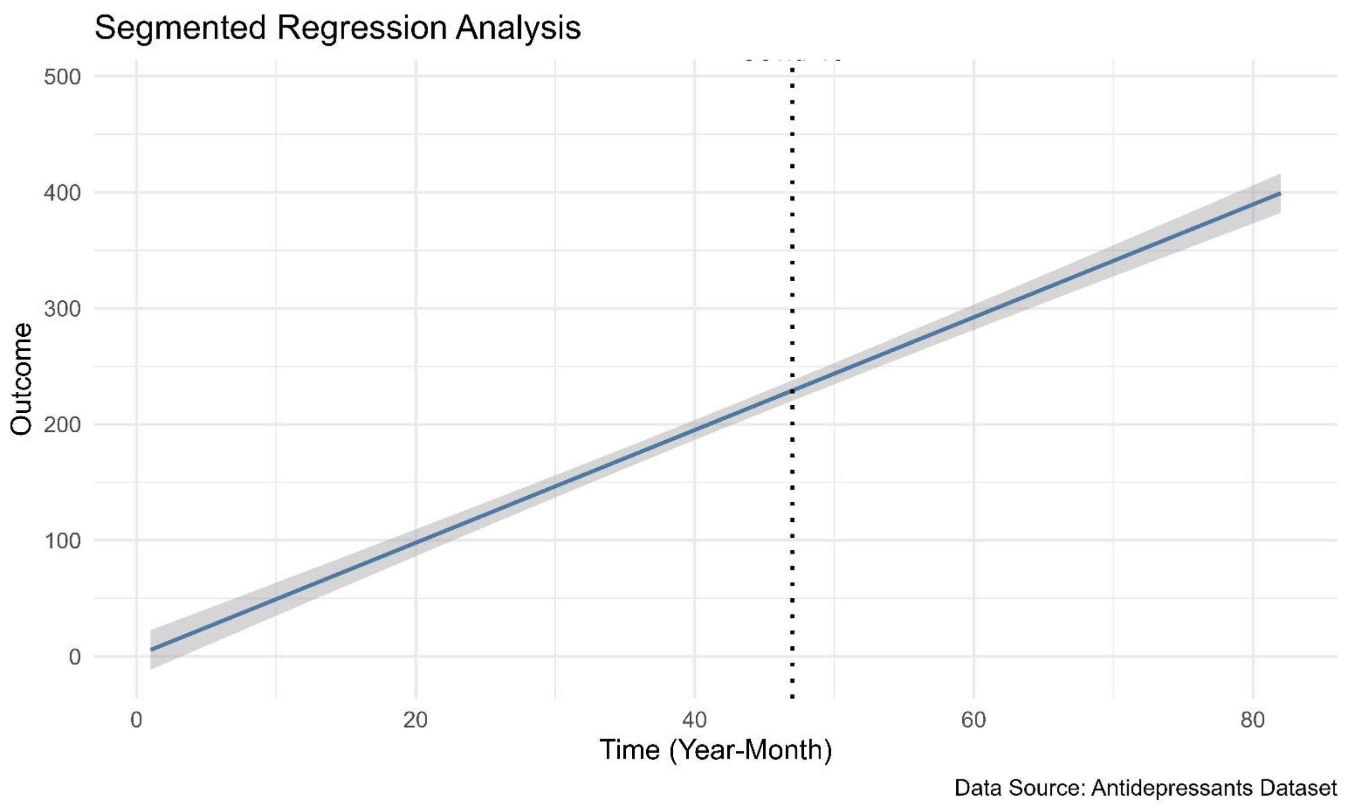

Supplementary Figure 37. Segmented Regression Plot (Antipsychotic Prescriptions).

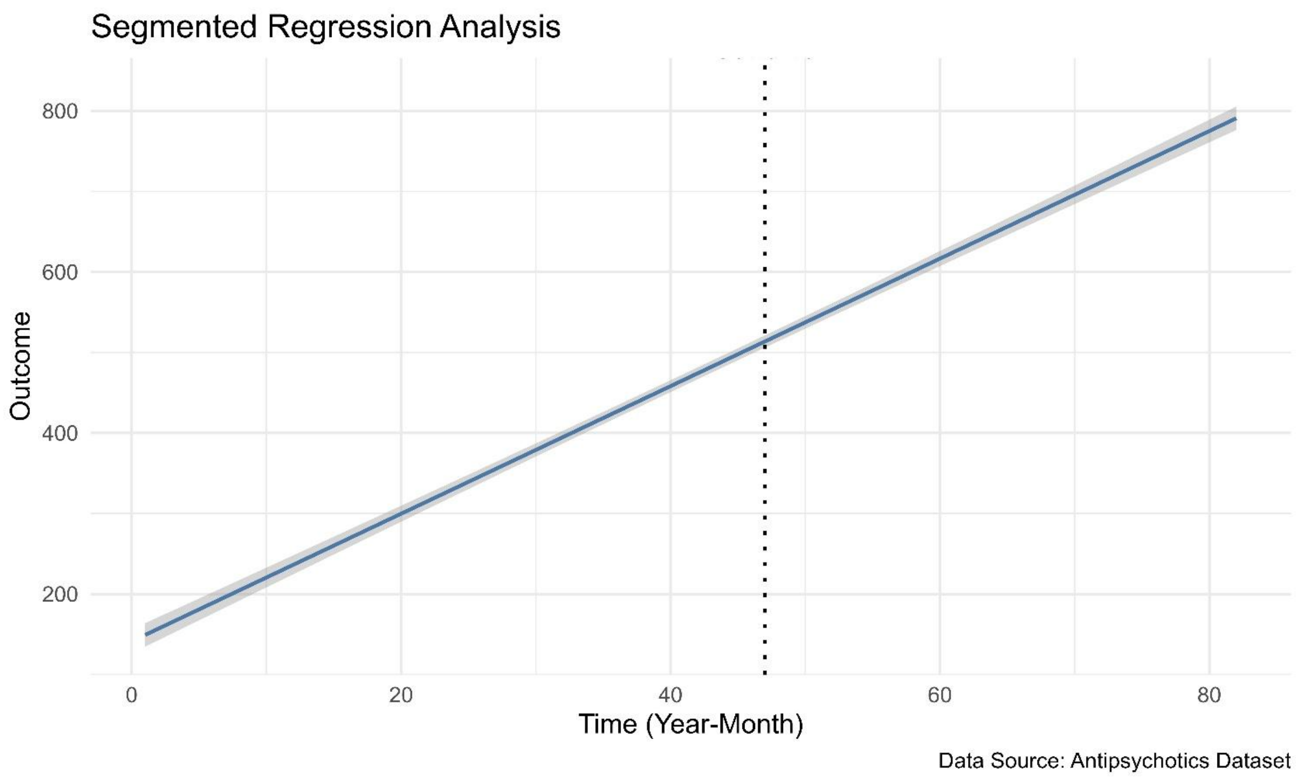

Supplementary Figure 38. Segmented Regression Plot (Anxiolytic Prescriptions).

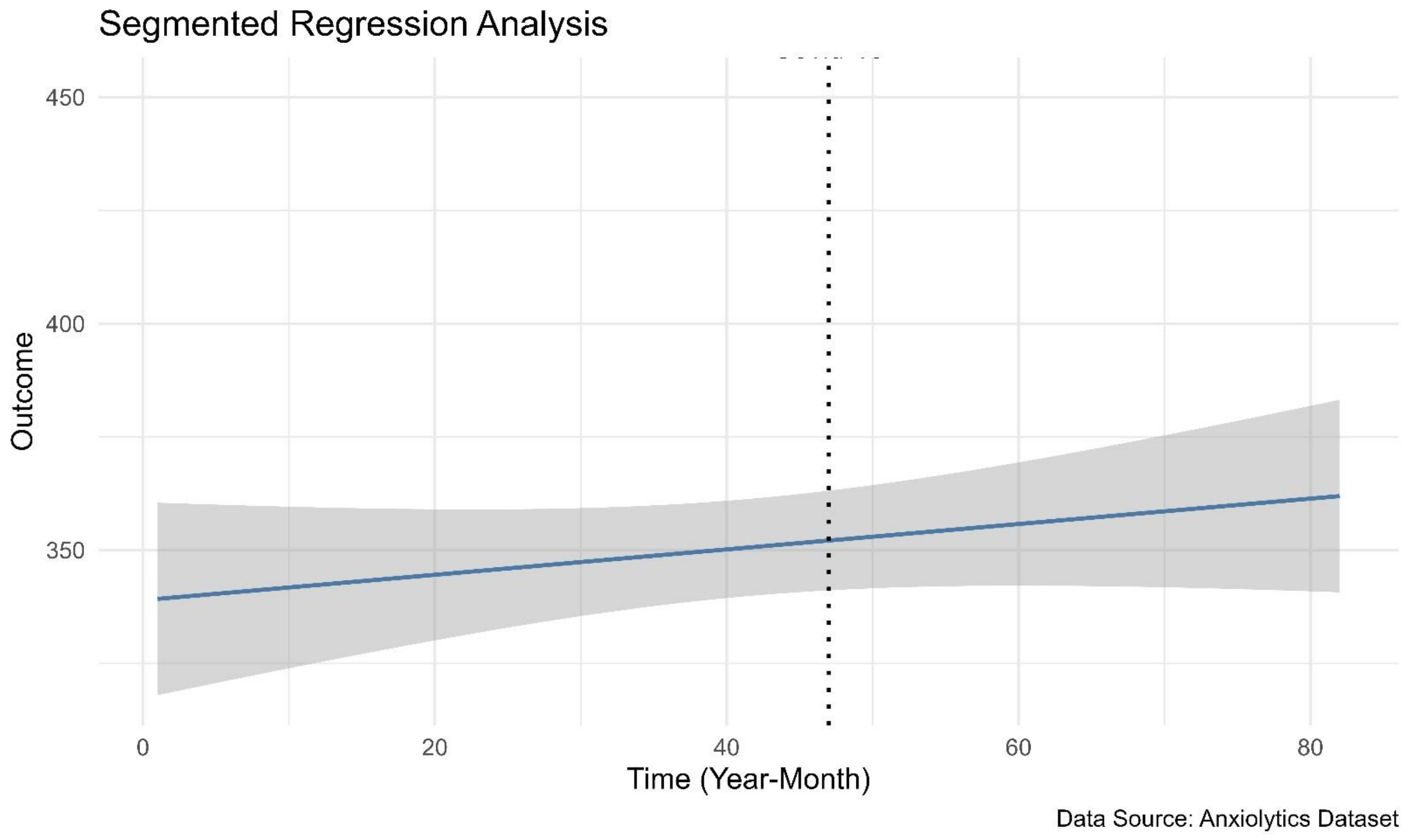

Supplementary Figure 39. Segmented Regression Plot (Hypnotic and Sedative Prescriptions).

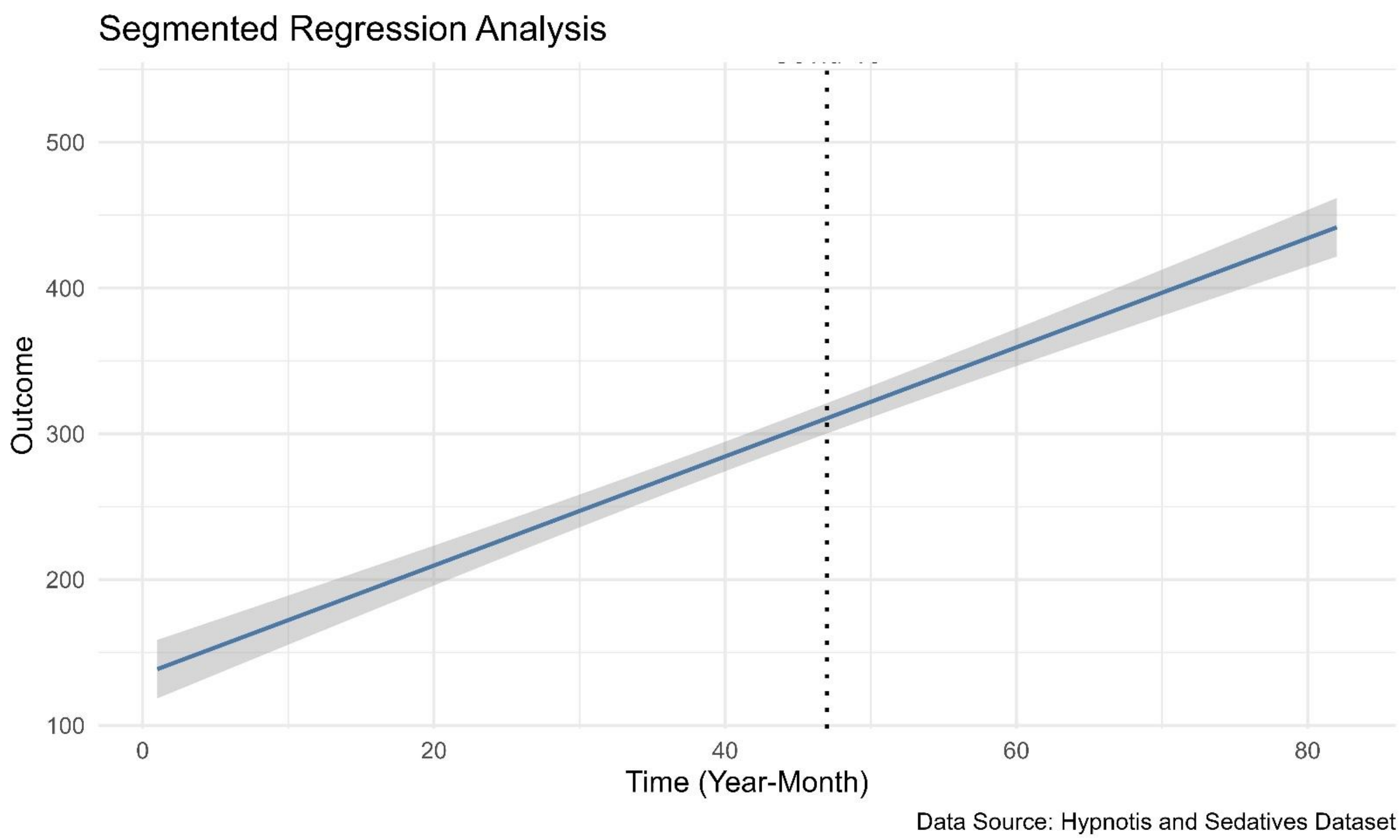

Supplementary Figure 40. Segmented Regression Plot (Psychostimulant Prescriptions).

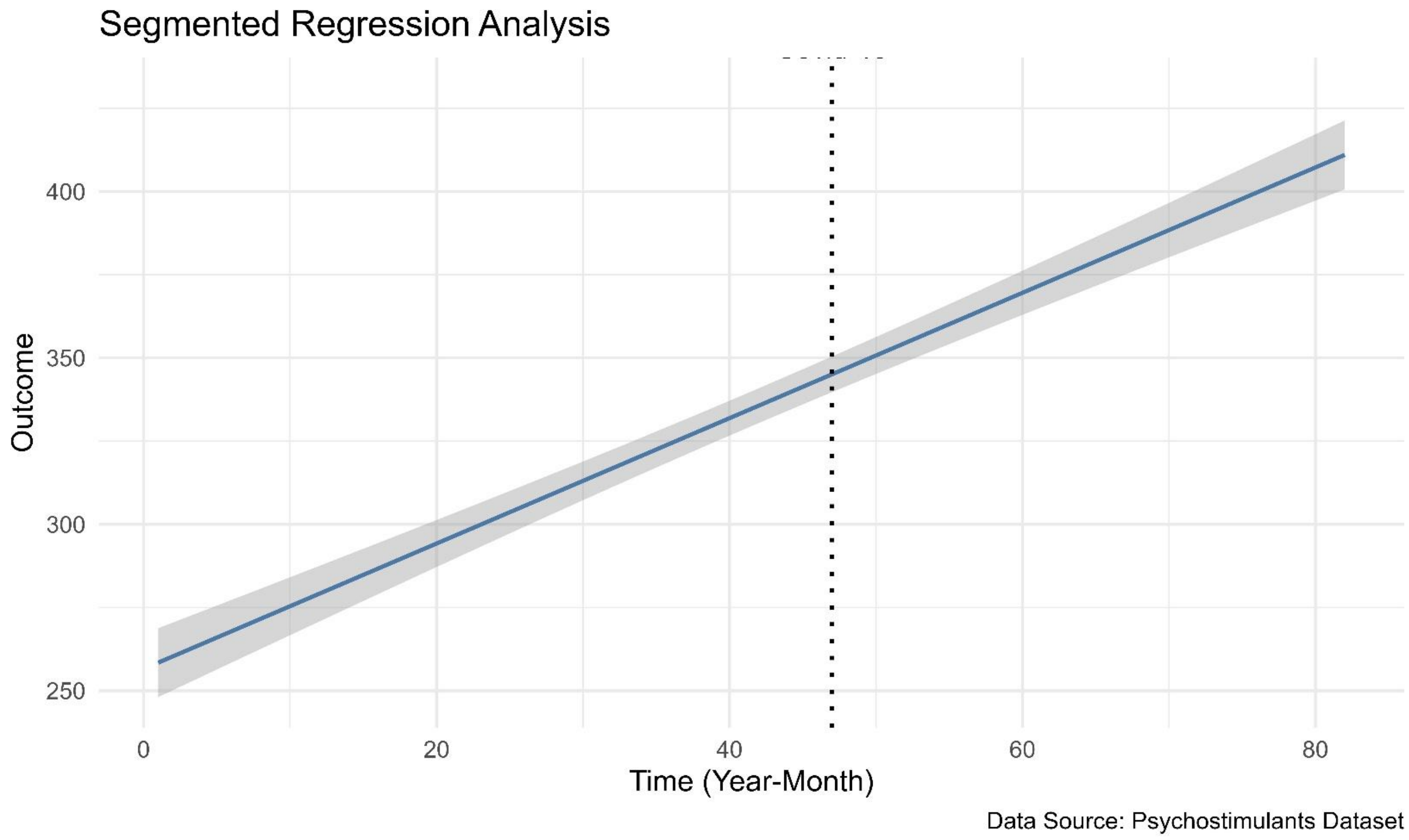

Version 3.00 - 2025-07-03

Open Source Licenses

This analysis exclusively utilizes open-source code. All analysis and graphing scripts provided here are governed by the license terms of the respective R packages employed in this work.

Copyright (c) <2024> <Wenbo Huang (wenbohuang@g.ecc.u-tokyo.ac.jp), Hiroki Matsui (ptmatsui-tky@g.ecc.u-tokyo.ac.jp)>

Permission is hereby granted, free of charge, to any person obtaining a copy of this software and associated documentation files (the "Software"), to deal in the Software without restriction, including without limitation the rights to use, copy, modify, merge, publish, distribute, sublicense, and/or sell copies of the Software, and to permit persons to whom the Software is furnished to do so, subject to the following conditions:

The above copyright notice and this permission notice shall be included in all copies or substantial portions of the Software.

THE SOFTWARE IS PROVIDED "AS IS", WITHOUT WARRANTY OF ANY KIND, EXPRESS OR IMPLIED, INCLUDING BUT NOT LIMITED TO THE WARRANTIES OF MERCHANTABILITY, FITNESS FOR A PARTICULAR PURPOSE AND NONINFRINGEMENT. IN NO EVENT SHALL THE AUTHORS OR COPYRIGHT HOLDERS BE LIABLE FOR ANY CLAIM, DAMAGES OR OTHER LIABILITY, WHETHER IN AN ACTION OF CONTRACT, TORT OR OTHERWISE, ARISING FROM, OUT OF OR IN CONNECTION WITH THE SOFTWARE OR THE USE OR OTHER DEALINGS IN THE SOFTWARE.
